# Differential pre-mRNA Splicing Alters the Transcript Diversity of *Helitrons* Between the Maize Inbred Lines

**DOI:** 10.1534/g3.115.018630

**Published:** 2015-06-12

**Authors:** Brian T. Lynch, Tara L. Patrick, Jennifer J. Moreno, Amy E. Siebert, Katarina M. Klusman, Donya N. Shodja, L. Curtis Hannah, Shailesh K. Lal

**Affiliations:** * Department of Biological Sciences, Oakland University, Rochester, Minnesota 48309-4401; † Department of Horticultural Sciences and Program in Plant Molecular and Cellular Biology, University of Florida, Gainesville, Florida 32611

**Keywords:** *Helitron*, alternative splicing, gene evolution, transposable element

## Abstract

The propensity to capture and mobilize gene fragments by the highly abundant *Helitron* family of transposable elements likely impacts the evolution of genes in *Zea mays*. These elements provide a substrate for natural selection by giving birth to chimeric transcripts by intertwining exons of disparate genes. They also capture flanking exons by read-through transcription. Here, we describe the expression of selected *Helitrons* in different maize inbred lines. We recently reported that these *Helitrons* produce multiple isoforms of transcripts in inbred B73 via alternative splicing. Despite sharing high degrees of sequence similarity, the splicing profile of *Helitrons* differed among various maize inbred lines. The comparison of *Helitron* sequences identified unique polymorphisms in inbred B73, which potentially give rise to the alternatively spliced sites utilized by transcript isoforms. Some alterations in splicing, however, do not have obvious explanations. These observations not only add another level to the creation of transcript diversity by *Helitrons* among inbred lines but also provide novel insights into the *cis*-acting elements governing splice-site selection during pre-mRNA processing.

*Helitrons* represent a novel family of highly abundant transposable elements in the genomes of species representing all eukaryotic kingdoms (Kapitonov and Jurka 2001; Lal *et al.* 2003; Pritham and Feschotte 2007; Yang and Bennetzen 2009; Lal *et al.* 2009a,b). An autonomous *Helitron* is proposed to encode DNA helicase and a replication initiator protein. These are similar to bacterial transposons, which transpose via a rolling circle mechanism (Tavakoli *et al.* 2000; Kapitonov and Jurka 2001). Typically, unlike other known transposable elements, *Helitrons* do not contain repeats of their terminal ends and do not duplicate the host sequence upon insertion (Kapitonov and Jurka 2001). Rather, they insert between host dinucleotide AT in the vast majority of cases, and bear conserved TC and CTRR nucleotides at their 5′ and 3′ terminal ends, respectively.

Although *Helitrons* are proposed to multiply via strand replacement and synthesis, similar to the copy-and-paste mechanism, recent polymerase chain reaction (PCR)-based detection of their somatic excisions in maize indicates these elements also can transpose via a cut-and-paste mechanism (Li and Dooner 2009). Despite their importance, unequivocal genetic or biochemical evidence of their activity and movement to date remains elusive in any species. The presence of nearly identical copies of long *Helitrons* inserted in different regions of the genome points to their recent mobility in the maize genome. Our earlier report of two maize mutants caused by the recent insertion of *Helitrons* provided the first genetic evidence of their mobility in the present day maize genome (Lal *et al.* 2003; Gupta *et al.* 2005). Recently, *Helitrons* were reported to be the most abundant DNA transposon in the maize genome. An improved version of the computational-based searches estimates the presence of more than 31,000 *Helitrons*. These constitute >6% of the total maize genome (Xiong *et al.* 2014). These *Helitrons* are prolific in capturing thousands of gene pieces and mobilizing them into different regions of the genome (Du *et al.* 2009; Yang and Bennetzen 2009). Gene piece capture appears random, and the advantage of this to the host or the element remains unclear. The captured gene pieces display varying degrees of sequence similarity to their putative progenitors. It remains unclear whether this reflects the evolutionary time of their capture or natural selection. Several contrasting mechanisms for gene capture by *Helitrons* have been proposed but each lacks supporting experimental evidence (Feschotte and Wessler 2001; Brunner *et al.* 2005; Gupta *et al.* 2005; Kapitonov and Jurka 2007; Lal *et al.* 2009a,b). In addition to coding regions, *Helitrons* have mediated massive movement of a diverse collection of host sequences, including promoters, poly-A addition sites and binding sites for various regulatory proteins across the genome. Emerging reports indicate that in addition to coding regions, *Helitrons* also have captured and multiplied various host regulatory sequences that display functionality. These point to their possible important roles in physiological processes and adaptation of organisms to their environment (Ellison and Bachtrog 2013; Fu *et al.* 2013; Han *et al.* 2013; Capriglione *et al.* 2014; Thomas *et al.* 2014).

*Helitron*-captured gene pieces represent dead remnants of their progenitors in the majority of cases. However, they are sometimes transcribed, giving birth to eclectic transcripts, which fuse coding regions of different genes. These may evolve into new genes with novel domains and functions after selection (Lal *et al.* 2003; Brunner *et al.* 2005; Morgante *et al.* 2005). The location of the promoters driving the expression of the captured genes remains elusive in the vast majority of cases (Brunner *et al.* 2005; Morgante *et al.* 2005; Jameson *et al.* 2008). We recently demonstrated that alternative splicing and read-through transcription dramatically augment the transcript diversity of *Helitron*-captured genes in maize (Barbaglia *et al.* 2012). These provide a potential substrate for natural selection. The molecular basis defining the aberrant alternative splicing of *Helitron*-captured genes is not apparent. Perhaps the close proximity of different gene sequences and mutations within the elements interfere with the proper recognition of their wild-type splice sites. Mutations affecting splice-site selection from a distance have been reported in both plants and animals (McNellis *et al.* 1994; Marillonnet and Wessler 1997; Lal *et al.* 1999b).

Here, we performed expression analysis of selected *Helitrons* in different maize inbred lines. We previously reported alternatively spliced expression of these *Helitrons* in inbred B73 (Barbaglia *et al.* 2012). Our data indicate that these specific *Helitrons* inserted into these respective sites before the divergence of the inbreds examined here are expressed in both etiolated shoots and roots in other inbred lines. However, the pattern of alternatively spliced transcripts differed dramatically among the inbred lines. The comparison of the *Helitrons* and their transcripts among the inbred lines identified key polymorphisms potentially affecting the differential usage of splice sites among inbred lines. These observations not only add another degree of complexity to the diversity of *Helitron*-captured genes among inbred lines for potential natural selection, but also provide novel insights into the mechanism of splice site selection during pre-mRNA processing in plants.

## MaterialS and Methods

### Plant material

The maize inbred lines B73, HP301, OH7B, and Tzi8 were obtained from Maize Genetics Cooperative Stock Center, University of Illinois. Plants were grown in the greenhouse or in the field at the University of Florida/Institute of Food and Agriculture Sciences facility in Citra, FL.

### Genomic and reverse transcription (RT)-PCR analysis

Following the protocol provided by the manufacturer, we extracted genomic DNA from kernel tissue using the DNeasy Plant Mini Kit (QIAGEN). The PCR amplification of *Helitrons Hel1-331*, *Hel1-332*, and *Hel1-333* in inbred lines HP301, OH7B, and Tzi8 was achieved by use of the same primer pairs used for RT-PCR expression analysis of these *Helitrons* in maize inbred line B73 described previously (Barbaglia *et al.* 2012), except amplification of *Helitron Hel1-333* was performed with two sets of overlapping PCR primers. The primer pairs H33-1F (5′-GCGTTCTGCCGTTAGACAAT-3′) and H33-13R (5′-AGGGTGACCAAAGAGCAAGT-3′) spanning positions 51,979−52,018 bp and 58,508−58,527bp, respectively, of HTGS clone (gi: 373839194) are complementary to exon 1 and exon 13 of predicted gene structure of the captured gene by *Hel1-333*. Similarly, primer pairs H33-13F (5′-ACCAGAGACGGGAGGTCT-3′) and H33-14R (5′-TCATGCCCTTTACACTTGAT-3′) span positions 58,468−58,487 bp and 60,207−60,226 bp and complementary to exon 13 and exon 14, respectively. Primers designed on B73 inbred sequence failed to PCR amplify the border junction sequence of *Hel1-331* and *Hel1-332* in various inbred lines (data not presented). This may either be to the result of different insertion sites of *Hel1-331* and *Hel1-332* in different inbreds or the polymorphisms at the binding sites of the primers between the inbred lines. In contrast, the successful PCR amplification and sequencing of the products using the primers complementary to exon 14, which lies outside of *Hel1-333* strongly, indicates this *Helitron* is inserted at the same site in the inbred lines.

Total RNA was extracted from 3-d-old, dark-grown etiolated roots and shoots of various inbred lines using Invitrogen’s TRIzol reagent. Resultant RNA was subjected to RT-PCR analysis using Superscript First Strand Synthesis kit (Invitrogen) using the same primers reported earlier for the expression analysis for *Hel1-331* and *Hel1-332* in inbred B73 (Barbaglia *et al.* 2012). However, a different set of primer pairs H33E1F (5′-GAGGCCACCGACACATATTC-3′) and H33E14R (5′-GCTTTCCTGCTCACACCTTC-3′), spanning positions 51,865–51,855 bp and 60,107–60,127 bp of the HTGS clone (gi: 373839194) was used for RT-PCR analysis of *Helitron Hel1-333* in inbred lines HP301, OH7B, and Tzi8. These primers hybridize to exon 1 and exon 14 of *Hel1-333* (Barbaglia *et al.* 2012). RT-PCR was performed using High Fidelity Platinum Taq Polymerase (Invitrogen) with at least three different RNA extracts from each inbred line to test the reproducibility of banding pattern of PCR products on agarose gels. The resultant PCR products from several different RT-PCRs were resolved on gel, purified, cloned into TOPO TA vectors (Invitrogen), and sequenced in both directions by the University of Florida Interdisciplinary Center for Biotechnology Research DNA Sequencing Core Laboratory. Annotation and determination of splices sites were done by manual examination of the splice alignment of the *Helitrons* with their cognate RT-PCR amplified transcripts using the computer software GeneSeqer and SplicePredictor, respectively (Usuka and Brendel 2000a; Usuka *et al.* 2000b). The multiple sequence alignment to determine the polymorphic regions between the inbred lines was performed using the programs Multalin and ClustalW (http://multalin.toulouse.inra.fr/multalin/multalin.html) (Corpet 1988; Larkin *et al.* 2007).

## Results

### Differential splicing of *Helitron*-transcribed genes among various maize inbred lines

**Figure 1:**
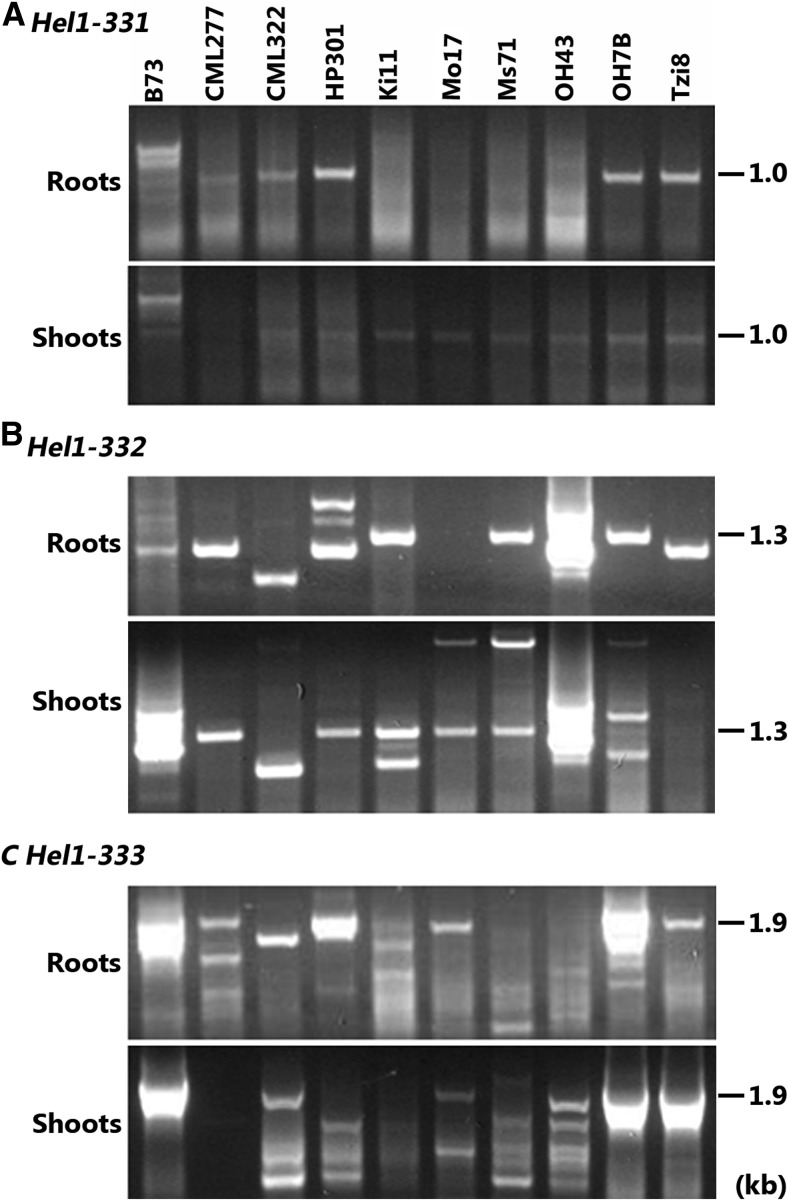
Expression analysis of *Helitrons* in different maize inbred lines. (A−C) Reverse-transcription polymerase chain reaction products extracted from roots (top) and shoots (bottom) of various inbred lines using primers specific *for Helitrons*, *Hel1-331*, *Hel1-332*, and *Hel1-333*, respectively. These *Helitron*-specific primers were based on the inbred B73 sequence described previously (Barbaglia *et al.* 2012). The inbred lines are shown at top of the panel. Molecular weight markers in kilobases are displayed on the left of each panel.

We recently reported the expression analysis of several *Helitrons* in maize inbred line B73 (Barbaglia *et al.* 2012). The elements analyzed were selected initially from expressed sequence tag evidence of captured gene transcription. Our data indicated that these *Helitrons* produced multiple transcripts via differential selection of splice sites during pre-mRNA processing and read-through transcription. Because alternative splicing and *Helitrons* are both viewed as potentially important forces in the processes of evolution, we examined the expression of *Helitrons Hel1-331*, *Hel1-332*, and *Hel1-333* in different maize inbred lines. Total RNA from etiolated root and shoot tissues from inbred lines B73, CML277, CML322, HP301, Ki11, Mo17, Ms71, OH43, OH7B, and Tzi8 was subjected to RT-PCR analysis using the same primers for each *Helitron* reported previously in B73 (Barbaglia *et al.* 2012). The resultant amplified products were resolved on an agarose gel and stained with ethidium bromide. As displayed in Figure 1, the profile of RT-PCR products for each *Helitron* in the other inbreds dramatically differed from B73. For example, several RT-PCR products from both roots (Figure 1A) and shoots (Figure 1B) were derived from *Helitron Hel1-331* in inbred B73. In contrast, only a single product of approximately 1.0kb was amplified from roots in inbred lines CML277, CML322, HP301, OH7B, and Tzi8 and shoots in inbred lines CML322, HP301, Ki11, Mo17, Ms71, OH43, OH7B, and Tzi8 (Figure 1, A and B).

As previously reported, multiple transcripts were derived from *Hel1-332* in roots and shoots of inbred B73 (Figure 1, A and B) (Barbaglia *et al.* 2012). However, the profile of the amplified products differed considerably among the other inbred lines. For example, a single product of ∼1.1 kb was amplified in both roots and shoots of inbred line CML277. Similarly, a single product of ∼0.9 kb was detected in both roots and shoots of inbred line CML322. In contrast, HP301 produced three RT-PCR products of ∼1.1 kb, ∼1.4 kb, and ∼1.5 kb in roots and a single product of ∼1.3 kb in shoots, whereas inbred Ki11 produced a single product of ∼1.2 kb in roots and two fragments of ∼1.1 kb and 1.3 kb in shoots. No amplification was detected in roots of Mo17, although two fragments of 1.2 kb and 1.8 kb were amplified in shoots. Similar to Mo17, the inbred Ms71 produced two bands of ∼1.4 kb and ∼1.8 kb in shoots, whereas only a single band of ∼1.2 kb in roots. However, in contrast to Mo17, Ms71 amplified a single product of ∼1.2 kb in roots. Multiple products migrating between ∼1.1 kb and 1.4 kb in length were amplified in both shoots and roots of inbred OH43. The inbred OH7B produced one fragment of ∼1.3 kb in roots and three fragments of ∼∼1.1 kb, 1.4 kb, and 1.8 kb in shoots. A single product of ∼1.2 kb was amplified only in the roots of inbred Tzi8.

We reported previously that *Helitron Hel1-333* had captured a flanking exon by read-through transcription and produced seven transcript isoforms by alternative splicing (Barbaglia *et al.* 2012). As displayed in both panels of Figure 1, multiple products spanning between ∼1.7 kb and 2.2 kb in length were amplified in both roots and shoots of inbred line B73. Similar to *Hel1-332*, the amplification profile of *Hel1-333* diverged among different inbred lines. For instance, two major products of ∼1.8 kb and ∼2.1 kb were amplified only in the roots of inbred line CML277. Inbred CML322 amplified a single product of ∼1.9 kb in roots and three distinct fragments of ∼1.2 kb, 1.3 kb, and 1.9 kb in shoots. In contrast, HP301 produced a major product of ∼2.0 kb in roots and several weakly stained bands ranging between ∼1.1 kb and 1.7 kb in length in shoots. However, the sequences of inbred HP301 shoot RT-PCR products displayed no similarity to *Hel1-333* sequence and were considered artifacts of PCR amplification (data not presented). Amplification of inbred Ki11 RNA produced multiple fragments ranging from ∼1.2 kb to ∼2.1 kb in roots and from ∼1.2 kb to ∼1.6 kb in shoots. The inbred Mo17 contained two products of ∼1.6 kb and 1.9 kb in shoots and only one product of ∼1.9 kb in roots. Multiple faintly stained and poorly resolved products ranging between ∼1.0 kb and ∼2.0 kb were amplified from Ms17 roots, and multiple products ranging from ∼1.0 kb to ∼1.8 kb in Ms17 shoot tissue. Poorly resolved, weak fragments ranging between ∼1.2 kb and 1.9 kb also were amplified in both roots and shoots of inbred OH43. Amplicons from RNA of OH7B contained five fragments ranging from ∼1.6 kb to ∼2.1 kb in roots and two fragments of ∼1.6 kb and ∼1.8 kb in shoots, whereas inbred Tzi8 amplified a single product of ∼1.9 kb from roots, and two fragments of ∼1.6 kb and ∼1.8 kb from shoots.

We conclude that *Helitrons Hel1-331*, *Hel1-332*, and *Hel1-333* are expressed in all inbreds reported here. The difference in the RT-PCR profile between inbred lines is caused by differences in splice-site selection during pre-mRNA processing.

We selected inbred lines HP301, OH7B, and Tzi8 to elucidate the molecular basis of differential splicing among maize inbred lines. These lines displayed markedly divergent RT-PCR profiles of alternative splicing for the *Helitrons Hel1-331*, *Hel1-332*, and *Hel1-333* as previously reported (Barbaglia *et al.* 2012).

### A single-donor, splice-site mutation potentially negates alternative splicing of *Hel1-331* transcripts in various maize inbred lines

**Figure 2:**
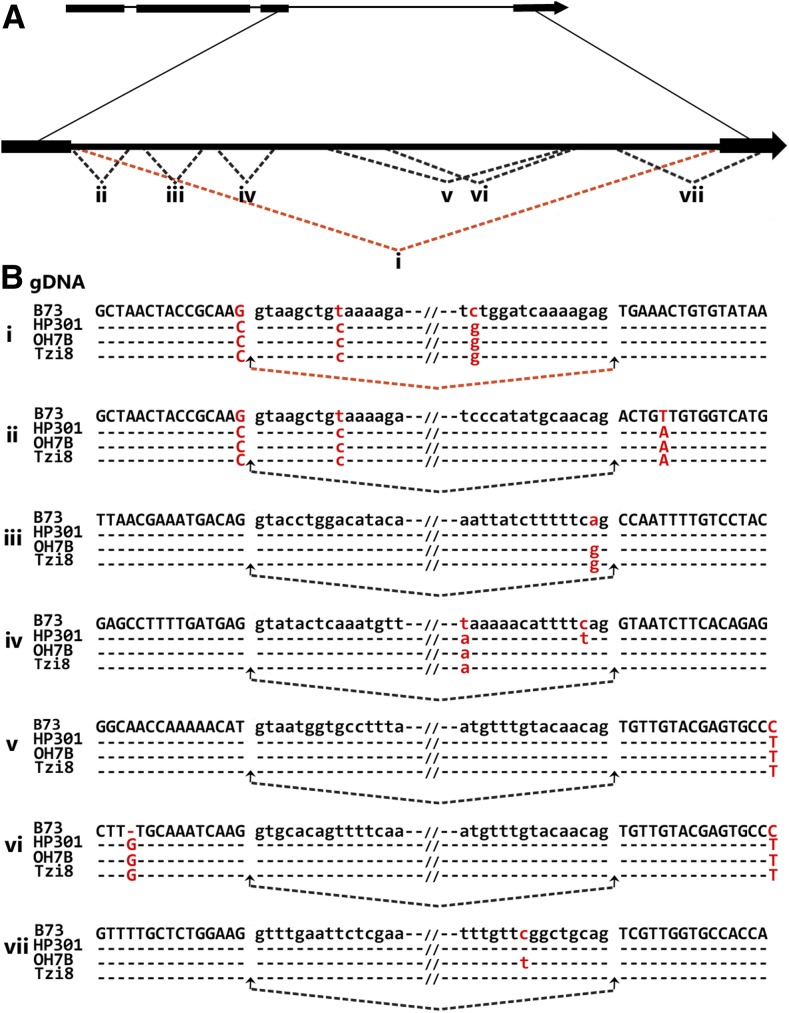
Splicing patterns of *Helitron Hel1-331* in different maize inbred lines. (A) Gene structure of the *Hel1-331* transcript isoforms detected in both roots and shoots of lines B73, HP301, OH7B, and Tzi8. The boxes and lines show exons and introns, respectively. The magnified portion exhibits the positions of the donor and acceptor sites that generate the additional seven *Hel1-331* transcript isoforms in inbred B73 (Barbaglia *et al.* 2012). The dotted lines connect the donor and acceptor sites. The orange line indicates the only transcript found in all four inbred lines. The black lines indicate the isoforms found only in B73. The Roman numerals i−vii mark these splice sites in the different transcript isoforms. (B) Multiple sequence alignments of the splice junctions shown in (A) between the inbred lines. The exons and introns are displayed in upper and lower case letters, respectively. The arrows connected by dotted lines indicate the donor and acceptor sites. The polymorphic nucleotides between the inbred lines are indicated by lettering and color.

To elucidate the molecular basis of the observed differences in the splicing of *Hel1-331* between B73 and other maize inbred lines, we amplified the cognate *Hel1-331* from genomic DNA of lines HP301, OH7B, and Tzi8 using primers designed for amplification in B73 (Barbaglia *et al.* 2012). The resultant product of ∼2.9 kb in length from each inbred line was cloned and sequenced in both directions. Supporting Information, Figure S1 displays the multiple sequence alignment of *Hel1-331* among inbreds B73, HP301, OH7B, and Tzi8. The inbred lines HP301, OH7B, and Tzi8 shared ∼99% sequence similarity between them, and each displayed ∼95% similarity to inbred line B73. As illustrated, despite sharing high degrees of sequence similarity, distinct regions of polymorphisms span the entire length of the elements within these inbred lines. These included indels and small clusters of nucleotide changes in addition to single nucleotide changes. We also cloned and sequenced a single *Hel1-331* specific RT-PCR product amplified in both roots and shoots of HP301, OH7B, and Tzi8. We then performed a splice alignment of each resultant transcript with their cognate inbred *Helitron*. Each line produced an identical gene structure with four exons and three introns (Figure 2A). This predicted gene structure is identical to one, isoform IV, of the eight different isoforms encoded by *Hel1-331* in B73 (Barbaglia *et al.* 2012). Noteworthy, the cognate region of the long intron 3 of this transcript encompasses the entirety of alternative splicing events and harbors the alternative donor and acceptor sites of the other seven transcript isoforms produced in B73 by *Hel1-331* (Barbaglia *et al.* 2012). Figure 2A schematically displays the position of these alternative donor and acceptor sites used to generate the other transcript isoforms of *Hel1-331* in B73.

Because sequences near splice-site junctions play a critical role in splice-site selection, we compared the flanking 15 bp of the splice site junctions for *Hel1-331* transcript isoforms in B73 with the cognate region in inbred lines HP301, OH7B, and Tzi8. Figure 2B displays the multiple sequence alignments of the splice-site junctions of *Hel1-331* transcripts in B73 with their corresponding sequence in inbred lines HP301, OH7B, and Tzi8.

A comparison of the donor and acceptor sites for intron 3 of this *Hel1-331* transcript from HP301, OH7B, and Tzi8, with intron 3 of the B73 transcript isoform IV is displayed in subpanels of Figure 2B. There are three single-nucleotide polymorphisms) between B73 and the other inbred lines. At one of these sites, a nucleotide C was substituted in three lines for a donor site G at −1 position in B73. Similarly, C replaced a donor site T at the +9 position in B73 and G replaced an acceptor site C at the −14 position in other inbred lines. The alignment of various B73-specific splice site junctions with other inbred lines are displayed in panels ii−vii of Figure 2B. For example, the donor site at the B73 alternative splicing event ii is the same as the donor for alternative splicing event i. However, A replaced an acceptor site T at the +5 position located within exon 4 in other inbred lines. The donor site for alternative splicing event iii displays no polymorphism between the inbred lines. However, the substitution of A to G at the −2 position alters the acceptor site dinucleotide from AG to GG in OH7B and Tzi8. Similarly, the donor site of alternative splicing event iv exhibits no changes, whereas the T in inbred B73 at position −15 of the acceptor site is replaced with A in other inbred lines. In addition, T in inbred HP301 replaces C, whereas the C remains unchanged in inbreds OH7B and Tzi8 at the −3 position of the acceptor site for inbred B73. The flanking sequences of splice sites used for alternative splicing event v were identical among the four inbred lines, except that C at +15 position of the acceptor site in B73 is replaced with T in other inbred lines. Similar to before, the splice sites of alternative splicing event vi are identical among four inbreds, except for an insertion of a G nucleotide at −12 position in other inbred lines and a T for an acceptor site C at +15 position in B73 in other three inbred lines. The flanking sequence of the donor site for alternatively spliced event vii remains identical among the different inbred lines, although it creates an acceptor site in exon 4, 95 bp downstream to the 3′ splice site of intron 3. The comparison of the flanking exon 4 sequences of the other inbred lines to the acceptor site identified a single polymorphism. Here, a T replaced an acceptor site C at −9 position in B73 in inbred line OH7B.

### Various alternatively spliced transcript isoforms of *Helitron Hel1-332* display inbred specific expression

**Figure 3:**
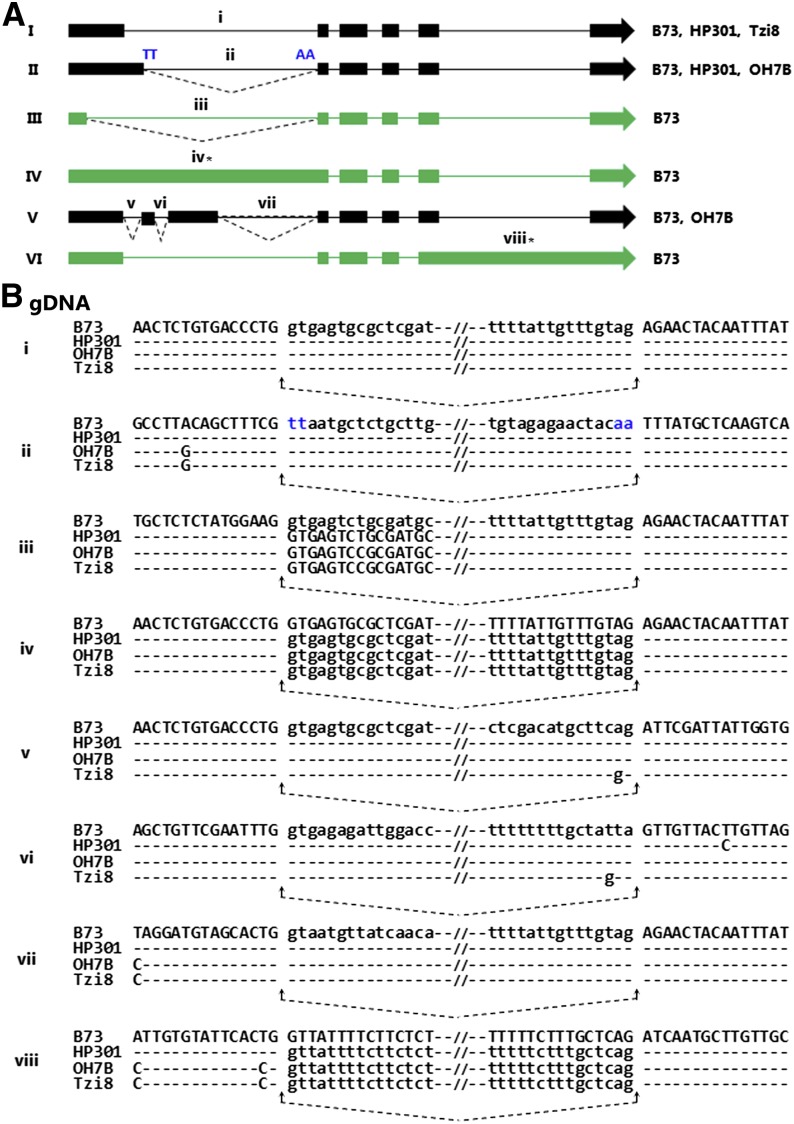
Alternatively spliced transcript isoforms of *Helitron Hel1-332* display inbred-specific expression. (A) Gene structure of the six transcript isoforms I−VI of *Hel1-332* in inbred line B73 (Barbaglia *et al.* 2012). The transcript isoforms, specific to B73 and not detected in other inbred lines, are highlighted in green. The noncanonical splice sites are shown in blue, and asterisks mark the retained introns. (B) Junction sequence alignment between the inbred lines of the splice sites marked by the lower case Roman numerals in (A).

In the same manner, to decipher the molecular basis of RT-PCR differences observed between these maize inbred lines, we cloned and sequenced a ∼3.9-kb fragment of *Helitron Hel1-332* PCR amplified from the genomic DNA isolated from inbred lines HP301, OH7B and Tzi8. Figure S2 displays the multiple alignment of the *Hel1-332* sequence from inbred lines B73, HP301, OH7B, and Tzi8. As shown, a high degree of similarity is shared between the *Hel1-332* sequences of the four inbred lines even though regions of polymorphism, including indels, span the entire length of the element. The sequence of inbred line HP301 displayed highest similarity of >99% with B73, whereas OH7B shared 97% and Tzi8 had 94% similarity with B73. The alternative splicing produces six isoforms of *Hel1-332* transcript in inbred line B73 (Barbaglia *et al.* 2012). In addition, we sequenced the two products amplified from the inbred lines HP301 and OH7B and a single product from inbred Tzi8. We performed splice alignment of the resultant products with their corresponding *Helitron* in different inbred lines to determine the gene structure and position of the intron/exon junctions. The gene structure of a single product of *Hel1-332* amplified from inbred Tzi8 is identical to transcript I of inbred B73 (Figure 3). Similarly, the two products amplified from HP301 displayed identical gene structure to transcripts I and II of inbred B73, whereas the two transcripts of OH7B were identical to transcripts II and V of B73. The transcripts III, IV, and VI were unique to B73 and were absent in other inbred lines. The majority of splicing events that give birth to various transcript isoforms are concentrated in the 5′ end of the *Helitron*, spanning exon 1 and intron 1 of transcript I (Figure 3). The exception is the retention of an unspliced intron 5 of transcript I in transcript VI spanning the 3′ end of the *Helitron*. The alternative splicing events that use donor or acceptor sites different from transcript I are displayed in Figure 2, A and B. As shown, the use of a noncanonical donor site within intron 1 and an acceptor site of exon 2 produces transcript II of B73. The flanking A at −10 position of the donor site in B73 was replaced with G in OH7B and Tzi8. Similarly, an alternative donor site within exon 1, an acceptor site of exon 2, and retention of an entire intron 1 give rise to B73 transcripts III and IV, respectively. As displayed in Figure 2B, no polymorphism was detected flanking the splice-site junction between the inbred lines of these splicing events. The use of several donor and acceptor sites creates two exons within intron 1 of B73 transcript V. There exists no polymorphism between the inbred lines flanking the splice junction of the first intron of this transcript. However, a flanking A at −2 position of the acceptor site of the second intron region in B73 was replaced by G in inbred Tzi8. In transcript vi, a G in inbred Tzi8 replaced T at −3 position of the acceptor site of the third intron region in B73. Similarly, T at +9 position of the acceptor site was replaced by C in inbred HP301. The retention of a complete intron 5 of B73 transcript isoform I produced transcript isoform VI. In transcript VI, the A at −15 position and T at −2 position in inbred B73 were both replaced by C in OH7B and Tzi8. The other splicing events common to all the six transcript isoforms of B73 did not display any junction site flanking polymorphism between the inbred lines.

### Alternative selections of splice sites create transcript isoform diversity of *Hel1-333* between maize inbred lines

**Figure 4:**
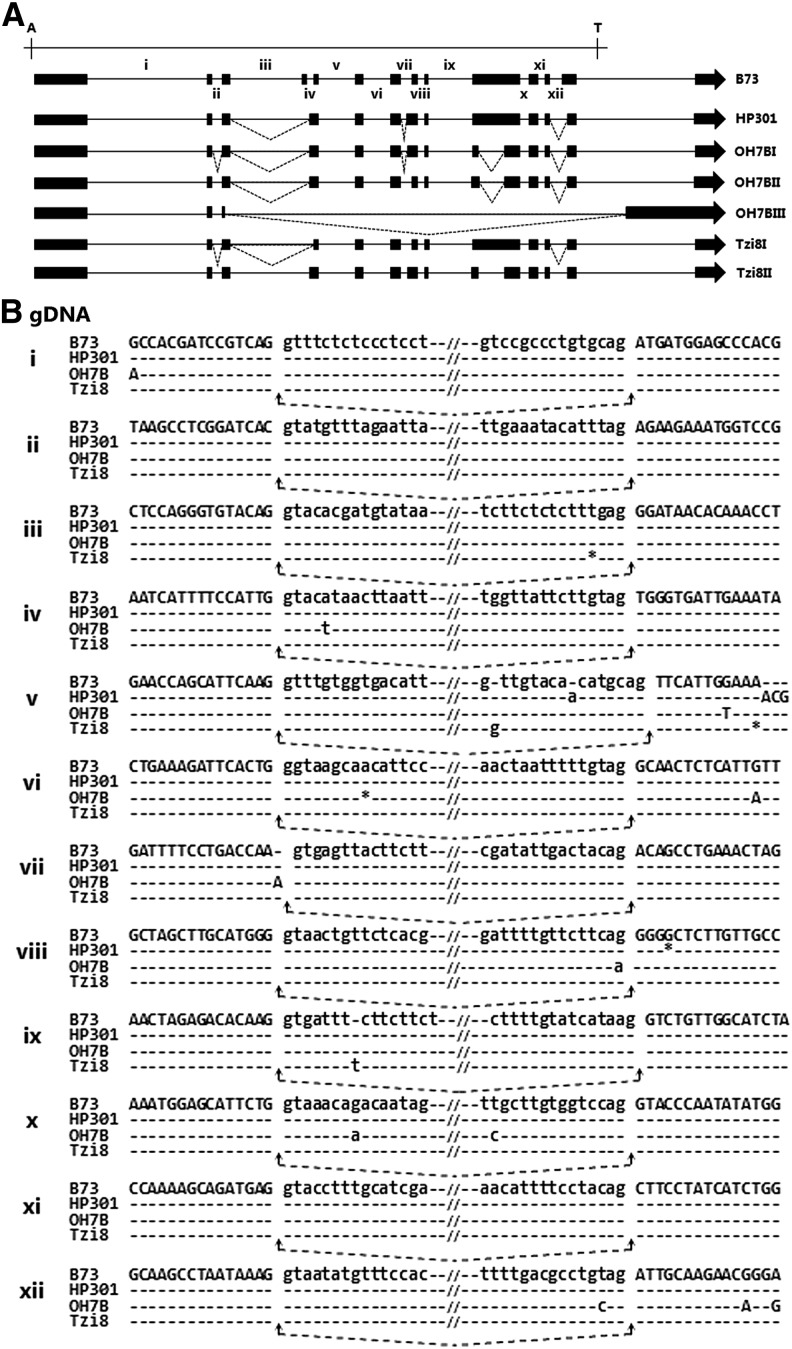
Alternative selections of splice sites create transcript isoform diversity of *Hel1-333* between maize inbred lines. (A) Schematic representation of the *Hel1-333* transcripts encoded by various inbred lines shown on the right. (B) Splice junction alignment between the inbred lines of the alternative splicing events marked by Roman numerals in (A).

The *Helitron Hel1-333* in maize inbred B73 encodes seven transcript isoforms via alternative splicing and read-through transcription of a flanking exon spliced to the captured gene transcript (Barbaglia *et al.* 2012). We amplified *Helitron Hel1-333* from inbred lines HP301, OH7B, and Tzi8 by using two pairs of overlapping primers. These primers are complementary to exon 1 and exon 13, and exon 13 and 14, respectively, of inbred B73 *Hel1-333* sequence. The resultant products of 6.7 kb and 1.9 kb in length were cloned and sequenced. As shown in Figure S3, *Hel1-333* sequence from the three inbred lines share a high degree of similarity with inbred B73 *Hel1-333*. For example, HP301 and OH7B each display >97% sequence similarity to B73, whereas Tzi8 displays >99% similarity. However, changes like indels and single-base changes interspersed throughout the length of the *Hel1-333* were detected between the inbred lines. Using primers spanning exon 1 and the flanking exon, we performed 14 RT-PCR on total RNA extracted from roots and shoots of different inbred lines. The resultant products were cloned, sequenced, and spliced aligned to their cognate *Hel1-333* inbred line. Inbred HP301 produces a single *Hel1-333*−specific transcript, whereas OH7B and Tzi8 each produce three and two transcripts, respectively (Figure 4). Intriguingly, the splicing profile of each *Hel1-333* transcript amplified from inbreds HP301, OH7B, and Tzi8 was distinct and did not display identity to any of the *Hel1-333* transcript isoforms of B73 (Barbaglia *et al.* 2012). To elucidate the molecular basis of splicing differences, we compared the alternative splicing profile of *Hel1-333* transcript isoforms of HP301, OH7B, and Tzi8 with B73 *Hel1-333* isoform I as a reference transcript.

As displayed in Figure 4, a single product amplified from HP301 revealed three regions of alternative splice-site usage compared with inbred B73 *Hel1-333* transcript I. The use of an alternative acceptor site within intron 4 and donor site of exon 3 results in the skipping of exon 4 and increases the length of exon 5 by 44 bp. The comparison of the splice-site junctions of intron 3 and intron 4, and the alternative acceptor site region within intron 4, displayed no polymorphism between inbred B73 and HP301, regardless of the loss of exon 4 (Figure 4). However, we note an insertion of A at the −8 position of the acceptor site of downstream intron 5 in HP301, and an insertion of 9 bp at position +12. In the same manner, the use of an alternative acceptor site within intron 7 and the donor site of exon 7 increases the length of exon 8 by 62 bp. Despite this difference, the splice junctions of intron 7 exhibit no polymorphism between B73 and HP301. However, the acceptor splice site of downstream intron 8 displays a deletion of G at position +4. Also, an alternative use of an acceptor site within exon 13 in conjunction with the donor site of intron 12 increases the length of intron 12 by 61 bp. The splice junctions of intron 12 and the alternative acceptor site within intron 12 are identical in B73 and HP301.

In the comparison between B73 and inbred OH7B, transcript I from OH7B displayed five regions of alternative splicing events. For example, use of an alternative acceptor site within exon 3 in combination with the donor site of intron 2 decreases the length of exon 3 by 5 bp. The comparison of splice junction sequence of intron 2 and flanking intron 1 and 3 between B73 and OH7B displays no polymorphism except for G at −15 position of the donor splice site of intron 1 in B73 is replaced by A in OH7B. Similar to the HP301 transcript, an alternative acceptor site within intron 4 results in the skipping of exon 4, and alters the length of exon 5 by 44 bp. Except for a replacement of A at +5 position of the donor splice site of intron 4 in B73 with T in OH7B, the splice junctions of introns 3 and 4, and the alternative acceptor site remain identical between these two inbred lines. In addition, an alternative acceptor site within intron 7 increases the length of exon 8 by 44bp. We note the donor splice site of intron 7 has an insertion of A at +1 position in OH7B compared with B73. Also, an A in OH7B replaces a G at −1 position of the acceptor site of intron 8 in B73. In addition, the use of an alternative donor and an alternative acceptor site creates an intron of 339 bp within exon 10. The splice junctions of the upstream intron display no polymorphism; however, an A in OH7B replaces G at position +8 of the donor splice site of downstream intron 10 in B73, and C in OH7B replaces T at position −14 of the acceptor splice site of intron 10 in B73. An acceptor site within exon 13, in combination with donor site of intron 12, reduces the length of exon 13 by 61bp. The T, G, and A at positions −3, +12, and +15 of the acceptor splice site of intron 12 in B73 is replaced by C, A, and G, respectively, in OH7B. The splicing profile of OH7B transcript II is similar to OH7B transcript I with three exceptions. Unlike OH7B transcript I, the alternative acceptor splice sites within exon 3 and intron 7 are not evoked in transcript II. Also, the use of an alternative donor and an alternative acceptor site creates an intron of 316 bp within exon 10. In OH7B transcript III, the use of alternative noncanonical splice sites GC-TG within exon 3 and intron 13 leads to the skipping of exon 2 to exon 13. The splice junctions of this non-canonical intron display no polymorphic sequences between B73 and OH7B.

The Tzi8 transcript I displays three distinct regions of alternative splice-site usage compared with B73 transcript I. Much like OH7B transcript I, an alternative acceptor site of intron 2 reduces the length of exon 3 by 5bp. The splice junctions of intron 2 remain identical in inbred lines B73 and Tzi8. Also, the employment of the donor site of intron 3, in conjunction with acceptor site of intron 4, results in skipping of exon 4. Except for a deletion of T at −4 position of the acceptor splice site of intron 3 in Tzi8, the splice junctions of flanking introns 2 and 4 remain identical between B73 and Tzi8. Furthermore, similar to HP301 transcript I, an alternative usage of an acceptor site within exon 13, in conjunction with the donor site of intron 12, increased the length of intron 12 by 61bp. The splice junctions of intron 12, and the region flanking the alternative acceptor site with exon 13, display no polymorphism between B73 and Tzi8. Similarly, splicing pattern of Tzi8 transcript II is identical to HP301 transcript I, except usage of an alternative donor and an alternative acceptor site within exon 10 creates an intron of 339bp. We note the donor site of intron 9 displays an insertion of T at +9.

## Discussion

We previously reported that transcript diversity of *Helitron*-captured genes is dramatically augmented by alternative splicing (Barbaglia *et al.* 2012). These potentially provide a potent diversity pool for the evolution of new genes via natural selection. Here, we report the expression analysis of selected *Helitrons* and demonstrate that differential usage of splice sites during pre-mRNA processing drastically alters the splicing profile and transcript isoform diversity between various maize inbred lines. We compared the sequences flanking the splice-site junctions between the inbred lines to investigate the *cis*-acting elements potentially underlying splicing differences. The vast majority of splicing aberration reported in both plants and animals have occurred from mutations within the proposed consensus region around the splice-site junction (Baralle and Baralle 2005; Krawczak *et al.* 2007; Schuler 2008). For example, a G to C transversion and a G to A transition at the +1 position of the donor splice site interferes with splicing and causes skipping of the downstream exon in both tomato and Arabidopsis, respectively (Jacobsen *et al.* 1996; Gorguet *et al.* 2009). Similarly, a base substitution at the −1 or −2 position of the donor splice site has also been reported to cause skipping of the downstream exon (Jack *et al.* 1992; McNellis *et al.* 1994; Sablowski and Meyerowitz 1998; Lal *et al.* 1999c; Schuler 2008), and the retention of unspliced intron or activation of a lariat-exon intermediate (Orozco *et al.* 1993; Liu and Filipowicz 1996; Lal *et al.* 1999c; Choe *et al.* 2000; Yuan *et al.* 2009). Comparisons of splice junction sites of *Helitron Hel1-331* in our study revealed several base substitutions between B73 and other inbred lines, including a G to C transversion at the +1 position of the donor splice site of intron 3 in a single *Hel1-331* transcript. This substitution alters the donor site consensus from AG/GT to AC/GT in inbreds HP301, OH7B, and Tzi8. Because the splice sites of all the seven B73 *Hel1-331* transcript isoforms reside in this intron 3 region, the most plausible explanation for the loss of alternative splicing in other inbred lines is this +1 position transversion substitution interfering with the recognition of their downstream splice sites. In this particular context, the various splice junction polymorphisms among the inbred lines detected at positions other than +1 of the donor splice site within intron 3, however, seem unlikely to play a role in splice-site selection due to the following observation. We have shown that a single-base substitution alters the acceptor site of B73 transcript isoform III from invariant terminal dinucleotide AG to GG in OH7B and Tzi8. Mutations that alter the acceptor site dinucleotides generally result in the complete abolishment of the splice site (Schuler 2008), and as expected, the transcript homologous to B73 transcript isoform III does not occur in OH7B and Tzi8. However, the absence of the B73 transcript isoform III in inbred HP301, which does not bear the homologous splice site alteration, indicates this mutation does not impact splice site selection in other inbred lines and lends support to the +1 transversion substitution as the causative polymorphism.

Despite significant differences in the number of *Hel1-332* transcript isoforms between the inbred lines, each transcript isoform of *Hel1-332* detected in inbreds HP301, OH7B, and Tzi8 was identical to one of the six transcript isoforms of *Hel1-332* reported in B73 (Barbaglia *et al.* 2012). The inspection of the polymorphism flanking the splice sites between the inbred lines does not provide clear clues to their impact on the selection of splice sites. It is quite plausible that variance in alternative splicing may be caused by polymorphisms distantly located from the splice sites. The recognition of the splice sites appears to be dependent on gene context. Intragenic mutations influencing the selection of splice sites from a distance have been reported in both plants and animals (McNellis *et al.* 1994; Marillonnet and Wessler 1997; Lal *et al.* 1999a). Intriguingly, each transcript isoform of *Hel1-333* was unique in each inbred and displayed different combinations of splice site usage. Similar to *Hel1-332*, inspection of the splice junction failed to reveal significant polymorphisms potentially underlying the observed splicing differences. Whether read-through transcription and capture of the flanking exon have evoked employment of cryptic splice sites by various *Hel1-333* transcript isoforms needs further investigation.

Unlike vertebrates, the mechanism of intron recognition and spliceosomal assembly is poorly understood in plants, mainly because of the lack of *in vitro* splicing assays. Despite sharing basic structural similarity, plant introns are not efficiently spliced in vertebrates and *vice versa* (Barta *et al.* 1986; Van Santen and Spritz 1987; Pautot *et al.* 1989; Barbazuk *et al.* 2008). Also, the presence of plant-specific splicing factors indicates intrinsic differences in splicing machinery between plants and animals (Barta *et al.* 2008; Rauch *et al.* 2014). Like vertebrates, the splicing differences of *Helitrons* among different inbred lines point to a complex array of signals that define the mechanism of splice site recognition in plants. For example, growing evidence in vertebrates points to short 5-to-10 nucleotide conserved elements located in both the introns and exons that affect splicing. Called splicing-responsive elements, they interact with various RNA-binding proteins and may suppress or enhance the use of splice sites (Chen and Manley 2009; Reddy *et al.* 2013). The mechanisms that regulate the use of splice sites remain uninvestigated in plants. The polymorphisms and mutations spanning the unidentified splicing-responsive elements may underlie the splicing differences of *Helitrons* between the inbred lines. In addition, the polymorphism may differentially impact the secondary structure of the *Helitron*-transcribed genes between the inbred lines. Mutations that alter the secondary structure of RNA are well documented in vertebrates (Warf and Berglund. 2010; Wan *et al.* 2011) and also have been shown to affect splice-site selection in plants (Goodall and Filipowicz 1991), perhaps by disrupting the site for RNA binding proteins.

It is well documented that pre-mRNA processing in vertebrates does not occur independently of other processes. Rather it is linked to transcription where various splicing factors participate in splicing before termination of transcription (Moore and Proudfoot 2009; Perales and Bentley 2009; Luco and Misteli 2011). These studies demonstrate a strong link between the pattern of alternative splicing and elongation rate of RNAPII (Kornblihtt *et al.* 2004; Luco and Misteli 2011; Luco *et al.* 2011). Because the elongation rate of RNA polymerase II (RNAPII) is controlled by chromatin structure, one explanation of the alternative splicing observed here is that *Helitron*-captured sequences of different genes have altered chromatin structure compared to their wild-type progenitors. This may alter the elongation rate of RNAPII transcribing *Helitron*-captured genes. The change in rate of RNAPII may enhance/alter access to the splicing elements recognized by the spliceosome, or it may alter the recruitment profile of splicing factors. Altogether, we envisage this could generate spurious splicing events leading to the appearance of multiple spliced transcripts that we have shown to exist in *Helitron*-captured genes.

Whether the polymorphisms between the *Helitrons* among the various inbred lines occur randomly or under the selection to promote or suppress particular transcript isoforms remains to be determined. Clearly they may have important implications for the process of gene evolution. Perhaps the differentially spliced transcript isoforms of *Helitrons* provide adaptive advantages to plants grown in environmentally diverse regions of the world.

## 

## Supplementary Material

Supporting Information

## References

[bib1] Baralle D. Baralle M., 2005 Splicing in action: assessing disease causing sequence changes. J. Med. Genet. 42: 737–748. 16199547 10.1136/jmg.2004.029538 PMC1735933

[bib2] Barbaglia A. M. Klusman K. M. Higgins J. Shaw J. R. Hannah L. C., 2012 Gene capture by *Helitron* transposons reshuffles the transcriptome of maize. Genetics 190: 965–975. 22174072 10.1534/genetics.111.136176 PMC3296258

[bib3] Barbazuk W. B. Fu Y. McGinnis K. M., 2008 Genome-wide analyses of alternative splicing in plants: opportunities and challenges. Genome Res. 18: 1381–1392. 18669480 10.1101/gr.053678.106

[bib4] Barta A. Sommergruber K. Thompson D. Hartmuth K. Matzke M. A., 1986 The expression of a nopaline synthase - human growth hormone chimaeric gene in transformed tobacco and sunflower callus tissue. Plant Mol. Biol. 6: 347–357. 24307385 10.1007/BF00034942

[bib5] Barta A. Kalyna M. Lorkovic Z. J., 2008 Plant SR proteins and their functions. Curr. Top. Microbiol. Immunol. 326: 83–102. 18630748 10.1007/978-3-540-76776-3_5

[bib6] Brunner S. Pea G. Rafalski A., 2005 Origins, genetic organization and transcription of a family of non-autonomous *Helitron* elements in maize. Plant J. 43: 799–810. 16146520 10.1111/j.1365-313X.2005.02497.x

[bib7] Capriglione T. De Paolo S. Cocca E., 2014 Helinoto, a Helitron2 transposon from the icefish *Chionodraco hamatus*, contains a region with three deubiquitinase-like domains that exhibit transcriptional activity. Comp. Biochem. Physiol. Part D Genomics Proteomics 11: 49–58. 25178533 10.1016/j.cbd.2014.07.004

[bib8] Chen M. Manley J. L., 2009 Mechanisms of alternative splicing regulation: insights from molecular and genomics approaches. Nat. Rev. Mol. Cell Biol. 10: 741–754. 19773805 10.1038/nrm2777 PMC2958924

[bib9] Choe S. Tanaka A. Noguchi T. Fujioka S. Takatsuto S., 2000 Lesions in the sterol delta reductase gene of Arabidopsis cause dwarfism due to a block in brassinosteroid biosynthesis. Plant J. 21: 431–443. 10758495 10.1046/j.1365-313x.2000.00693.x

[bib10] Corpet F., 1988 Multiple sequence alignment with hierarchical clustering. Nucleic Acids Res. 16: 10881–10890. 2849754 10.1093/nar/16.22.10881 PMC338945

[bib11] Du C. Fefelova N. Caronna J. He L. Dooner H. K., 2009 The polychromatic *Helitron* landscape of the maize genome. Proc. Natl. Acad. Sci. USA 106: 19916–19921. 19926866 10.1073/pnas.0904742106 PMC2785267

[bib12] Ellison C. E. Bachtrog D., 2013 Dosage compensation via transposable element mediated rewiring of a regulatory network. Science 342: 846–850. 24233721 10.1126/science.1239552 PMC4086361

[bib13] Feschotte C. Wessler S. R., 2001 Treasures in the attic: rolling circle transposons discovered in eukaryotic genomes. Proc. Natl. Acad. Sci. USA 98: 8923–8924. 11481459 10.1073/pnas.171326198 PMC55346

[bib14] Fu D. Wei L. Xiao M. Hayward A., 2013 New insights into *Helitron* transposable elements in the mesopolyploid species *Brassica rapa*. Gene 532: 236–245. 24055723 10.1016/j.gene.2013.09.033

[bib15] Goodall G. J. Filipowicz W., 1991 Different effects of intron nucleotide composition and secondary structure on pre-mRNA splicing in monocot and dicot plants. EMBO J. 10: 2635–2644. 1868837 10.1002/j.1460-2075.1991.tb07806.x PMC452964

[bib16] Gorguet B. Schipper D. van Lammeren A. Visser R. G. van Heusden A. W., 2009 ps-2, the gene responsible for functional sterility in tomato, due to non-dehiscent anthers, is the result of a mutation in a novel polygalacturonase gene. Theor. Appl. Genet. 118: 1199–1209. 19219598 10.1007/s00122-009-0974-9

[bib17] Gupta S. Gallavotti A. Stryker G. A. Schmidt R. J. Lal S. K., 2005 A novel class of *Helitron*-related transposable elements in maize contain portions of multiple pseudogenes. Plant Mol. Biol. 57: 115–127. 15821872 10.1007/s11103-004-6636-z

[bib18] Han M. J. Shen Y. H. Xu M. S. Liang H. Y. Zhang H. H., 2013 Identification and evolution of the silkworm *Helitrons* and their contribution to transcripts. DNA Res. 20: 471–484. 23771679 10.1093/dnares/dst024 PMC3789558

[bib19] Jack T. Brockman L. L. Meyerowitz E. M., 1992 The homeotic gene APETALA3 of *Arabidopsis thaliana* encodes a MADS box and is expressed in petals and stamens. Cell 68: 683–697. 1346756 10.1016/0092-8674(92)90144-2

[bib20] Jacobsen S. E. Binkowski K. A. Olszewski N. E., 1996 SPINDLY, a tetratricopeptide repeat protein involved in gibberellin signal transduction in Arabidopsis. Proc. Natl. Acad. Sci. USA 93: 9292–9296. 8799194 10.1073/pnas.93.17.9292 PMC38635

[bib21] Jameson N. Georgelis N. Fouladbash E. Martens S. Hannah L. C., 2008 *Helitron* mediated amplification of cytochrome P450 monooxygenase gene in maize. Plant Mol. Biol. 67: 295–304. 18327644 10.1007/s11103-008-9318-4

[bib22] Kapitonov V. V. Jurka J., 2001 Rolling-circle transposons in eukaryotes. Proc. Natl. Acad. Sci. USA 98: 8714–8719. 11447285 10.1073/pnas.151269298 PMC37501

[bib23] Kapitonov V. V. Jurka J., 2007 *Helitrons* on a roll: eukaryotic rolling-circle transposons. Trends Genet. 23: 521–529. 17850916 10.1016/j.tig.2007.08.004

[bib24] Krawczak M. Thomas N. S. Hundrieser B. Mort M. Wittig M., 2007 Single base-pair substitutions in exon-intron junctions of human genes: nature, distribution, and consequences for mRNA splicing. Hum. Mutat. 28: 150–158. 17001642 10.1002/humu.20400

[bib25] Kornblihtt A. R. de la Mata M. Fededa J. P. Munoz M. J. Nogues G., 2004 Multiple links between transcription and splicing. RNA 10: 1489–1498. 15383674 10.1261/rna.7100104 PMC1370635

[bib26] Lal S. K. Hannah L. C., 1999 a Maize transposable element Ds is differentially spliced from primary transcripts in endosperm and suspension cells. Biochem. Biophys. Res. Commun. 261: 798–801. 10441504 10.1006/bbrc.1999.1119

[bib27] Lal S. Choi J. H. Hannah L. C., 1999 b The AG dinucleotide terminating introns is important but not always required for pre-mRNA splicing in the maize endosperm. Plant Physiol. 120: 65–72. 10318684 10.1104/pp.120.1.65 PMC59270

[bib28] Lal S. Choi J. H. Shaw J. R. Hannah L. C., 1999 c A splice site mutant of maize activates cryptic splice sites, elicits intron inclusion and exon exclusion, and permits branch point elucidation. Plant Physiol. 121: 411–418. 10517832 10.1104/pp.121.2.411 PMC59403

[bib58] Lal S. K. Giroux M. J. Brendel V. Vallejos C. E. Hannah L. C., 2003 The maize genome contains a *Helitron* insertion. Plant Cell 15: 381–391. 12566579 10.1105/tpc.008375 PMC141208

[bib29] Lal S. Georgelis N. Hannah L. C., 2009 a *Helitrons*: Their impact on maize genome evolution and diversity, pp. 329–339 in Handbook of Maize, edited by Bennetzen J. Hake S. Springer, New York.

[bib30] Lal S. Oetjens M. Hannah L. C., 2009 b *Helitrons*: enigmatic abductors and mobilizers of host genome sequences. Plant Sci. 176: 181–186.

[bib31] Larkin M. A. Blackshields G. Brown N. P. Chenna R. McGettigan P. A., 2007 Clustal W and Clustal X version 2.0. Bioinformatics 23: 2947–2948. 17846036 10.1093/bioinformatics/btm404

[bib32] Li Y. Dooner H. K., 2009 Excision of *Helitron* transposons in maize. Genetics 182: 399–402. 19255366 10.1534/genetics.109.101527 PMC2674836

[bib33] Liu H. X. Filipowicz W., 1996 Mapping of branchpoint nucleotides in mutant pre-mRNAs expressed in plant cells. Plant J. 9: 381–389. 8919914 10.1046/j.1365-313x.1996.09030381.x

[bib34] Luco R. F. Misteli T., 2011 More than a splicing code: integrating the role of RNA, chromatin, and non-coding RNA in alternative splicing regulation. Curr. Opin. Genet. Dev. 21: 366–372. 21497503 10.1016/j.gde.2011.03.004 PMC6317717

[bib35] Luco R. F. Allo M. Schor I. E. Kornblihtt A. R. Misteli T., 2011 Epigenetics in alternative pre-mRNA splicing. Cell 144: 16–26. 21215366 10.1016/j.cell.2010.11.056 PMC3038581

[bib36] Marillonnet S. Wessler S. R., 1997 Retrotransposon insertion into the maize waxy gene results in tissue-specific RNA processing. Plant Cell 9: 967–978. 9212470 10.1105/tpc.9.6.967 PMC156971

[bib37] McNellis T. W. von Arnim A. G. Araki T. Komeda Y. Misera S., 1994 Genetic and molecular analysis of an allelic series of cop1 mutants suggests functional roles for the multiple protein domains. Plant Cell 6: 487–500. 8205001 10.1105/tpc.6.4.487 PMC160452

[bib38] Moore M. J. Proudfoot N. J., 2009 Pre-mRNA processing reaches back to transcription and ahead to translation. Cell 136: 688–700. 19239889 10.1016/j.cell.2009.02.001

[bib39] Morgante M. Brunner S. Pea G. Fengler K. Zuccolo A., 2005 Gene duplication and exon shuffling by *Helitron*-like transposons generate intraspecies diversity in maize. Nat. Genet. 37: 997–1002. 16056225 10.1038/ng1615

[bib40] Orozco B. M. McClung C. R. Werneke J. M. Ogren W. L., 1993 Molecular basis of the ribulose-1,5-bisphosphate carboxylase/oxygenase activase mutation in *Arabidopsis thaliana* is a guanine-to-adenine transition at the 5′-splice junction of intron 3. Plant Physiol. 102: 227–232. 8108496 10.1104/pp.102.1.227 PMC158767

[bib41] Pautot V. Brzezinski R. Tepfer M., 1989 Expression of a mouse metallothionein gene in transgenic plant tissues. Gene 77: 133–140. 2744484 10.1016/0378-1119(89)90367-3

[bib42] Perales R. Bentley D., 2009 “Cotranscriptionality”: the transcription elongation complex as a nexus for nuclear transactions. Mol. Cell 36: 178–191. 19854129 10.1016/j.molcel.2009.09.018 PMC2770090

[bib43] Pritham E. J. Feschotte C., 2007 Massive amplification of rolling-circle transposons in the lineage of the bat *Myotis lucifugus*. Proc. Natl. Acad. Sci. USA 104: 1895–1900. 17261799 10.1073/pnas.0609601104 PMC1794268

[bib44] Rauch H. B. Patrick T. L. Klusman K. M. Battistuzzi F. U. Mei W., 2014 Discovery and expression analysis of alternative splicing events conserved among plant SR proteins. Mol. Biol. Evol. 31: 605–613. 24356560 10.1093/molbev/mst238

[bib45] Reddy A. S. Marquez Y. Kalyna M. Barta A., 2013 Complexity of the alternative splicing landscape in plants. Plant Cell 25: 3657–3683. 24179125 10.1105/tpc.113.117523 PMC3877793

[bib46] Sablowski R. W. Meyerowitz E. M., 1998 Temperature-sensitive splicing in the floral homeotic mutant apetala3–1. Plant Cell 10: 1453–1463. 9724692 10.1105/tpc.10.9.1453 PMC144071

[bib47] Schuler M. A., 2008 Splice site requirements and switches in plants. Curr. Top. Microbiol. Immunol. 326: 39–59. 18630746 10.1007/978-3-540-76776-3_3

[bib48] Tavakoli N. Comanducci A. Dodd H. M. Lett M. C. Albiger B., 2000 IS1294, a DNA element that transposes by RC transposition. Plasmid 44: 66–84. 10873528 10.1006/plas.1999.1460

[bib49] Thomas J. Phillips C. D. Baker R. J. Pritham E. J., 2014 Rolling-circle transposons catalyze genomic innovation in a Mammalian lineage. Genome Biol. Evol. 6: 2595–2610. 25223768 10.1093/gbe/evu204 PMC4224331

[bib50] Usuka J. Brendel V., 2000 a Gene structure prediction by spliced alignment of genomic DNA with protein sequences: increased accuracy by differential splice site scoring. J. Mol. Biol. 297: 1075–1085. 10764574 10.1006/jmbi.2000.3641

[bib51] Usuka J. Zhu W. Brendel V., 2000 b Optimal spliced alignment of homologous cDNA to a genomic DNA template. Bioinformatics 16: 203–211. 10869013 10.1093/bioinformatics/16.3.203

[bib52] Van Santen V. L. Spritz R. A., 1987 Splicing of plant pre-mRNAs in animal systems and vice versa. Gene 56: 253–265. 3678838 10.1016/0378-1119(87)90142-9

[bib53] Wan Y. Kertesz M. Spitale R. C. Segal E. Chang H. Y., 2011 Understanding the transcriptome through RNA structure. Nat. Rev. Genet. 12: 641–655. 21850044 10.1038/nrg3049 PMC3858389

[bib54] Warf M. B. Berglund J. A., 2010 Role of RNA structure in regulating pre-mRNA splicing. Trends Biochem. Sci. 35: 169–178. 19959365 10.1016/j.tibs.2009.10.004 PMC2834840

[bib55] Xiong W. He L. Lai J. Dooner H. K. Du C., 2014 HelitronScanner uncovers a large overlooked cache of *Helitron* transposons in many plant genomes. Proc. Natl. Acad. Sci. USA 111: 10263–10268. 24982153 10.1073/pnas.1410068111 PMC4104883

[bib56] Yang L. Bennetzen J. L., 2009 Distribution, diversity, evolution, and survival of *Helitrons* in the maize genome. Proc. Natl. Acad. Sci. USA 106: 19922–19927. 19926865 10.1073/pnas.0908008106 PMC2785268

[bib57] Yuan Y. X. Wu J. Sun R. F. Zhang X. W. Xu D. H., 2009 A naturally occurring splicing site mutation in the *Brassica rapa* FLC1 gene is associated with variation in flowering time. J. Exp. Bot. 60: 1299–1308. 19190098 10.1093/jxb/erp010 PMC2657548

