# Supplementary material for: Differential pre-mRNA Splicing Alters the Transcript Diversity of Helitrons Between the Maize Inbred Lines
Source: G3 (Bethesda). 2015 Jun 12;5(8):1703–11. doi: 10.1534/g3.115.018630 (PMC4528327; doi:10.1534/g3.115.018630)
Supplement: Supporting Information [file supp_5_8_1703__index.html]

Differential pre-mRNA Splicing Alters the Transcript Diversity of Helitrons Between the Maize Inbred Lines — Supporting Information 

# Differential pre-mRNA Splicing Alters the Transcript Diversity of *Helitrons* Between the Maize Inbred Lines

## Supporting Information for Lynch *et al.*, 2015

**Files in this Data Supplement:**

- Supporting Information - Figures S1-S3 (PDF, 569 KB)
- Figure S1 - Sequence Alignment of *Helitron Hel1-331* between the lines of maize. (PDF, 266 KB)
- Figure S2 - Alignment of *Helitron Hel1-332* between the maize inbred lines. (PDF, 275 KB)
- Figure S3 - Alignment of *Helitron Hel1-333* sequence between the maize inbred lines. (PDF, 314 KB)
