# Supplementary material for: Differential pre-mRNA Splicing Alters the Transcript Diversity of Helitrons Between the Maize Inbred Lines
Source: G3 (Bethesda). 2015 Jun 12;5(8):1703–11. doi: 10.1534/g3.115.018630 (PMC4528327; doi:10.1534/g3.115.018630)
Supplement: Supporting Information [file supp_g3.115.018630_018630SI.pdf]

G3 · Genes | Genomes | Genetics

Differential pre-mRNA Splicing Alters the Transcript Diversity of *Helitrons* Between the Maize Inbred Lines

Brian T. Lynch[*], Tara L. Patrick[*], Jennifer J. Moreno[*], Amy E. Siebert[*], Katarina M. Klusman[*], Donya N. Shodja[*], L. Curtis Hannah[§], and Shailesh K. Lal[*1]

[*] Department of Biological Sciences, Oakland University, Rochester, MI 48309-4401

[§]Department of Horticultural Sciences and Program in Plant Molecular and Cellular Biology, University of Florida, Gainesville, FL 32611

[1]Corresponding Author:
Shailesh Lal, Oakland University 3200 N. Squirrel Rd. Room 347 Dodge Hall of Engineering Rochester, MI 48309
Phone: (248) 370-2875
Email: lal@oakland.edu

The sequences for *Hel1-331*, *Hel1-332*, *Hel1-333*, and their correlating transcripts are deposited under the GenBank accession numbers:

KF294013, KF294014, KF294015, KF294016, KF294017, KF294018, KF294019, KF294020, KF294021, KF294022, KF294023, KF294024, KF294025, KF294026, KF294027, KF294028, KF294029, KF294030, KF294031, KF294032, KF294033, KF294034, KF294035

DOI: 10.1534/g3.115.018630

```
B73             TTCTCAAAGGAATTCAGAATTTGGCCCTTCGTTTGAGCAAAATAAATGTGAGGTCATGAT
HP301           TTCTCAAAGGAATTCAGAATTTGGCCCTTCGTTTGAGCAAAATAAATGTGAGGTCATGAT
OH7B            TTCTCAAAGGAATTCAGAATTTGGCCCTTCGTTTGAGCAAAATAAATGTGAGGTCATGAT
Tzi8            TTCTCAAAGGAATTCAGAATTTGGCCCTTCGTTTGAGCAAAATAAATGTGAGGTCATGAT

B73             GATGGTCGGTCTTCCGGCCACTGGCAAGTCAACATGGGCAGAGAAATGGGTCAAGGAGCA
HP301           GATGGTCGGTCTTCCGACCTCTGGCAAGTCAACATGGGCAGAGAAATGGGTCAAGGAGCA
OH7B            GATGGTCGGTCTTCCGACCTCTGGCAAGTCAACATGGGCAGAGAAATGGGTCAAGGAGCA
Tzi8            GATGGTCGGTCTTCCGACCTCTGGCAAGTCAACATGGGCAGAGAAATGGGTCAAGGAGCA

B73             TCAGGAGAAACGTTATATCCTTCTCGGAACTAACCTTGTATTGGAGCAAATGAAGGTTGG
HP301           TCAGGAGAAACGTTATATCCTTCTCGGAACTAACCTTGTATTGGAGCAAATGAAGGTTGG
OH7B            TCAGGAGAAACGTTATATCCTTCTCGGAACTAACCTTGTATTGGAGCAAATGAAGGTTGG
Tzi8            TCAGGAGAAACGTTATATCCTTCTCGGAACTAACCTTGTATTGGAGCAAATGAAGGTTGG

B73             TTTCCTTCTGGTGGCAATTGTATTAGGGAGTTCCCTTAATCCTGCAACCTTT-TTTTCAA
HP301           TTTCCTTCTAGTGGCAATTGTATTAGGGAGTTCCCTTAATCCTGCAACCTTTTTTTTCAA
OH7B            TTTCCTTCTAGTGGCAATTGTATTAGGGAGTTCCCTTAATCCTGCAACCTTT-TTTTCAA
Tzi8            TTTCCTTCTAGTGGCAATTGTATTAGGGAGTTCCCTTAATCCTGCAACCTTT-TTTTCAA

B73             TATATAACTAATACCTTGATCATTGCAGGTGCCAGGATTATTGCGTAAGAATAACTATGG
HP301           TATATAACTAATACCTTGATCATTGCAGGTGCCAGGATTATTGCGTAAGAATAACTATGG
OH7B            TATATAACTAATACCTTGATCATTGCAGGTGCCAGGATTATTGCGTAAGAATAACTATGG
Tzi8            TATATAACTAATACCTTGATCATTGCAGGTGCCAGGATTATTGCGTAAGAATAACTATGG

B73             TGAGCGTTTTGAACGACTGATGGATTGTGCTACATGGATTTTCAACAAACTGCTGACTAG
HP301           TGAGCGTTTTGAACGACTGATGGATTGTGCTACATGGATTTTCAACAAACTGCTGACTAG
OH7B            TGAGCGTTTTGAACGACTGATGGATTGTGCTACATGGATTTTCAACAAACTGCTGACTAG
Tzi8            TGAGCGTTTTGAACGACTGATGGATTGTGCTACATGGATTTTCAACAAACTGCTGACTAG

B73             GGCTGCAAATACCCCTCGCAACTTCATTATCGATCAAACGAATGTTTACAAGAATGCTCG
HP301           GGCTGCAAATACCCCTCGCAACTTCATTATCGATCAAACGAATGTTTACAAGAATGCTCG
OH7B            GGCTGCAAATACCCCTCGCAACTTCATTATCGATCAAACGAATGTTTACAAGAATGCTCG
Tzi8            GGCTGCAAATACCCCTCGCAACTTCATTATCGATCAAACGAATGTTTACAAGAATGCTCG

B73             CATTCGCAAGTTGAGGCCATTTGCTAACTACCGCAAGGTAAGCTGTAAAAGAGAAAAAGG
HP301           CATTCGCAAGTTGAGGCCATTTGCTAACTACCGCAACGTAAGCTGCAAAAGAGAAAAAGG
OH7B            CATTCGCAAGTTGAGGCCATTTGCTAACTACCGCAACGTAAGCTGCAAAAGAGAAAAAGG
Tzi8            CATTCGCAAGTTGAGGCCATTTGCTAACTACCGCAACGTAAGCTGCAAAAGAGAAAAAGG
                                      ************** ******** ******
                                                    ↑ Donor i & ii
B73             AAATGATAGGTTTCCTGTATGGTAGCACTGGATTGGTTCATAAATACTCCCATATGCAAC
HP301           AAATGATAGGTTTCCTGTATGGTAGCACTGGATTGGTTCATAAATACTCCCATATGCAAC
OH7B            AAATGATAGGTTTCCTGTATGGTAGCACTGGATTGGTTCATAAATACTCCCATATGCAAC
Tzi8            AAATGATAGGTTTCCTGTATGGTAGCACTGGATTGGTTCATAAATACTCCCATATGCAAC
                                                               *************

B73             AGACTGATGTGGTCATGTTCCCAACTCCAAGTGAACTGAAATCCAGGGCAGCGAAGCGTT
HP301           AGACTGTTGTGGTCATGTTCCCAACTCCAAGTGAACTGAAATCCAGGGCAGCGAAGCTTT
OH7B            AGACTGTTGTGGTCATGTTCCCAACTCCAAGTGAACTGAAATCCAGGGCAGCGAAGCTTT
Tzi8            AGACTGTTGTGGTCATGTTCCCAACTCCAAGTGAACTGAAATCCAGGGCAGCGAAGCTTT
                ****** **********
                  ↑ Acceptor ii
```

```
B73      TCAATGAGATGGGAAAGGAAATACCAGCTGAAGCAGTTAACGAAATGACAGGTACCTGGA
HP301    TCAATGAGATGGGAAAGGAAATACCAGCTGAAGCAGTTAACGAAATGACAGGTACCTGGA
OH7B     TCAATGAGATGGGAAAGGAAATACCAGCTGAAGCAGTTAACGAAATGACAGGTACCTGGA
Tzi8     TCAATGAGATGGGAAAGGAAATACCAGCTGAAGCAGTTAACGAAATGACAGGTACCTGGA
                                             ************************
                                                            ⬆ Donor iii

B73      CATACACACATTATCATTTAACATGTTCGCTGTCTAGCTCCTTTTTTTCCCATTATCTAT
HP301    CATACACACATTATCATTTAACATGTTCGCTGCCTAGCTCCTTT--TTCCCATTATCTAT
OH7B     CATACACACATTATCATTTAACATGTTCGCTGCCTAGCTCCTTT---TCCCATTATCTAT
Tzi8     CATACACACATTATCATTTAACATGTTCGCTGCCTAGCTCCTTT---TCCCATTATCTAT
         ******

B73      ACATCCATTACTTACCAGTTACCTTAAATTATCTTTTTCAGCCAATTTTGTCCTACCACT
HP301    ACATCCATTACTTACCAGTTACCTTAAATTATCTTTTTCAGCCAATTTTGTCCTACCACT
OH7B     ACATCCATTACTTACCAGTTACCTTAAATTATCTTTTTCGGCCAATTTTGTCCTACCACT
Tzi8     ACATCCATTACTTACCAGTTACCTTAAATTATCTTTTTCGGCCAATTTTGTCCTACCACT
                                   ************* ***************
                                                ⬆ Acceptor iii

B73      CTCGAAGGATATGCCTCATTCGAAGGAGCCTTTTGATGAGGTATACTCAAATGTTGATAT
HP301    CTCGAAGGATATGCCTCATTCGAAGGAGCCTTTTGATGAGGTATACTCAAATGTTGATAT
OH7B     CTCGAAGGATATGCCTCATTCGAAGGAGCCTTTTGATGAGGTATACTCAAATGTTGATAT
Tzi8     CTCGAAGGATATGCCTCATTCGAAGGAGCCTTTTGATGAGGTATACTCAAATGTTGATAT
                                  ******************************
                                                ⬆ Donor iv

B73      TATTGTACATCTTCTGACTAGCGTCAAATGATTACTTAGCTCATTGAGCTTTAAAAACAT
HP301    TATTGTACATCTTCTGACTAGCGTCAAATGATTACTTAGCTCATTGAGCTTAAAAAACAT
OH7B     TATTGTACATCTTCTGACTAGCGTCAAATGATTACTTAGCTCATTGAGCTTAAAAAACAT
Tzi8     TATTGTACATCTTCTGACTAGCGTCAAATGATTACTTAGCTCATTGAGCTTAAAAAACAT
                                                             ********

B73      TTTCAGGTAATCTTCACAGAGCTTTCTAGGGATGAGGCTCAGAGAACCCTAGATGATATG
HP301    TTTTAGGTAATCTTCACAGAGCTTTCTAGGGATGAGGCTCAGAGAACCCTAGATGATATG
OH7B     TTTCAGGTAATCTTCACAGAGCTTTCTAGGGATGAGGCTCAGAGAACCCTAGATGATATG
Tzi8     TTTCAGGTAATCTTCACAGAGCTTTCTAGGGATGAGGCTCAGAGAACCCTAGATGATATG
         *** *****************
              ⬆ Acceptor iv

B73      CAGCGTGTGTTGCCAAGAAATGTGACCCCAAGCTATGGAAACTCTGGCAACCAAAAACAT
HP301    CAGCGTGTGTTGCCAAGAAATGTGACCCCAAGCTATGGAAACTCTGGCAACCAAAAACAT
OH7B     CAGCGTGTGTTGCCAAGAAATGTGACCCCAAGCTATGGAAACTCTGGCAACCAAAAACAT
Tzi8     CAGCGTGTGTTGCCAAGAAATGTGACCCCAAGCTATGGAAACTCTGGCAACCAAAAACAT
                                                      ***************

B73      GTAATGGTGCCTTTATTGTTATTTTTGAAACAGTGCCTTGTATGTTTTATGTCATCATGG
HP301    GTAATGGTGCCTTTATTGTTATTTTTGAAACAGTGCCTTGTATGTTTTATGTCATCATGG
OH7B     GTAATGGTGCCTTTATTGTTATTTTTGAAACAGTGCTTTGTATGTTTTATGTCATCATGG
Tzi8     GTAATGGTGCCTTTATTGTTATTTTTGAAACAGTGCCTTGTATGTTTTATGTCATCATGG
         ***************
        ⬆ Donor v

B73      ATCAGATTAGTGTAAATATGCAACGCTATACAACAATGTTCTGATGTTCTGGGACTGC--
HP301    ATCAGATTAGTGTAAATATGCAACGCTATACAACAATGTTCTGATGTTCTGGTGCTTACA
OH7B     ATCAGATTAGTGTAAATATGCAACGCTATACAACAATGTTCTGATGTTCTGGTACTTACA
Tzi8     ATCAGATTAGTGTAAATATGCAACGCTATACAACAATGTTCTGATGTTCTGGTACTTACA

B73      ---------------------------TGCTCCACTTGATCCTGAAGCCACTT-TGCAAA
HP301    GTATAGTTCAACATATGCTGGGACTGCTGCTCCACTTGATCCTGAAGCCACTTGTGCAAA
OH7B     GTATAGTTCAACATATGCTGGGACTGCTGCTCCACTTGATCCTGAAGCCACTTGTGCAAA
Tzi8     GTATAGTTCAACATATGCTGGGACTGCTGCTCCACTTGATCCTGAAGCCACTTGTGCAAA
                                                           *** ******
```

```
B73           TCAAGGTGCACAGTTTTCAAACATCTGGTGTTATTTTCCTATTTATTTAGTGAGACATTT
HP301         TCAAGGTGCACAGTTTTCAAACATCTGGTGTTATTTTCCTATTTATTTAGTGAGACATTT
OH7B          TCAAGGTGCACAGTTTTCAAACATCTGGTGTTATTTTCCTATTTATTTAGTGAGACATTT
Tzi8          TCAAGGTGCACAGTTTTCAAACATCTGGTGTTATTTTCCTATTTATTTAGTGAGACATTT
              ********************
                   ⬆ Donor vi

B73           GGAGTTCATTTTGTCTGAATTAAACAACAAGTTTCAGTTGAGGTTAACTGAAATACGTGA
HP301         GGAGTTCATTTTGTCTGAATTAAATAACAAGTTTCAGTTGAGGTTAACTGAAATATGTGA
OH7B          GGAGTTCATTTTGTCTGAATTAAATAACAAGTTTCAGTTGAGGTTAACTGAAATATGTGA
Tzi8          GGAGTTCATTTTGTCTGAATTAAATAACAAGTTTCAGTTGAGGTTAACTGAAATATGTGA

B73           TTTTGTATAAACTGAACCAACGTAAACCAATTTTATTAGGCTACTTAGTAGCAGGGATAT
HP301         TTTTGTATAAACTGAACCAACGTAAACCAATTTTATTAGGCTACTTAGTAGCAGGGATAT
OH7B          TTTTGTATAAACTGAACCAATGTAAACCAATTTTATTAGGCTACTTAGTAGCAGGGATAT
Tzi8          TTTTGTATAAACTGAACCAATGTAAACCAATTTTATTAGGCTACTTAGTAGCAGGGATAT

B73           AAATTAATATAAAGAATGGAAGTTGTTAACTTACATAAACTACTGTGTTGAATTCATGTT
HP301         AAATTAATATAAAGAATGGAAGTTGTTAACTTGCATAAACTACTGTGTTGAAATCATGTT
OH7B          AAATTAATATAAAGAATGGAAGTTGTTAACTTACATAAACTACTGTGTTGAATTCATGTT
Tzi8          AAATTAATATAAAGAATGGAAGTTGTTAACTTACATAAACTACTGTGTTGAATTCATGTT

B73           CATTTTAGCCTTGTCTGCACCTGTTAACTATCCATTTGTCTATACAACTAAGTGCAGTTC
HP301         CATTTTAGCCTTGTCTGCACCTGTTAACTACCCATTTGTCTATACAACTAAGTGCAGTTC
OH7B          CATTTTAGCCTTGTCTGCACCTGTTAACTACCCATTTGTCTATACAACTAAGTGCAGTTC
Tzi8          CATTTTAGCCTTGTCTGCACCTGTTAACTACCCATTTGTCTATACAACTAAGTGCAGTTC

B73           ATAGTCCAAACTAAGGTATCACCATTTTGTCTTAATTATTGCATACAAAACTAATGAAAC
HP301         ATAGTCCGAACTAATGTATCACCATTTTGTCTTAATTATTGCATACAAAACTAATGAAAC
OH7B          ATAGTCCGAACTAATGTATCACCATTTTGTCTTAATTATTGCATACAAAACTAATGAAAC
Tzi8          ATAGTCCGAACTAATGTATCACCATTTTGTCTTAATTATTGCATACAAAACTAATGAAAC

B73           ATGATTATGTTTCTGCATTTTAACGAGTGAGTAGTCCCATAAAATATCAAGAACGACATC
HP301         ATGATTATATTTCTGCATTTTAACGAGCGAGTAGTCCCATAAAATATCAAGAACGACATC
OH7B          ATGATTATGTTTCTGCATTTTAACGAGTGAGTAGTCCCATAAAATATCAAGAACGACATC
Tzi8          ATGATTATGTTTCTGCATTTTAACGAGTGAGTAGTCCCATAAAATATCAAGAACGACATC

B73           CAAAACTAATCCG-TTTTTTTCACGAGGTTTGCTGACATAAGTGGCTTTTGTTTTTGTAG
HP301         CAAAACTAATCCCGTTTTTTTTCACGTGGTTTGCTGACATAAGTGGCTTTTGTTTTTGTAG
OH7B          CAAAACTAAC-CCGTTTTTTTTCACGTGGTTTGCTGACATAAGTGGCTTTTGTTTTTGTAG
Tzi8          CAAAACTAAC-CCGTTTTTTTTCACGTGGTTTGCTGACATAAGTGGCTTTTGTTTTTGTAG

B73           TAGCCAGTTGTCCTTTCTGAACCCTCTCATGCATTGAACCCTCTCATCTGTTGTTATGTT
HP301         TAGCCAGTTGTCCTTTCTGAACCCTCTCATGCATGGAACCCTCTCATCTGTTGTTATGTT
OH7B          TAGCCAGTTGTCCTTTCTGAACCCTCTCATGCATGGAACCCTCTCATCTGTTGTTATGTT
Tzi8          TAGCCAGTTGTCCTTTCTGAACCCTCTCATGCATGGAACCCTCTCATCTGTTGTTATGTT
                                                                   *****

B73           TGTACAACAGTGTTGTACGAGTGCCCGCGACAGGAGCTTGTACGCCAGTGTGTTTTCTTC
HP301         TGTACAACAGTGTTGTACGAGTGCCTGCGACAGGAGCTTGTACGCTAGTGTGTTTTCTTC
OH7B          TGTACAACAGTGTTGTACGAGTGCCTGCGACAGGAGCTTGTACGCTAGTGTGTTTTCTTC
Tzi8          TGTACAACAGTGTTGTACGAGTGCCTGCGACAGGAGCTTGTACGCTAGTGTGTTTTCTTC
              *************************
                        ⬆ Acceptor v & vi
```

```
B73        CGGCTGATTGTTTTGCTCTGTGAAGGTTTGAATTCTCGAACATAGTATAGATCAATCATT
HP301      CGGCTGATTGTTTTGCTCTGTGAAGGTTTGAATTCTCGAACATAGTATAGATCAATCATT
OH7B       CGGCTGATTGTTTTGCTCTGTGAAGGTTTGAATTCTCGAACATAGTATAGATCAATCATT
Tzi8       CGGCTGATTGTTTTGCTCTGTGAAGGTTTGAATTCTCGAACATAGTATAGATCAATCATT
                    *******************************
                                ⬆ Donor vii

B73        CAATTAGATGCATTATGTTGGCATCAATATCCAGTGATCTGAGTAGCTTGTACAAAACAT
HP301      CAATTAGATGCATTAGGTTGGCATTAATATCCAGTGATCTGAGTAGCTTGTACAAAACAT
OH7B       CAATTAGATGCATTAGGTTGGCATTAATATCCAGTGATCTGAGTAGCTTGTACAAAACAT
Tzi8       CAATTAGATGCATTAGGTTGGCATTAATATCCAGTGATCTGAGTAGCTTGTACAAAACAT

B73        TATGCCAACCTAATGATAAAACTGTTTTGAATGATTGATACTCAATATCCAGTGAGCTGC
HP301      TATGCCAACCTAATGATAAAACTGTTTTGAATGATTGATACTCAATATCCAGTGAGCTGC
OH7B       TATGCCAACCTAATGATAAAACTGTTTTGAATGATTGATACTCAATATCCAGTGAGCTGC
Tzi8       TATGCCAACCTAATGATAAAACTGTTTTGAATGATTGATACTCAATATCCAGTGAGCTGC

B73        TATAACACTTATACGCATTTTCACTCTTTTGATCCAAGAATAAAATTGCGTATATATTCT
HP301      TATAACATTTATACGCATTTTCACTCTTTTGATCCAAGAATAAAATTGCGTATATATTCT
OH7B       TATAACATTTATACGCATTTTCACTCTTTTGATCCAAGAATAAAATTGCGTATATATTGT
Tzi8       TATAACATTTATACGCATTTTCACTCTTTTGATCCAAGAATAAAATTGCGTATATATTGT
                                                                    * *

B73        GGATCAAAAGAGTGAAACTGTGTATAAGTGCTATAACAGCTTGTGTAGTTCTCATGAGGG
HP301      GGATCAAAAGAGTGAAACTGTGTATAAGTGCTATAATAGCTTGTGTAGTTCTCATGAGGG
OH7B       GGATCAAAAGAGTGAAACTGTGTATAAGTGCTATAATAGCTTGTGTAGTTCTCATGAGGG
Tzi8       GGATCAAAAGAGTGAAACTGTGTATAAGTGCTATAATAGCTTGTGTAGTTCTCATGAGGG
           ***************************
                       ⬆ Acceptor i

B73        AATTTGACCCGGGGAACATTGTCTTTTATATTTTTGTTCGGCTGCAGTCGTTGGTGCCAC
HP301      AATTTGACCCGGGGAACATTGTCTTTTATATTTTTGTTCGGCTGCAGTCGTTGGTGCCAC
OH7B       AATTTGACCCGGGGAACATTGTCTTTTATATTTTTGTTTGGCTGCAGTCGTTGGTGCCAC
Tzi8       AATTTGACCCGGGGAACATTGTCTTTTATATTTTTGTTCGGCTGCAGTCGTTGGTGCCAC
                                           ****** *********************
                                                          ⬆ Acceptor vii

B73        CATCACACTGCTAGCGTGCAGAAACTCCTCTGTTGATTTAATCCTATCTCCATCAGTGAT
HP301      CATCACACTGCTAGCGTGCAGAAACTCCTCTGTTGATTTAATCCTATCTCCATCAGTGAT
OH7B       CATCACACTGCTAGCGTGCAGAAACTCCTCTGTTGATTTAATCCTATCTCCATCAGTGAT
Tzi8       CATCACACTGCTAGCGTGCAGAAACTCCTCTGTTGATTTAATCCTATCTCCATCAGTGAT
           **

B73        ATAAAGGTACAAGTTGAAGTAAAAGACATGGATATGATGCAGCAAGTCAAGTCTAACAAT
HP301      ATAAAGGTACAAGTTGAAGTAAAAGACATGGATATGATGTAGCAAGTCGAGTCTAACGAT
OH7B       ATAAAGGTACAAGTTGAAGTAAAAGACATGGATATGATGTAGCAAGTCGAGTCTAACGAT
Tzi8       ATAAAGGTACAAGTTGAAGTAAAAGACATGGATATGATGTAGCAAGTCGAGTCTAACGAT
```

Figure S1 Sequence Alignment of *Helitron Hel1-331* between the lines of maize. The sequence alignment exhibits the splice site junctions of various alternatively spliced transcript isoforms (i-vii) of *Hel1-331* in inbred B73 that are shown in figure 2. The arrows point to the donor and acceptor splice sites, and the blue and gray shaded sequence flanking the splice sites represent exons and introns, respectively. Asterisks beneath the highlighted regions indicate conserved flanking sequences between all the inbred lines. The names of the inbred lines are indicated on the left.

```
B73         TCCAGCGTCCTCCACCCCGAATCGCCGCCCCGGCTCTCTCCATGGAAGGCGCGTCTGCGA
HP301       TCCAGCGTCCTCCACCCCGAATCGCCGCCCCGGCTCTCTCCATGGAAGGCGCGTCTGCGA
OH7B        TCCAGCGTCCTCCGCCCCGGATCGTTGCC--CCAGATCTCCATGGAAGGCGTGTCTGTGA
Tzi8        TCCAGCGTCCTCCGCCCCGGATCGTTGCC--CCAGATCTCCATGGAAGGCGTGTCTGTGA

B73         CAGCCCGATCTCCGTGGAAGGCGTGTCTTCCTCCCCAGATCGTCGTCCCGGCTCTCTCCA
HP301       CAGCCCGATCTCCGTGGAAGGCGTGTCCTCCTCCCCAGATCGTCGTCCCGGCTCTCTCCA
OH7B        CAGCCCGATCTCTGTGGAAGGCGCGTCCTCCTCCCCAGATCGTCGTCCCGGCTCTCTCCA
Tzi8        CAGCCCGATCTCTGTGGAAGGCGCGTCCTCCTCCCCAGATCGTCGTCCCGGCTCTCTCCA

B73         CGCCCGTGACAGCCTGCTCTCTATGGAAGGTGAGTCTGCGATGCTCACGTGGATTGGGTA
HP301       CGCCCGTGACAGCCTGCTCTCTATGGAAGGTGAGTCTGCGATGCTCACGTGGATTGGGTA
OH7B        CGCTCGTGACAGCCTGCTCTCTATGGAAGGTGAGTCCGCGATGCTCACGTGGATTGGGTA
Tzi8        CGCTCGTGACAGCCTGCTCTCTATGGAAGGTGAGTCCGCGATGCTCACGTGGATTGGGTA
                          ********************** *******
                                        ↑ Donor iii

B73         CCCCGCCCCCGCTCTCTCCATGGAAGGCGTGTCTCCGATAGCCGCATCCTCCCTTGCGAA
HP301       CCCCGCCCCCGCTCTCTCCATGGAAGGCGTGTCTCCGATAGCCGCATCCTCCCTTGCGAA
OH7B        CCCCGCCCCCGCTCTCTCCATGGAAAGTGTGTCTCCGATAGACGCATCCTCCCTTGCGAA
Tzi8        CCCCGCCCCCGCTCTCTCCATGGAAAGTGTGTCTCCGATAGACGCATCCTCCCTTGCGAA

B73         CCGCGTGCGTCATGGCAACACGGGCGAGGCGGTGGGGAGTCTGGTGGACGCGCATCACGG
HP301       CCGCGTGCGTCATGGCAACACGGGCGAGGCGGTGGGGAGTCTGGTGGACGCGCATCACGG
OH7B        CCGCGCACGTCATGGCAACACGGGCGAGGCAGTGGGGAGTCTGGCGGACGCGCATCACGG
Tzi8        CCGCGCACGTCATGGCAACACGGGCGAGGCAGTGGGGAGTCTGGCGGACGCGCATCACGG

B73         TGAGACGAACGGGGGCAAGGGCAAGGGATATATTTTCCATCCGGGGCGGATGTGGAGAGG
HP301       TGAGACGAACGGGGGCAAGGGCAAGGGATATATTTTCCATCCGGGGCGGATGTGGAGAGG
OH7B        TGAGGCGAACGGGGGCAAGGGCAAGGGATATATTTTCCATCCGGGGCGGATGTGGAGAGG
Tzi8        TGAGGCGAACGGGGGCAAGGGCAAGGGATATATTTTCCATCCGGGGCGGATGTGGAGAGG

B73         TTCTTCAAACTCTGTGACCCTGGTGAGTGCGCTCGATCTCTGGT----------------
HP301       TTCTTCAAACTCTGTGACCCTGGTGAGTGCGCTCGATCTCTGGT----------------
OH7B        TTCTTCAAACTCTGTGACCCTGGTGAGTGCGCTCGATCTCTTTGGTTCCAGGATGTGGAG
Tzi8        TTCTTCAAACTCTGTGACCCTGGTGAGTGCGCTCGATCTCTTTGGTTCCAGGATGTGGAG
                   ******************************
                                 ↑ Donor i, iv§, v

B73         ---------------CTTCCGTGGTTTTTCTGCTTGGGTTTCGTTGCTTTGGTGGGGGAT
HP301       ---------------CTTCCGTGGTTTTTCTGCTTGGGTTTCGTTGCTTTGGTGGGGGAT
OH7B        TGATAGTCGAGGAGGCTTCCGTGGTTTTTCTGCTTGGGTTTCGTTGCTTTGGTGGGGGAT
Tzi8        TGATAGTCGAGGAGGCTTCCGTGGTTTTTCTGCTTGGGTTTCGTTGCTTTGGTGGGGGAT

B73         TGGATCATTGGAAGCTAAATTTGGAGAAATCTCCCTTTTCTCTGTCTCCTCTACCCTCAC
HP301       TGGATCATTGGAAGCTAAATTTGGAGAAATCTCCCTTTTCTCTGTCTCCTCTACCCTCAC
OH7B        TGGATCGTTGGAAGCTAAATTTAGAGAAATTT-CCCTTTCTCTGTCTCCTCTACCCTCAC
Tzi8        TGGATCGTTGGAAGCTAAATTTAGAGAAATTT-CCCTTTCTCTGTCTCCTCTACCCTCAC
                                                                       *

B73         TCGACATGCTTCAGATTCGATTATTGGTGGCCTTACAGCTTTCGTTAATGCTCTGCTTGT
HP301       TCGACATGCTTCAGATTCGATTATTGGTGGCCTTACAGCTTTCGTTAATGCTCTGCTTGT
OH7B        TCGACATGCTTCAGATTCGATTATTGGTGGCCTTGCAGCTTTCGTTAATGCTCTGCTTGT
Tzi8        TCGACATGCTTCGGATTCGATTATTGGTGGCCTTGCAGCTTTCGTTAATGCTCTGCTTGT
            ************ ***************  **** ***********************
                          ↑ Acceptor v                  ↑ Donor ii
```

```
B73          GCTTGATTGTAGCTGTTCGAATTTGGTGAGAGATTGGACCCCGTGATGACAGTTTTATGT
HP301        GCTTGATTGTAGCTGTTCGAATTTGGTGAGAGATTGGACCCCGTGATGACAGTTTTATGT
OH7B         GCTTGATTGTAGCTGTTCGAATTTGGTGAGAGATTGGACCCCGTGATGACAGTTTTATGT
Tzi8         GCTTGATTGTAGCTGTTCGAATTTGGTGAGAGATTGGACCCCGTGATGACAGTTTTATGT
                       ******************************
                                     ↑ Donor vi

B73          TGGTTGGAACTGTGGAACCAGTGGCTGACATATAATTTTTTTTGCTATTAGGTTGTTACT
HP301        TGGTTGGAACTGTGGAACCAGTGGCTGACATATAATTTTTTTTGCTATTAGGTTGTTACC
OH7B         TGGTTGGAACTATGGAACCAGTGGCTGACATATAATTTTTTTTGCTATTAGGTTGTTACT
Tzi8         TGGTTGGAACTATGGAACCAGTGGCTGACATATAATTTTTTTTGCTAGTAGGTTGTTACT
                                                   ************ ***********
                                                                 ↑ Acceptor vi

B73          TGTTAGCGCTATGGGGTTGCTATCAATCTTGTATTGACAGCTTGCTTGCAGAAAATCACT
HP301        TGTTAGCGCTATGGGGTTGCTATCAATCTTGTATTGACAGCTTGCTTGCAGAAAATCACT
OH7B         TGTTAGCGCTATGGGGTTCCCATCAATCTTGTATTGACAGCTTGCTTGCAGAAAATCGCT
Tzi8         TGTTAGCGCTATGGGGTTCCCATCAATCTTGTATTGACAGCTTGCTTGCAGAAAATCGCT
             ******

B73          AGATATTTTTGGCTGATCGGCGCCGTGGAATTTCGATTGTGTACTGGGTGCTTGGTTTCT
HP301        AGATATTTTTGGCTGATCGGCGCCGTGGAATTTCGATTGTGTACTGGGTGCTTGGTTTCT
OH7B         AGATATTTTTGGCTGATCGGTGCCGTGGAAAATGGACTGTGTACTGGGTGCTTGGTTTCT
Tzi8         AGATATTTTTGGCTGATCGGTGCCGTGGAAAATGGACTGTGTACTGGGTGCTTGGTTTCT

B73          ATATGACTAGGCAGTTTGCTCTTCCATCTATCGCACAGCGCCACATCTTGGCTGGTCTGG
HP301        ATATGACTAGGCAGTTTGCTCTTCCATCTATCGCACAGCGCCACATCTTGGCTGGTCTGG
OH7B         ATATGACTAGGCAGTTTGCTCTTCCATCTATCGCACAGCGCCACATCTTGGCTGGTCTGG
Tzi8         ATATGACTAGGCAGTTTGCTCTTCCATCTATCGCACAGCGCCACATCTTGGCTGGTCTGG

B73          CATCAGCAGTTGACGGTCTGAGTGCACCATATGAAAGAGCATCATGTATTCATGAAAGAC
HP301        CATCAGCAGTTGACGGTCTGAGTGCACCATATGAAAGAGCATCATGTATTCATGAAAGAC
OH7B         CATCAACAGTTGACGGTCTGAGTGCACCATATGAAAGAGCATCACATATTCATGAAAGAC
Tzi8         CATCAACAGTTGACGGTCTGAGTGCACCATATGAAAGAGCATCACGTATTCATGAAAGAC

B73          ATGTAGTGAACTGGCTGTGGGCTGCAGGCTGTCATCCGTTTGGACCATTCTCGAACTCCT
HP301        ATGTAGTGAACTGGCTGTGGGCTGCAGGCTGTCATCCGTTTGGACCATTCTCGAACTCCT
OH7B         ATGTAGTGAACTGGCTGTGGGCTGCAGGCTGTCATCCGTTTGGACCATTCTCGAACTCCT
Tzi8         ATGTAGTGAACTGGCTGTGGGCTGCAGGCTGTCATCCGTTTGGACCATTCTCGAACTCCT

B73          CCCAGATCAGTCAAATTCTTTAGGATGTAGCACTGGTAATGTTATCAACAACATTT-TTT
HP301        CCCAGATCAGTCAAATTCTTTAGGATGTAGCACTGGTAATGTTATCAACAACATTT-TTT
OH7B         CCCAGATCAGTCAAATTCTTCAGGATGTAGCACTGGTAATGTTATCAACAACATTTTTTT
Tzi8         CCCAGATCAGTCAAATTCTTCAGGATGTAGCACTGGTAATGTTATCAACAACATTTTTTT
                                  *****************************
                                               ↑ Donor vii

B73          TATTTCTATGATATGTACAATGAGTAGGCAGGGAGCGAGGGAGGTCAATGCTCCTATGTC
HP301        TATTTCTATGATATGTACAATGAGTAGGCAGGGAGCGAGGGAGGTCAATGCTCCTATGTC
OH7B         TAATTCTATGATATGTACAATGAGTAGGCAGGGAGCGAGGGAGGTCAATGCTCCTATGTC
Tzi8         TATTTCTATGATATGTACAATGAGTAGGCAGGGAGCGAGGGAGGTCAATGCTCCTATGTC

B73          GAAGTGCAGCTTAAAGTAGAAAATCTGTTCATGCTTCCAATGGTTTGCACAGTGCATAGG
HP301        GAAGTGCAGCTTAAAGTAGAAAATCTGTTCATGCTTCCAATGGTTTGCACAGTGCATAGG
OH7B         GAAGTGTAGCTTAAAGTAGAAAATCTGTTCATGCTTCCAATGGTTTGCACAGTGCATAGG
Tzi8         GAAGTGTAGCTTAAAGTAGAAAATCTGTTCATGCTTCCAATGGTTTGCACAGTGCATAGG
```

```
B73        TATGCAAACCAACAAAGGAAAACGAGGCCAAACTATGCCCCAATTGTTTCTGTCTGAGTT
HP301      TATGCAAACCAACAAAGGAAAACGAGGCCAAACTATGCCCCAATTGTTTCTGTCTGAGTT
OH7B       TATGCAAACCAACAAAGGAAAACGAGGCCAAACTATGCCCCAATTGTTTCTGTCTGAGTT
Tzi8       TATGCAAACCAACAAAGGAAAACGAGGCCAAACTATGCCCCAATTGTTTCTGTCTGAGTT

B73        TGCCAGTTTCAATTTTTTGGATCAACATAACTTGGGTATGTTGGAAATAATCCCGTGTCT
HP301      TGCCAGTTTCAATTTTTTGGATCAACATAACTTGGGTATGTTGGAAATAATCCCGTGTCT
OH7B       TGCCAGTTTCAATTTTTTGGATCAACATAACTTGGGTATGTTGGAAATAATCCCATGTCT
Tzi8       TGCCAGTTTCAATTTTTTGGATCAACATAACTTGGGTATGTTGGAAATAATCCCATGTCT

B73        AGGTTCATAGCATTTACTTTAGCTAAATTTCAGCATGGAATAATAGGAAGTAATATGCTG
HP301      AGGTTCATAGCATTTACTTTAGCTAAATTTCAGCATGGAATAATAGGAAGTAATATGCTG
OH7B       AGGTTCATAGCATTTACTTTAGCTAAATTTCAGCATGGAATAATAGGAAGTAATATGCTA
Tzi8       AGGTTCATAGCATTTACTTTAGCTAAATTTCAGCATGGAATAATAGGAAGTAATATGCTA

B73        ATTAGTTGAGTAATATCCTGCATTTATTAGGGGCTATCATTGCTTTATTTGTAGCTCACA
HP301      ATTAGTTGAGTAATATCCTGCATTTATTAGGGGCTATCATTGCTTTATTTGTAGCTCACA
OH7B       ATTAGTTGAGTAATATCCTGCATTTATTAGGGGCTATCATAGCTTTATTTGTAGCTCACA
Tzi8       ATTAGTTGAGTAATATCCTGCATTTATTAGGGGCTATCATAGCTTTATTTGTAGCTCACA

B73        TTGGTTATAATAGTAGTAGTAGAAGAGAAAATAGAAAAGGCAAGCCATTCCAGCCACCAA
HP301      TTGGTTATAATAGTAGTAGTAGAAGAGAAAATAGAAAAGGCAAGCCATTCCAGCCACCAA
OH7B       TTGGTTATAATAGTAGTAGTAGAAGAGAAAATAGAAAAGGCAAGCCATTCCAGCCACCAG
Tzi8       TTGGTTATAATAGTAGTAGTAGAAGAGAAAATAGAAAAGGCAAGCCATTCCAGCCACCAG

B73        AAACACTTGTACTATGTTCCTGTGACCTTTTGGAGGCTTTCAACCTGTCAGGAATCAGGA
HP301      AAACACTTGTACTATGTTCCTGTGACCTTTTGGAGGCTTTCAACCTGTCAGGAATCAGGA
OH7B       AAACACTTGTACTGTGTTCCTGTGACCTTTTGGAGGCTTTCAACCTGTCAGGAATCAGGA
Tzi8       AAACACTTGTACTGTGTTCCTGTGACCTTTTGGAGGCTTTCAACCTGTCAGGAATCAGGA

B73        TATTTGATGCATCCAATTATCTAAATCCAACAAATCTTGGTATTATGCTTACTGTATAAT
HP301      TATTTGATGCATCCAATTATCTAAATCCAACAAATCTTGGTATTATGCTTACTGTATAAT
OH7B       TATTTGATGCATCCAATTATCTAAATCCAACAAATCTTGGTATTATGCTTACTGTATAAT
Tzi8       TATTTGATGCATCCAATTATCTAAATCCAACAAATCTTGGTATTATGCTTATTGTATAAT

B73        CATGGTCGTATTATTTTTTAGTCTGTTGATATTTCACTGGCATAAATTGTTTCTTCTGTG
HP301      CATGGTCGTATTATTTTTTAGTCTGTTGATATTTCACTGGCATAAATTGTTTCTTCTGTG
OH7B       CATGGTCGCATTATTTTTTAGTCTGTTGATATTTCACTGGCATAAATTGTTTCTTCTGTG
Tzi8       CATGGTCGCATTATTTTTTAGTCTGTTGATATTTCACTGGCATAAATTGTTTCTTCTGTG

                                                   ⬇ Acceptor i, iii, iv§, vii
B73        CTTTCATATCCACTATGTAACCCATGTTTTATTGTTTGTAGAGAACTACAATTTATGCTC
HP301      CTTTCATATCCACTATGTAACCCATGTTTTATTGTTTGTAGAGAACTACAATTTATGCTC
OH7B       CTTTCATATCCACTATGTAACCCATGTTTTATTGTTTGTAGAGAACTACAATTTATGCTC
Tzi8       CTTTCATATCCACTATGTAACCCATGTTTTATTGTTTGTAGAGAACTACAATTTATGCTC
                                     **********************************
                                                            ⬆ Acceptor ii
B73        AAGTCAATGCTGCTCTTCGCAAAATAAGAGACACATCAGAGGTCCTACATGCAATTCAGC
HP301      AAGTCAATGCTGCTCTTCGCAAAATAAGAGACACATCAGAGGTCCTACATGCAATTCAGC
OH7B       AAGTCAATGCTGCTCTTCGCAAAATAAGAGACACATCAGAGGTCCTACATGCAATTCAGC
Tzi8       AAGTCAATGCTGCTCTTCGCAAAATAAGAGACACATCAGAGGTCCTACATGCAATTCAGC
           ******
```

```
B73       CTACTTTTATTCACTTATTTCCTCAAATGCACAGAAAGTATAACTACTATCTCTGGTCTG
HP301     CTACTTTTATTCACTTATTTCCTCAAATGCACAGAAAG-TTAACTACTATCTCTGGTCTG
OH7B      CTACTTTTATTCACTTATTTCCTCAAATGCACAGAAAGTATAACTACTATCTCTGGTCTG
Tzi8      CTACTTTTATTCACTTATTTCCTCAAATGCACAGAAAGTATAACTACTATCTCTGGTCTG

B73       GTGCAGTTTGTCCAATCTTTTGCATCAGAGCATCTTAAGACGCCATTAGAAGAACTTGTG
HP301     GTGCAGTTTGTCCAATCTTTTGCATCAGAGCATCTTAAGACGCCATTAGAAGAACTTGTG
OH7B      GTGCAGTTTGTCCAATCTTTTGCATCAGAGCATCTTAAGATGCCATTAGAAGAACTTGTG
Tzi8      GTGCAGTTTGTCCAATCTTTTGCATCAGAGCATCTTAAGATGCCATTAGAAGAACTTGTG

B73       AAAGGTAATAAAAACAAGTCCACCA---CTGAGTTATGGGTAGAGAAATTCTATAAGAAG
HP301     AAAGGTAATAAAAACAAGTCCACCA---CTGAGTTATGGGTAGAGAAATTCTATAAGAAG
OH7B      AAAGGTAATAAAAACAAGTC-CACCACTGAGTT--ATGGGTAGAGAAATTCTATAAGAAG
Tzi8      AAAGGTAATAAAAAACAAGTCCACCGCTGAGTTATTGGGTAGAGAAAATTCTATAAGAAG

B73       GTAACCAATCTACCGGAGCCTTTTCCCCATGATCTTGTTGAGAAGTTAGAAGAATACCTG
HP301     GTAACCAATCTACCGGAGCCTTTTCCCCATGATCTTGTTGAGAAGTTAGAAGAATACCTG
OH7B      GTAACCAATCTACCGGAGCCTTTTCCCCATGATCTTGTTGAGAAGTTAGAAGAATACCTG
Tzi8      GTAACCAATCTACCGGAGCCTTTTCCCCATGATCTTGTTGAGAAGTTAGAAGAATACCTG

B73       GATGTAAGTATTATATACCATACATTGCATAAGTGTACTTTCATGATGAAATAATGCATA
HP301     GATGTAAGTATTATATACCATACATTGCATAAGTGTACTTTCATGATGAAATAATGCATA
OH7B      GATGTAAGTATTATATACCATACATTGCATAAGTGTACTTTCATGATGAAATAATGCATA
Tzi8      GATGTAAGTATTATATACCATACATTGCATAAGTGTACTTTCATGATGAAATAATGCATA

B73       TATATGTTCTAATTTTCCTTCATCTATGGTGCCCCTTGTAGAAACTTGAAGGGCAACCCG
HP301     TATATGTTCTAATTTTCCTTCATCTATGGTGCCCCTTGTAGAAACTTGAAGGGCAACCCG
OH7B      TATATGTTCTAATTTTCCTTCATCTATGGTGCCCCTTGTAGAAACTTGAAGGGCAACCCG
Tzi8      TATATGTTCTAATTTTCCTTCATCTATGGTGCCCCTTGTAGAAACTTGAAGGGCAACCCG

B73       TGGACTTGTCTTCCTTATTGTATGACCACCAGCTGATCGATGCATATCAAAACAGTACCG
HP301     TGGACTTGTCTTCCTTATTGTATGACCACCAGCTGATCGATGCATATCAAAACAGTACCG
OH7B      TGGACTTGTCTTCCTTATTGTATGACCACCGGCTGATCGATGCATATCAAAGCAGTACCG
Tzi8      TGGACTTGTCTTCCTTATTGTATGACCACCGGCTGATCGATGCATATCAAAGCAGTACCG

B73       ACATCCTGCAAAGCACTATTTTTACACAACAGTATGTTTATATAGAGATGTTATATTTGC
HP301     ACATCCTGCAAAGCACTATTTTTACACAACAGTATGTTTATATAGAGATGTTATATTTGC
OH7B      ACATCCTGCAAAGCACTATTTTTACACAACAGTATGTTTATACAGGGATGCTATATTTGC
Tzi8      ACATCCTGCAAAGCACTATTTTTACACAACAGTATGTTTATACAGGGATGCTATATTTGC

B73       ATCTGGCTACTCTTGTGCTATCTTTTTGAAACTCATTATTTTCATTTTGTTTCATTCGAA
HP301     ATCTGGCTACTCTTGTGCTATCTTTTTGAAACTCATTATTTTCATTTTGTTTCATTCGAA
OH7B      ATCTGGCTACTCTTGTGCTATCTTTTTGAAACTCATTATTTTCATTTTGTTTCATTCGAA
Tzi8      ATCTGGCTACTCTTGTGCTATCTTTTTGAAACTCATTATTTTCATTTTGTTTCATTCGAA

B73       ACATTTTTACCCTTTCCCCCCTTCTCTTGACAGGTATGTGGAACGTGTTCTAGCTAACGA
HP301     ACATTTTTACCCTTTCCCCCCTTCTCTTGACAGGTATGTGGAACGTGTTCTAGCTAACGA
OH7B      ACATTTTTACCCTTTCCCCCCTTCTCTTGACAGGTATGTGGAACGTGTTCTAGCTAACGA
Tzi8      ACATTTTTACCCTTTCCCCCCTTCTCTTGACAGGTATGTGGAACGTGTTCTAGCTAACGA
```

```
B73          GAGAGACAAGATGAAATGCTGTAGCATTGAATATAGTCATCCAAAGCAGTCATCCCAGGC
HP301        GAGAGACAAGATGAAATGCTGTAGCATTGAATATAGTCATCCAAAGCAGTCATCCCAGGC
OH7B         GAGAGACAAGATGAAATGCTGTAGCATTGAATATAGTCATCCAAAGCAGTCATCCCAGGC
Tzi8         GAGAGACAAGATGAAATGCTGTAGCATTGAATATAGTCATCCAAAGCAGTCATCCCAGGC

B73          TTTTGTCTATGGAGGGATCCTTCTGGCTGGATTTATTGTGTATTCACTGGTTATTTTCTT
HP301        TTTTGTCTATGGAGGGATCCTTCTGGCTGGATTTATTGTGTATTCACTGGTTATTTTCTT
OH7B         TTTTGTCTATGGAGGGATCCTTCTGGCTGGATTTCTTGTGTATTCACCGGTTATTTTCTT
Tzi8         TTTTGTCTATGGAGGGATCCTTCTGGCTGGATTTCTTGTGTATTCACCGGTTATTTTCTT
                                                  ************ ************
                                                               ↑ Donor viii§

B73          CTCTTCACCTGTTCGCTAATTTTATGTCCAGATGAAGAAAGTCCTGGATGCTTTATGGTA
HP301        CTCTTCACCTGTTCGCTAATTTTATGTCCAGATGAAGAAAGTCCTGGATGCTTTATGGTA
OH7B         CTCTTCACCTGTTCGCTAATTTTATGTCCAGATGCAGAAAGTCCTGGATGCTTTATGGTA
Tzi8         CTCTTCACCTGTTCGCTAATTTTATGTCCAGATGCAGAAAGTCCTGGATGCTTTATGGTA
             ****

B73          GGCTAGGTTTGTCTAGGGTTGTCAATCTTCATGAGGGAATGACCTAGACCGTCTTGGATA
HP301        GGCTAGGTTTGTCTAGGGTTGTCAATCTTCATGAGGGAATGACCTAGACCGTCTTGGATA
OH7B         GGCTAGGTTTGTCTAGGGTTGTCAATATTCATGAGGGAATGACCTAGACTGTCTTGGATA
Tzi8         GGCTAGGTTTGTCTAGGGTTGTCAATATTCATGAGGGAATGACCTAGACTGTCTTGGATA

B73          TAGAAGGGCTTTGGCACAGTTTTAGTGCGCTAT-TTCGCGATACACAAGCAATCAGACGT
HP301        TAGAAGGGCTTTGGCACAGTTTTAGTGCGCTAT-TTCGCGATACACAAGCAATCAGACGT
OH7B         TAGAAGGGCTTTGGCACAGTTTTAGTGCGCTAT-TTCTCGATACACAAGCAATCAGACGG
Tzi8         TAGAAGGGCTTTGGCACAGTTTTAGTGCGCTATTTTCTCGATACACAAGCAATCAGACGG

B73          CTGATATATATGTAACTGATTCTTGTCTGCGATAAACTATAGTCGATTTCACAGAAGAGT
HP301        CTGATATATATGTAACTGATTCTTGTCTGCGATAAACTATAGTCGATTTCACAGAAGAGT
OH7B         CTGATATATATGTAACTGATTCTTGTCTGCGATAAACTATAGTTGATTTCACAGAAGAGT
Tzi8         CTGATATATATGTAACTGATTCTTGTCTGCGATAAACTATAGTTGATTTCACAGAAGAGT

B73          AGGTGCATGCTATGAAAATATGAAATACTTTCCCCC--ATTTTGCAACTTGTTACTGCAG
HP301        AGGTGCATGCTATGAAAATATGAAATACTTTCC-CC--ATTTTGCAACTTGTTACTGCAG
OH7B         TGGTGCATGCTATGAAAATATGAAATACTTACCCCC--ATTTTGCAACTTGTTACTGTAG
Tzi8         TGGTGCATGCTATGAAAATATGAAATACTTACCCCCCATTTTTGCAACTTGTTACTGTAG

B73          AACTGCACATAACACCACTGACAGAAACCCGTTTGTTATTAAGCCCCCCAGCTTCATGTG
HP301        AACTGCACATAACACCACTGACAGAAACCCGTTTGTTATTAAGC-CCCCAGCTTCATGTG
OH7B         AACTGCACATAACACCACTGACAGAAACCCATTTGTTAATAAGCCCCCCAGTTTCATGTG
Tzi8         AACTGCACATAACACCACTGACAGAAACCCATTTGTTAATAAGCCCCCCAGTTTCATGTG

B73          GACCAGCCAGCAGACCTGTGGCCGTGGAGTGGTGCAAGGGCGTAGTCGCTTTATTTCATG
HP301        GACCAGCCAGCAGACCTGTGGCCGTGGAGTGGTGCAAGGGCGTAGTCGCTTTATTTCACG
OH7B         GACCAGCCAGCAGACCTGTGGCCGTGGAGTGGTGCAAGGGCGTAGTCGCTTTATTTCATG
Tzi8         GACCAGCCAGCAGACCTGTGGCCGTGGAGTTGCGCAAGGGCGAGTCGCTTTAT-T-CATG

B73          GCAATCATCTCCAAAAATT-ATTTTCCTTGGCTCGTGT-GTTTTTTATCCTTTTGAAAAT
HP301        GCAATCATCTCCAAAAATT-ATTTTCCTTGGCTCGTGT-GTTTTTTATCCTTTTGAAAAT
OH7B         GCAATCAT-CTCCAAAAAATTATTTTCCTTAGCTCGTGT-GTTTTTTATCCTTTTGAAAAT
Tzi8         GCAATCATCTCCAAAAAATTATTTTCCTTAGCTCGTGTGTTTTTTTATCCTTTTGAAAAT
```

```
B73        TGCCTGACGCCAAGGCTCCACCATACGCCTGAAGCGCTGAGGCCTCAATGTCCAGCGTGT
HP301      TGCCTGACGCCAAGGCTCCACCATACGCCTGAAGCGCTGAGGCCTCAATGTCCAGCGTGT
OH7B       TGCCTAACGCCAAGGCTCCACCGTACGCCTGAAGCGATGAGGCCTCAATGTCCAGCGTGT
Tzi8       TGCCTAACGCCAAGGCTCCACCGTACGCCTGAAGCGATGAGGCCTCAATGTCCAGCGTGT

B73        TTCAATATAAAAGATTGCTTTTAGCTAGGGCAATGGTGGTTGTGCAAGCAGTATTTAGGT
HP301      TTCAATATAAAAGATTGCTTTTAGCTAGGGCAATGGTGGTTGTGCAAGCAGTATTTAGGT
OH7B       TTCAATATAAAAGATTGCTTTTAGCTAGGGCAATGGTGGTTGTGCAAGAAGTACTTAGGT
Tzi8       TTCAATATAAAAGATTGCTTTTAGCTAGGGCAATGGTGGTTGTGCAAGAAGTACTTAGGT

B73        GCAAACGTGCAAGCAAGTTCTCTGGTCCGTTTTGATTTGTTCCATCCTGCTATATATTTT
HP301      GCAAACGTGCAAGCAAGTTCTCTGGTCCGTTTTGATTTGTTCCATCCTGCTATATATTTT
OH7B       GCAAACGTGCAAGCGAGTTCTCTGGTCCGTTTTGATTTGTTCCATCCCGCTATATATTTT
Tzi8       GCAAACGTGCAAGCGAGTTCTCTGGTCCGTTTTGATTTGTTCCATCCCGCTATATATTTT

B73        GCACCAGTTTATAATTATCTTTTGGCAC--ATGATCACTACATATGTGAGTTGGCTCCAA
HP301      GCACCAGTTTATAATTATCTTTTGGCAC--ATGATCACTACATATGTGAGTTGGCTCCAA
OH7B       GCACCAGTTTATAATTATCTTTTGGCACATATGATCACTACATATGTGAGTTGGCTCCAA
Tzi8       GCACCAGTTTATAATTATCTTTTGGCACATATGATCACTACATATGTGAGTTGGCTCCAA

B73        TAATTTAACTTTGATTCCATGGAACATTTTATTTTCATCCGGACACTTCTGTCTTTCTCA
HP301      TAATTTAACTTTGATTCCATGGAACATTTTATTTTCATCCGGACACTTCTGTCTTTCTCA
OH7B       TAATTTAACTTTGATTCCATGGAACATTTTATTTTCATCCGGACACTTATGTCTTTCTCA
Tzi8       TAATTTAACTTTGATTCCATGGAACATTTTATTTTCATCCGGACACTTATGTCTTTCTCA

B73        TAAAATTT-TTATTGCTTGTAAATTTACTATTGGTGCTCAAATATTCTTTG-CTTCATCA
HP301      TAAAATTT-TTATTGCTTGTAAATTTACTATTGGTGCTCAAATATTCTTTG-CTTCATCA
OH7B       TAAAATCTTTTATTGCTTGTAAATTTACTATTGGTGCTCAAATATTCTTTGCCTTCATCA
Tzi8       TAAAATCTTTTATTGCTTGTAAATTTACTATTGGTGCTCAAATATTCTTTGCCTTCATCA

B73        AAAAAATTCCTTGATCAGTTCTGGTGATTATAACAATTTTAGTTTTTTGGTTATAACTTT
HP301      AAAAAATTCCTTGATCAGTTCTGGTGATTATAACAATTTTAGTTTTTTGGTTATAACTTT
OH7B       AAAAAATTCCTTGATCAGTTCTGGTGATTAGAACAATTTTAGTTTTTTTGTTAGAACTTT
Tzi8       AAAAAATTCCTTGATCAGTTCTGGTGATTAGAACAATTTTAGTTTTTTTGTTAGAACTTT

B73        TTCCTTTCTCAGAACATTTCTCTTTGCTTGGAGCATTTTCCTTAGTATTTAGAACAATTT
HP301      TTCCTTTCTCAGAACATTTCTCTTTGCTTGGAGCATTTTCCTTAGTATTTAGAACAATTT
OH7B       TTCCTTTCTCAGAACATTTCTCTTTGCTTGGAGCATTTTCCTTAGTATTTAGAACAATTT
Tzi8       TTCCTTTCTCAGAACATTTCTCTTTGCTTGGAGCATTTTCCTTAGTATTTAGAACAATTT

B73        ATTTTTTGATCGA--TTTTTTTCTTTGCTCAGATCAATGCTTGTTGCAACATCATAGTGC
HP301      ATTTTTTGATCGA--TTTTTTTCTTTGCTCAGATCAATGCTTGTTGCAACATCATAGTGC
OH7B       ATTTTTTGATCGAATTTTTTTCTTTGCTCAGATCAATGCTTGTTGCAACATCATAGTGC
Tzi8       ATTTTTTGATCGAATTTTTTTCTTTGCTCAGATCAATGCTTGTTGCAACATCATAGTGC
                            ******************************
                                           ↑ Acceptor viii§

B73        ACCTGATGGGGAATCCAACATGTTTCACGTGACATGTAGGGGAAGGCATCGAAAAAGGTG
HP301      ACCTGATGGGGAATCCAACATGTTTCACGTGACATGTAGGGGAAGGCATCGAAAAAGGTG
OH7B       ACCTGATAGGGAATCCAACATGTTTCACGTGACATGTAGGGGAAGGCATCGAAAAAGTTG
Tzi8       ACCTGATAGGGAATCCAACATGTTTCACGTGACATGTAGGGGAAGGCATCGAAAAAGTTG
```

```
B73         AAGGTTGTAGAAAAGGGCCCTAAAAAGGGCACGATGAAAGCCCAACTTGCTAAGAGAGGT
HP301       AAGGTTGTAGAAAAGGGCCCTAAAAAGGGCACGATGAAAGCCCAACTTGCTAAGAGAGGT
OH7B        AAGGTTGTAGAAAAGAGCACGAT-----------GAAAAAGCCAACTTGCTAAGAGAGGT
Tzi8        AAGGTTGTAGAAAAGAGCACGAT-----------GAAAAAGCCAACTTGCTAAGAGAGGT

B73         AGGAAGGCTAAGAATTGATGACATGTTGATTTCCTCGTCAGAGAAAAAAATGCTATAGCA
HP301       AGGAAGGCTAAGAATTGATGACATGTTGATTTCCTCGTCAGAGAAAAAAATGCTATAGCA
OH7B        AGGAAGGCTAAGAATTGATGACATGTTGATTTCCTCGTCAGAGAAAAAAATGCTATAGCA
Tzi8        AGGAAGGCTAAGAATTGATGACATGTTGATTTCCTCGTTAGAGAAAAAAATGCTATAGCA
```

Figure S2 Alignment of *Helitron Hel1-332* between the maize inbred lines. The alignment shows the splice junctions of alternative splicing events (i – viii) of *Hel1-332* shown in figure 3. The blue and gray shaded sequences flanking the splice sites represent exons and introns, respectively. Asterisks represent the conserved sequences beneath the highlighted regions, while the dashed lines fill the gaps in the alignment. The inbred lines are indicated on the left. The intron retention at splice sites iv and viii are marked by §.

```
B73     TGTGAATCCTCGCCTAGGATGCCGACGGGCACGGCGCCCACGGTGGTCACCTTCTGCGCC
HP301   TGTGAATCCTCGCCTAGGATGCCGACGGGCACGGCGCCCACGGTGGTCACCTTCTGCGCC
OH7B    TGTGAATCCTCGCCTAGGATGCCGACGGGCACGACGCCCACGGTGGTCACCTTCTGCGCC
Tzi8    TGTGAATCCTCGCCTAGGATGCCGACGGGCACGGCGCCCACGGTGGTCACCTTCTGCGCC


B73     TCTTCGAGGTGGTGCTCATCGGCTCCATAGATCTGGCACCATGCTTAGCCGACCATGGCG
HP301   TCTTCGAGGTGGTGCTCATCGGCTCCATAGATCTGGCGCCATGCTTAGCCGACCATGGCG
OH7B    TCTTCGAGGTGGTGCTCATCGGCTCCATAGATCTGGCACCATGCTTAGCCGACCATGGCG
Tzi8    TCTTCGAGGTGGTGCTCATCGGCTCCATAGATCTGGCACCATGCTTAGCCGACCATGGCG


B73     AGGCCATCAAGGAGGACACCGGCCACGATCCGTCAGGTTTCTCTCCCTCCTCTTTCCCTC
HP301   AGGCCATCAAGGAGGACACCGGCCACGATCCGTCAGGTTTCTCTCCCTCCTCTTTCCCTC
OH7B    AGGCCATCAAGGAGGACATCGACCACGATCCGTCAGGTTTCTCTCCCTCCTCTTTCCCTC
Tzi8    AGGCCATCAAGGAGGACACCGGCCACGATCCGTCAGGTTTCTCTCCCTCCTCTTTCCCTC
                             ******************************
                                             ↑ Donor i

B73     TGTGTCTGTGATGTCGGGGTTGGATATGCATTGCAATGGGGGGCGTGATGGTGCGGTTGG
HP301   TGTGTCTGTGATGTCGGGGTTGGATATGCATTGCAATGGGGGGCGTGATGGTGCGGTTGG
OH7B    TGTGTCTGTGATGTCGGGGTTGGATATGCATTGCAATGGGGG-CGTGATGGTGCAGTTGG
Tzi8    TGTGTCTGTGATGTCGGGGTTGGATATGCATTGCAATGGGGGGCGTGATGGTGCGGTTGG


B73     AACTCATAACCATCCCCAATCAGTGTGAAGCGGTGGTGTGGGTGTCCCACTTGGGAGACT
HP301   AACTCATAACCATCCCCAATCAGTGTGAAGCGGTGGTGTGGGTGTCCCACTTGGGAGACT
OH7B    AACTCATAACCGTCCCCCATCCGTGTGAAGCGGTGGTGTGGGTGTCCCACTTGGGAGACT
Tzi8    AACTCATAACCATCCCCAATCAGTGTGAAGCGGTGGTGTGGGTGTCCCACTTGGGAGACT


B73     CGAGCACTGCGCATCCGGCAGCTTGTCTGGTGGGGGTCTATAGGGATTTTTTT-ACTTTC
HP301   CGAGCACTGCGCATCCGGCAGCTTGTCTGGTGGGGGTCTATAGGGATTTTTTT-ACTTTC
OH7B    CGGGCACTTCGCATCCGGCAGCTTGTCTGGTGGGGGTCTATAGGGATTTTTTTTACTTTC
Tzi8    CGAGCACTGCGCATCCGGCAGCTTGTCTGGTGGGGGTCTATAGGGATTTTTTT-ACTTTC


B73     TCTACTCTCAACTCGAGTGTTTCAAAAGCATCTTACACCCGTCTGTCAGATGGCTTTAAA
HP301   TCTACTCTCAACTCGAGTGTTTCAAAAGCATCTTACACCCGTCTGTCAGATGGCTTTAAA
OH7B    TCTACTCTCAACTCTAGTGTTTCAAAAGCATCTTACACCCGACTGTCAGATGGCTTTAAA
Tzi8    TCTACTCTCAACTCGAGTGTTTCAAAAGCATCTTACACCCGTCTGTCAAATGGCTTTAAA


B73     ACACTCGAAACTGGATAGATAACAGAATCGAGTGTTTATAGGCTATGGAACACTCAATTC
HP301   ACACTCGAAACTGGATAGATAACAGAATCGAGTGTTTATAGGCTATGGAACACTCAATTC
OH7B    ACACTCGAAACTAGATAGATAACAGAATCGAGTGTTTATAGGCTATGGAAAGCTCAATTC
Tzi8    ACACTCGAAACTGGATAGATAACAGAATCGAGTGTTTATAGGCTATGGAACACTCAATTC


B73     TCATATCAGATAGAAAAAAA-CAAACCAAATTATTTCTATGGAAGCCAAATTGGAAGGTT
HP301   TCATATCAGATAGAAAAAAA-CAAACCAAATTATTTCTATGGAAGCCAAATTGGAAGGTT
OH7B    TCATATCAGATAGAAAAAAAACTAACCAAATTATTTCTATGGAAGCCAAATTGGAAGGTT
Tzi8    TCATATCAGATAGAAAAAAA-CAAACCAAATTATTTCTATGGAAGCCAAATTGGAAGGTT


B73     TGCAGAGGGGAACCAAATAAAACATGCATACAAACAAAAAATAGAACAACAAGAAATAAA
HP301   TGCAGAGGGGAACCAAATAAAACATGCATACAAACAAAAAATAGAACAACAAGAAATAAA
OH7B    TGCAGAGGGGAACCAAATAAAACATGCATACAAACAAAAAATAGAACAGCAAGAAATAAA
Tzi8    TGCAGAGGGGAACCAAATAAAACATGCATACAAACAAAAAATAGAACAACAAGAAATAAA
```

```
B73     CAGATAACTGTTCGTGCAATCCAAATGATCATTCTCAATAATAAACAACCAATTTACATC
HP301   CAGATAACTGTTCGTGCAATCCAAATGATCATTCTCAATAATAAACAACCAATTTACATC
OH7B    CAGATAACTGTTCGTGCAATCCAAATGATCATTCTCAATAATAAACAACCAATTTACATC
Tzi8    CAGATAACTGTTCGTGCAATCCAAATGATCATTCTCAATAATAAACAACCAATTTACATC

B73     AGTCGGATACACAAC----ACACAAACACAGATAGTAAAGCATGATATAGTAAGATGTAA
HP301   AGTCGGATACACAAC----ACACAAACACAGATAGTAAAGCATGATATAGTAAGATGTAA
OH7B    AGTCGGATACACAACTTACACACAAACACAGATAGTAAAGCATGCTATAGTAAGATGTAA
Tzi8    AGTCGGATACACAAC----ACACAAACACAGATAGTAAAGCATGATATAGTAAGATGTAA

B73     TACGGAACTACTGGGCCAAAGCAGACCGGACGTAATCAAATCGAAGAAGGGATTCACAGA
HP301   TACGGAACTACTGGGCCAAAGCAGACCGGACGTAATCAAATCGAAGAAGGGATTCACAGA
OH7B    TACGGAACTACTGGGCCAAAGCAGACCGGACGTAATCAAATCGAAGAAGGGATTCACAGA
Tzi8    TACGGAACTACTGGGCCAAAGCAGACCGGACGTAATCAAATCGAAGAAGGGATTCACAGA

B73     TTCAAATAAGAGATGGAATTGTCTGGTGCCTTGGATTGCTGATTGAGGTGATGCAGGGCC
HP301   TTCAAATAAGAGATGGAATTGTCTGGTGCCTTGGATTGCTGATTGAGGTGATGCAGGGCC
OH7B    TTCAAATAAGAGATGGAATTGTCTGGTGCCTTGGATCGCTGATTGAGGTGATGC------
Tzi8    TTCAAATAAGAGATGGAATTGTCTGGTGCCTTGGATTGCTGATTGAGGTGATGCAGGGCC

B73     GGTCCTGAGGTTTTATGGACCCGGGGCAAAACAAAAAATTGGGGCCCTTTAAAACTGTAT
HP301   GGTCCTGAGGTTTTATGGACCCGGGGCAAAACAAAAAATTGGGGCCCTTTAAAACTGTAT
OH7B    ------------------------------------------------------------
Tzi8    GGTCCTGAGGTTTTATGGACCCGGGGCAAAACAAAAAATTGGGGCCCTTTAAAACTGTAT

B73     ATATATTTTTTACTTTCTTCTAAGTACATAAGCAAAATTTTGAAAGATAAAAGTCTATAA
HP301   ATATATTTTTTACTTTCTTCTAAGTACATAAGCAAAATTTTGAAAGATAAAAGTCTATAA
OH7B    ------------------------------------------------------------
Tzi8    ATATATTTTTTACTTTCTTCTAAGTACATAAGCAAAATTTTGAAAGATAAAAGTCTATAA

B73     TATATCAAAATAACTTCAAATCTTTGTACATATTACTTCAAAAAAATTCTAACATTTCTT
HP301   TATATCAAAATAACTTCAAATCTTTGTACATATTACTTCAAAAAAATTCTAACATTTCTT
OH7B    ------------------------------------------------------------
Tzi8    TATATCAAAATAACTTCAAATCTTTGTACATATTACTTCAAAAAAATTCTAACATTTCTT

B73     GATGCAAAGTCATTGATAATAGTGTTATTATCAACCTCGTCTAATAATTTTTCTCAATAC
HP301   GATGCAAAGTCATTGATAATAGTGTTATTATCAACCTCGTCTAATAATTTTTCTCAATAC
OH7B    ------------------------------------------------------------
Tzi8    GATGCAAAGTCATTGATAATAGTGTTATTATCAACCTCGTCTAATAATTTTTCTCAATAC

B73     ATAAAGTAGCTAATTCGTTGAGACGATCTTGAGACATCGTTGACCTTAAATAGTTCTTCA
HP301   ATAAAGTAGCTAATTCGTTGAGACGATCTTGAGACATCGTTGACCTTAAATAGTTCTTCA
OH7B    ------------------------------------------------------------
Tzi8    ATAAAGTAGCTAATTCGTTGAGACGATCTTGAGACATCGTTGACCTTAAATAGTTCTTCA

B73     ACAATTTTAACTTTGAAAAGCTTATCTTAGTCGATGCCACAGTCACGTCACATGCACAGT
HP301   ACAATTTTAACTTTGAAAAGCTTATCTTAGTCGATGCCACAGTCACGTCACATGCACAGT
OH7B    ------------------------------------------------------------
Tzi8    ACAATTTTAACTTTGAAAAGCTTATCTTAGTCGATGCCACAGTCACGTCACATGCACAGT
```

```
B73     AAATAATATTCGATAAGTGATTGAAATATTTGGATACGAATCTATTTGCCGAACATATTA
HP301   AAATAATATTCGATAAGTGATTGAAATATTTGGATACGAATCTATTTGCCGAACATATTA
OH7B    ------------------------------------------------------------
Tzi8    AAATAATATTCGATAAGTGATTGAAATATTTGGATACGAATCTATTTGCCGAACATATTA

B73     AAAAATCTCCATAGAAGACATTAGTCTTTCAGGGCACGAGGCCGGTGGCCAGCAACAGGT
HP301   AAAAATCTCCATAGAAGACATTAGTCTTTCAGGGCACGAGGCCGGTGGCCAGCAACAGGT
OH7B    ------------------------------------------------------------
Tzi8    AAAAATCTCCATAGAAGACATTAGTCTTTCAGGGCACGAGGCCGGTGGCCAGCAACAGGT

B73     CGTCAGGCCGATGTCGATCGTCGGCCAGCCAGACGGACGAAACCAAGGCGGTGGCAGCAC
HP301   CGTCAGGCCGATGTCGATCGTCGGCCAGCCAGACGGACGAAACCAAGGCGGTGGCAGCAC
OH7B    ------------------------------------------------------------
Tzi8    CGTCAGGCCGATGTCGATCGTCGGCCAGCCAGACGGACGAAACCAAGGCGGTGGCAGCAC

B73     AACGATTAAAATTCTAGACACAATTTTAGTAAGTTAAATAATTGAAAAATAGGACCATTG
HP301   AACGATTAAAATTCTAGACACAATTTTAGTAAGTTAAATAATTGAAAAATAGGACCATTG
OH7B    ------------------------------------------------------------
Tzi8    AACGATTAAAATTCTAGACACAATTTTAGTAAGTTAAATAATTGAAAAATAGGACCATTG

B73     TAACTTTAGCAGGTCAGACCTAATACCTAAGACTATATATACATGTACACTACTTGAAGG
HP301   TAACTTTAGCAGGTCAGACCTAATACCTAAGACTATATATACATGTACACTACTTGAAGG
OH7B    ------------------------------------------------------------
Tzi8    TAACTTTAGCAGGTCAGACCTAATACCTAAGACTATATATACATGTACACTACTTGAAGG

B73     GCCCTTCCAAAATCGGGGGCCCGGTGCGGCCGCTCCAGTTGCCCCCCTCCAGGTCCGGCC
HP301   GCCCTTCCAAAATCGGGGGCCCGGTGCGGCCGCTCCAGTTGCCCCCCTCCAGGTCCGGCC
OH7B    ------------------------------------------------------------
Tzi8    GCCCTTCCAAAATCGGGGGCCCGGTGCGGCCGCTCCAGTTGCCCCCCTCCAGGTCCGGCC

B73     CTGGGTGATGCCAGGGATGAGCTAGAATTTCTTCAATACTTGATGCGTGTGTTGACTGTT
HP301   CTGGGTGATGCCAGGGATGAGCTAGAATTTCTTCAATACTTGATGCGTGTGTTGACTGTT
OH7B    -----------CAGGGATGAGCTAGAATTTCTTCAATACTTGATGCGTGTGTTGACTGTT
Tzi8    CTGGGTGATGCCAGGGATGAGCTAGAATTTCTTCAATACTTGATGCGTGTGTTGACTGTT

B73     GAAGACTACAGCCCATCATGCATTTAGCTTATTAAAATAATGGTACCATTTTTGTAATGG
HP301   GAAGACTACAGCCCATCATGCATTTAGCTTATTAAAATAATGGTACCATTTTTGTAATGG
OH7B    GAAGACTACAGCCCATCATGCATTTAGCTTATTAAAATAATGGTACCATTTTTGTAATGG
Tzi8    GAAGACTACAGCCCATCATGCATTTAGCTTATTAAAATAATGGTACCATTTTTGTAATGG

B73     CTTGATGAA---TCATTAAGCTGGAGTTTTGTAGTGGCTTATTTTGTCCATGCTCCATCC
HP301   CTTGATGAA---TCATTAAGCTGGAGTTTTGTAGTGGCTTATTTTGTCCATGCTCCATCC
OH7B    CTTGATGATCATTAATTAAGCTGGAGTTTTGTAGTGTCTTATTTTGTCCATGCTCCATCC
Tzi8    CTTGATGAA---TCATTAAGCTGGAGTTTTGTAGTGGCTTATTTTGTCCATGCTCCATCC

B73     AAAACAGATTCATACCCATGCCCTT-CTCTAATCTGATCAAGTTATTCTTATCTTGAGTT
HP301   AAAACAGATTCATACCCATGCCCTT-CTCTAATCTGATCAAGTTATTCTTATCTTGAGTT
OH7B    AAAACAGATTCATACCCATGCCCTT-CTCTAATCTGATCAAGCTATTCTTATCTTGAGTT
Tzi8    AAAACAGATTCATACCCATGCCCTTTCTCTAATCTGATCAAGTTATTCTTATCTTGAGTT
```

```
B73       CTTGGCTAACCTCAGCGTCTATCTGTTTGATTACTCATATTTCATAATAAATTGATGTAT
HP301     CTTGGCTAACCTCAGCGTCTATCTGTTTGATTACTCATATTTCATAATAAATTGATGTAT
OH7B      CTTGGCTAACCTCAGCGTCTATCTGTTTGATTACTCATATTCCATAATAAATTGATGTAT
Tzi8      CTTGGCTAACCTCAGCGTCTATCTGTTTGATTACTCATATTTCATAATAAATTGATGTAT


B73       GTTTCTGCAATTTTGC--------TTATACATGCCTATTTTCATTATGGAGTTACTTACT
HP301     GTTTCTGCAATTTTGCAATTTTGCTTATACATGCCTATTTTCATTATGGAGTTACTTACT
OH7B      GTTTCTGCAATTTTGC--------TTATACATGCCTATTTTCATTATGGAGTTACTTACG
Tzi8      GTTTCTGCAATTTTGC--------TTATACATGCCTATTTTCATTATGGAGTTACTTACT


B73       TATCTGTAATTTTGTTTGTCCGCCCTGTGCAGATGATGGAGCCCACGAGCCATTTTCACG
HP301     TATCTGTAATTTTGTTTGTCCGCCCTGTGCAGATGATGGAGCCCACGAGCCATTTTCACG
OH7B      TATCTGTAATTTTGTTTGTCCGTCCTGTGCAGATGATGGAGCCCACGAGCCATTTTCACG
Tzi8      TATCTGTAATTTTGTTTGTCCGCCCTGTGCAGATGATGGAGCCCACGAGCCATTTTCACG
                           ***** ************************
                                        ↑ Acceptor i

B73       TAAACTTCTTGAAGATAAGCCTCGGATCACGTATGTTTAGAATTAAAACTTTTAAGTTTT
HP301     TAAACTTCTTGAAGATAAGCCTCGGATCACGTATGTTTAGAATTAAAACTTTTAAGTTTT
OH7B      TAAACTTCTTGAAGATAAGCCTCGGATCACGTATGTTTAGAATTAGAACTTTTAAGTTTT
Tzi8      TAAACTTCTTGAAGATAAGCCTCGGATCACGTATGTTTAGAATTAAAACTTTTAAGTTTT
                         ******************************
                                        ↑ Donor ii

B73       TTGATGTATAAAATGAAAACAATGTGTGTCTTAGTCTGATGATGTTATTGGTTGACTATG
HP301     TTGATGTATAAAATGAAAACAATGTGTGTCTTAGTCTGATGATGTTATTGGTTGACTATG
OH7B      TTGATGTATAAAATGAAAACAATGTGTGTCTCAGTCTGATGATGTTATTGGTTGACTATG
Tzi8      TTGATGTATAAAATGAAAACAATGTGTGTCTTAGTCTGATGATGTTATTGGTTGACTATG


B73       TTGTGTATGATTTTGAAATACATTTAGAGAAGAAATGGTCCGTGGATATATGAGCAACAC
HP301     TTGTGTATGATTTTGAAATACATTTAGAGAAGAAATGGTCCGTGGATATATGAGCAACAC
OH7B      CTGTGTATGATTTTGAAATACATTTAGAGAAGAAATGGTCCGTGGATATATGAGCAACAC
Tzi8      TTGTGTATGATTTTGAAATACATTTAGAGAAGAAATGGTCCGTGGATATATGAGCAACAC
                      ******************************
                                     ↑ Acceptor ii

B73       TGAGCTTGAGACCGCTGTACATGCCTTTGGAAGTCGTTGTTCCAATATCTCCAGGGTGTA
HP301     TGAGCTTGAGACCGCTGTACATGCCTTTGGAAGTCGTTGTTCCAATATCTCCAGGGTGTA
OH7B      TGAGCTTGAGACTGCTGTACATGCCTTTGGAAGTCGTTGTTCCAATATCTCCAGGGTGTA
Tzi8      TGAGCTTGAGACCGCTGTACATGCCTTTGGAAGTCGTTGTTCCAATATCTCCAGGGTGTA
                                                          ************

B73       CAGGTACACGATGTATAATGTTTTACTTCTGTGATAATTACATTTGGAGCTTCTAAACAA
HP301     CAGGTACACGATGTATAATGTTTTACTTCTGTGATAATTACATTTGGAGCTTCTAAACAA
OH7B      CAGGTACACGATGTATAATGTTTTACTTCTGTGATAATTACATTTGGAGCTTCTAAACAA
Tzi8      CAGGTACACGATGTATAATGTTTTACTTCTGTGATAATTACATTTGGAGCTTCTAAACAA
          ******************
             ↑ Donor iii

B73       ACTTTTAAGACCATCAGTTTTTAAGGACATGTAAATGACTGTGTCATTGGTGGGTAGTAC
HP301     ACTTTTAAGACCATCAGTTTTTAAGGACATGTAAATGACTGTGTCATTGGTGGGTAGTAC
OH7B      ACTTTTAAGACCATCAGTTTTTAAGGACATGTAAATGACTGTGTCATTGGTGGGTAGTAC
Tzi8      ACTTTTAAGACCATCAGTTTTTAAGGACATGTAAATGACTGTGTCATTGGTGGGTAGTAC


B73       CGTAGTAATATAAGATCAGGAGCGACTGCGTCTTTGACAGACAAAGGCCCAGCATGGGCA
HP301     CGTAGTAATATAAGATCAGGAGCGACTGCGTCTTTGACAGACAAAGGCCCAGCATGGGCA
OH7B      TGTAGTAATATAAGATCAGGAGCGACTGCGTCTTTGACAGACAAAGGCCCAACATGGACA
Tzi8      CGTAGTAATATAAGATCAGGAGCGACTGCGTCTTTGACAGACAAAGGCCCAGCATGGGCA
```

```
B73     GCATCGTCATGAGAGCGAGAGCGGAGAAGGAGCTTTGGGAGATGGCTGGAGGCTGGAGCT
HP301   GCATCGTCATGAGAGCGAGAGCGGAGAAGGAGCTTTGGGAGATGGCTGGAGGCTGGAGCT
OH7B    GCATCGTCATGAGAGCGAGAGCGGAGAGGGAGCTTTGGGAGATGGCTGGAGGCTGGAGCT
Tzi8    GCATCGTCATGAGAGCGAGAGCGGAGAAGGAGCTTTGGGAGATGGCTGGAGGCTGGAGCT

B73     AAGAGCATGTCTCTCCTTATGGCTCCGGCTCCAGGGTTGTAGGGTCCCCTTAGTAGCGAT
HP301   AAGAGCATGTCTCTCCTTATGGCTCCGGCTCCAGGGTTGTAGGGTCCCCTTAGTAGCGAT
OH7B    AAGAGCATGTCTCTCCTTATGGCTCCGGCTCCAGGGTTGTAGGGTCCCCTTAGTAGCGAT
Tzi8    AAGAGCATGTCTCTCCTTATGGCTCCGGCTCCAGGGTTGTAGGGTCCCCTTAGTAGCGAT

B73     GTGGTCGTCATTATTTGTTTAGTCGTATGAGGTATGACCCCATTTTTTGTATTCTTGTTT
HP301   GTGGTCGTCATTATTTGTTTAGTCGTATGAGGTATGACCCCATTTTTTGTATTCTTGTTT
OH7B    GTGGTCGCCATTATTTGTTTAGTCGTATGAGGTATGACCCCATTTTTTGTATTCTTGTTT
Tzi8    GTGGTCGTCATTATTTGTTTAGTCGTATGAGGTATGACCCCATTTTTTGTATTCTTGTTT

B73     TTGGCCACCGTAAGTGGTTTTATCTTGTAACGGCCGGCATGGACCTTAACCACTTTCTTC
HP301   TTGGCCACCGTAAGTGGTTTTATCTTGTAACGGCCGGCATGGACCTTAACCACTTTCTTC
OH7B    TTGGCCACCTTAAGTGGTTTTATCTTGTAACGGTCGGCATGGACCTTAACCACTTTCTTC
Tzi8    TTGGCC-CCGTAAGTGGTTTTATCTTGTAACGGCCGGCATGGACCTTAACCACTTTCTTC

B73     TTAATATAATGACGCGCAGCTTTCCTGCGCTTCCGAGAAAAATAAAGGATTTAAAAATCA
HP301   TTAATATAATGACGCGCAGCTTTCCTGCGCTTCCGAGAAAAATAAAGGATTTAAAAATCA
OH7B    TTAATATAATGACGCACAGCTCTCCTGCGCTTCCGAGAAAAATAAAGGATTTAAAAATCA
Tzi8    TTAATATAATGACGCGCAGCTTTCCTGCGCTTCCGAGAAAAATAAAGGATTTAAAAATCA

B73     TTATCAACCATGAGAGAAACAACATTGGATGCAGTCATGTTGATAGGTAACAACAAATGG
HP301   TTATCAACCATGAGAGAAACAACATTGGATGCAGTCATGTTGATAGGTAACAACAAATGG
OH7B    TTATCAACCATGAGAGAAACAACATTGCATGCAGTCATGTTGATAGGTAACAGCAAATGG
Tzi8    TTATCAACCATGAGAGAAACAACATTGGATGCAGTCATGTTGATAGGTAACAACAAATGG

B73     ATAAAATGAAACATGTAGCTTTTTGAAAAAAAAACTAATCATGTAGCGCCAACTTATAAC
HP301   ATAAAATGAAACATGTAGCTTTTTGAAAAAAAAACTAATCATGTAGCGCCAACTTATAAC
OH7B    ATAAAATGAAACATGTAGCTTTTTGAAAAAAAA-CTAATCATGAAGCGCCAACTTATAAC
Tzi8    ATAAAATGAAACATGTAGCTTTTTGAAAAAAAAACTAATCATGTAGCGCCAACTTATAAC

B73     ATAATTATATCC--ATAGGATACATGTAGTAATTCTGTTTTACTTCAGATTGTTTTTGGA
HP301   ATAATTATATCCCCATAGGATACATGTAGTAATTCTGTTTTACTTCAGATTGTTTTTGGA
OH7B    ATAATTATATCC--ATAGGATACATGTAGTAATTCTGTTTTACTTCAGATTGTTTTTGGA
Tzi8    ATAATTATATCC--ATAGGATACATGTAGTAATTCTGTTTTACTTCAGATTGTTTTTGGA

B73     GGAGAAAACTGTAGG-TTTTACTTCAGATTGTTGAAATGTTCC-ATGTTCATTGG-CCAT
HP301   GGAGAAAACTGTAGG-TTTTACTTCAGATTGTTGAAATGTTCC-ATGTTCATTGG-CCAT
OH7B    GGAGAAAACTGTAGG-TTTTACTTCAGATTGTTGAAATGTTCC-ATGTTCATTGG-CCAT
Tzi8    GGAGAAAACTGTAGGGTTTTACTTCAGATTGTTGAAATGTTCCCATGTTCATTGGGCCAT

B73     GATTAGCATTTTTAAGCATTTCCTGAGTAACAGAATAATTTGGTTTCAATCTCTTTATTC
HP301   GATTAGCATTTTTAAGCATTTCCTGAGTAACAGAATAATTTGGTTTCAATCTCTTTATTC
OH7B    GATTAGCATTTTTAAGCATTTCCTGAGTAACAGAATAATTTGGTTTCAATCTCTTTATTC
Tzi8    GATTAGCATTTTTAAGCATTTCCTGAGTAACAGAATAATTTGGTTTCAATCTCTTTATTC
```

```
B73      TGTTGGTTTGTTATACAACTTCTGTACTGTCCTTGATTATCTCATTTTTTAAGTGCACTG
HP301    TGTTGGTTTGTTATACAACTTCTGTACTGTCCTTGATTATCTCATTTTTTAAGTGCACTG
OH7B     TGTTGGTTTGTTATACAACTTCTGTACTGTCCTTGGTTATCTCATTTTTTAAGTGCACTG
Tzi8     TGTTGGTTTGTTATACAACTTCTGTACTGTCCTTGATTATCTCATTTTTTAAGTGCACTG

B73      CTTCTTTCTTTATTTTGTGCCCTCCAGTTTTCTCATATGTCCACACTTCACTTACATTTT
HP301    CTTCTTTCTTTATTTTGTGCCCTCCAGTTTTCTCATATGTCCACACTTCACTTACATTTT
OH7B     CTTCTTTCTTTATTTTGTGCCCTCCAGTTTTCTCATATGTCCACACTTCACTTACATTTT
Tzi8     CTTCTTTCTTTATTTTGTGCCCTCCAGTTTTCTCATATGTCCACACTTCACTTACATTTT

B73      -ATTTCTTCTCTCTTTGAGGGATAACACAAACCTGCTATCAGCATTGGAAAGAGTGTGAA
HP301    -ATTTCTTCTCTCTTTGAGGGATAACACAAACCTGCTATCAGCATTGGAAAGAGTGTGAA
OH7B     TATTTCTTCTCTCTTTGAGGGATAACACAAACCTGCTATCAGCATTGGAAAGAGTGTGAA
Tzi8     -ATTTCTTCTCTCTT-GAGGGATAACACAAACCTGCTATCAGCATTGGAAAGAGTGTGAA
             *********** ******************                         **
                            ↑ Acceptor iii

B73      TCATTTTCCATTGGTACATAACTTAATTGGGTTACATTTTTGTATGTTTTTCTTAGTATC
HP301    TCATTTTCCATTGGTACATAACTTAATTGGGTTACATTTTTGTATGTTTTTCTTAGTATC
OH7B     TCATTTTCCATTGGTACTTAACTTAATTGGGTTACATTTTTGTATGTTTTTCTTAGTATC
Tzi8     TCATTTTCCATTGGTACATAACTTAATTGGGTTACATTTTTGTATGTTTTTCTTAGTATC
         ***************** **********
                      ↑ Donor iv

B73      AAATTTACACACTGGCGAACACTTCTGGTTATTCTTGTAGTGGGTGATTGAAATATCAGA
HP301    AAATTTACACACTGGCGAACACTTCTGGTTATTCTTGTAGTGGGTGATTGAAATATCAGA
OH7B     AAATTTACACACTGGGGAACACTTCTGGTTATTCTTGTAGTGGGTGATTGAAATATCAGA
Tzi8     AAATTTACACACTGGCGAACACTTCTGGTTATTCTTGTAGTGGGTGATTGAAATATCAGA
                                  ******************************
                                                ↑ Acceptor iv

B73      CAAGCCCAAGCAAAGGGAATCTGAACCAGCATTCAAGGTTTGTGGTGACATTTTCTTAAC
HP301    CAAGCCCAAGCAAAGGGAATCTGAACCAGCATTCAAGGTTTGTGGTGACATTTTCTTAAC
OH7B     CAAGCCCAGGAAAAGGGAATCTGAACCAGCATTCAAGGTTTGTGGTGACATTTTCTTAAC
Tzi8     CAAGCCCAAGCAAAGGGAATCTGAACCAGCATTCAAGGTTTGTGGTGACATTTTCTTAAC
                               ******************************
                                             ↑ Donor v

B73      GCTATTGAGTGCGGCTTTCCTTTTTCGTAATTTTGACAGTGGAAATAAACTCTTGGTTGG
HP301    GCTATTGAGTGCGGCTTTCCTTTTTCGTAATTTTGACAGTGGAAATAAACTCTTGGTTGG
OH7B     GCTATTGAGTGCGGCTTTCCTTTTTCGTAATTTCGACAGTGGAAATAAACTCTTGGTTGG
Tzi8     GCTATTGAGTGCGGCTTTCCTTTTTCGTAATTTTGACAGTGGAAATAAACTCTTGGTTGG

B73      AGATTTCCTGTGTCGATCTTCTATTTTTTTGTTTAACCCTTGTCTAAAAGGGTAGTTATG
HP301    AGATTTCCTGTGTCGATCTTCTATTTTTTTGTTTAACCCTTGTCTAAAAGGGTAGTTATG
OH7B     AGATTTCCTATGTCGATCTTCTATTTTTTTGTTTAACCCTTGTCTAAAAGGGTAGTTATG
Tzi8     AGATTTCCTGTGTCGATCTTCTATTTTTTTGTTTAACCCTTGTCTAAAAGGGTAGTTATG

B73      TTCAATGGCAATGCAATGCCTTTACATCATTTTCTTGAATTCAACTTCCATGATGCCACA
HP301    TTCAATGGCAATGCAATGCCTTTACATCATTTTCTTGAATTCAACTTCCATGATGCCACA
OH7B     TTCAATGGCAATGCAATGCCTTTACATCATTTTCTTGAATTCAACTTCCATGATGCCACA
Tzi8     TTCAATGGCAATGCAATGCCTTTACATCATTTTCTTGAATTCAACTTCCATGATGCCACA

B73      TCTCGAGCACGGAGTAGCGTAGGCTCACACTTAACACATATTTCTGCAGTGGCAAACATG
HP301    TCTCGAGCACGGAGTAGCGTAG-CTCACACTTAACACATATTTCTGCAGTGGCAAACATG
OH7B     TCTCGAGCACGGAGTAGCGTAGGCTCACACTTAACACATATTTCTGCAGTGGCAAACATG
Tzi8     TCTCGAGCACGGAGTAGCGTAGGCTCACACTTAACACATATTTCTGCAGTGGCAAACATG
```

```
B73      CAATCATGTAATGCCATATTGGCAGTTACTAAACACATATTTTTGAAAGGATGTAATATA
HP301    CAATCATGTAATGCCATATTGGCAGTTACTAAACACATATTTTTGAAAGGATGTAATATA
OH7B     CAATCATGTAATGCCATATTGGCAGTTACTAAACACATATTTTTGAAAGGATGTAATATA
Tzi8     CAATCATGTAATGCCATATTGGCAGTTACTAAACACATATTTTTGAAAGGATGTAATATA

B73      CCCAAAGATTTAGTGTTGAATAGGAGCGAATGGAAAACAACTATCCACGTGCCTGAACCT
HP301    CCCAAAGATTTAGTGTTGAATAGGAGCGAATGGAAAACAACTATCCACGTGCCTGAACCT
OH7B     CCCAAAGATTTAGTGTTGAATAGGAACGAATGGAAAACAACTATCCACGTGCCTGAACCT
Tzi8     CCCAAAGATTTAGTGTTGAATAGGAGCGAATGGAAAACAACTATCCACGTGCCTGAACCT

B73      TGATTTGTGGTTTCTGTTGGGTTTCAACTCTAGCCTACATCAACTTGCTTGGGACTAAAA
HP301    TGATTTGTGGTTTCTGTTGGGTTTCAACTCTAGCCTACATCAACTTGCTTGGGACTAAAA
OH7B     TGATTTGTGGTTTCTGTTGGCTTTCAACTCTAGCCTACATCAACTTGCTTGGGACTAAAA
Tzi8     TGATTTGTGGTTTCTGTTGGGTTTCAACTCTAGCCTACATCAACTTGCTTGGGACTAAAA

B73      -GACTTTGTTGTTG-TTGTACA-CATGCAGTTCATTGGAAA---------CGTTCATGGT
HP301    AGACTTTGTTGTTG-TTGTACAACATGCAGTTCATTGGAAAACGTTGAAACGTTCATGGT
OH7B     -GACTTTGTTGTTG-TTGTACA-CATGCAGTTCATTGTAAA---------TGTTCATGGT
Tzi8     -GACTTTGTTGTTGGTTGTACA-CATGCAGTTCATTGGAA----------CGTTCATGGT
                      * ******* ************** **
                                     ⬆ Acceptor v

B73      GATGAGCCTGTTGCAAGAGAGGTTCTTATGCATCTTGCAAATTGGCTGTGTGATAACTAT
HP301    GATGAGCCTGTTGCAAGAGAGGTTCTTATGCATCTTGCAAATTGGCTGTGTGATAACTAT
OH7B     GATGAGCCTGTTGGAAGAGAGGTTCTTATGCATCTTGCAAATTGGCTGTGTGATAACTAT
Tzi8     GATGAGCCTGTTGCAAGAGAGGTTCTTATGCATCTTGCAAATTGGCTGTGTGATAACTAT

B73      CTGAAAGATTCACTGGTAAGCAACATTCCTCTTTGTTACTTGCTCTTTGGTCACCCTGCA
HP301    CTGAAAGATTCACTGGTAAGCAACATTCCTCTTTGTTACTTGCTCTTTGGTCACCCTGCA
OH7B     CTGAAAGATTCACTGGTAAGCA-CATTCCTCTTTGTTACTTGCTCTTTGGTCAC--TGCA
Tzi8     CTGAAAGATTCACTGGTAAGCAACATTCCTCTTTGTTACTTGCTCTTTGGTCACCCTGCA
         ********************** *******
                       ⬆ Donor vi

B73      GTTTACCAATAGCCAATCTCTTGTAATTTTTAGCTTTGCAATAACTACCATTTGTTTTTA
HP301    GTTTACCAATAGCCAATCTCTTGTAATTTTTAGCTTTGCAATAACTACCATTTGTTTTTA
OH7B     GTTTACCAATAGCCAATCTCTTGTAATTTTTAGCTTTGCAATAACTACTTTTTGTTTTTA
Tzi8     GTTTACCAATAGCCAATCTCTTGTAATTTTTAGCTTTGCAATAACTACCATTTGTTTTTA

B73      TTGAGTTGTTGTTCTGATGATATCATACTTTTTCTACAAATACAATAACACGTGAAAGTT
HP301    TTGAGTTGTTGTTCTGATGATATCATACTTTTTCTACAAATACAATAACACGTGAAAGTT
OH7B     TTGAGTTGTTGTTCTGATGATACCATACTTTTGCTACAAATACAATAACACGTGAAAGTT
Tzi8     TTGAGTTGTTGTTCTGATGATATCATACTTTTTCTACAAATACAATAACACGTGAAAGTT

B73      ATTTGAAATATGAAATGAAATTCTAAAGGACGCTGTTTTTACTTAGTTTCCAATATATTG
HP301    ATTTGAAATATGAAATGAAATTCTAAAGGACGCTGTTTTTACTTAGTTTCCAATATATTG
OH7B     ATTTGAAATATGAAATGAAATTCTAAAGGACGTTGTTTTTACTTAGTTTCCAATATATTG
Tzi8     ATTTGAAATATGAAATGAAATTCTAAAGGATGCTGTTTTTACTTAGTTTCCAATATATTG

B73      TTCAACATTAAGATATTGTGTTCCATGTAACTATGATGTATAACACAACATATTTTCATT
HP301    TTCAACATTAAGATATTGTGTTCCATGTAACTATGATGTATAACACAACATATTTTCATT
OH7B     TTCAACATTAAGATACTGTGTTCCATGTAACTATGATGTATAGCACAACATATTTTCATT
Tzi8     TTCAACATTAAGATATTGTGTTCCATGTAACTATGATGTATAACACAACATATTTTCATT
```

```
B73      AACTGTTAAAATGGTTGACTGTATATGGGAAAGGTCAGCAGCTGAACTAATTTTTGTAGG
HP301    AACTGTTAAAATGGTTGACTGTATATGGGAAAGGTCAGCAGCTGAACTAATTTTTGTAGG
OH7B     AACTGTTAAAATGGTTGACTGTATATGGGAAAGGTCAGCAGGTGAACTAATTTTTGTAGG
Tzi8     AACTGTTAAAATGGTTGACTGTATATGGGAAAGGTCAGCAGCTGAACTAATTTTTGTAGG
                                                     ****************
                                                       Acceptor vi ⬆

B73      CAACTCTCATTGTTGAGAACATGCACCTTCATATACTTCCGACAATGAACCCTGATGGGT
HP301    CAACTCTCATTGTTGAGAACATGCACCTTCATATACTTCCGACAATGAACCCTGATGGGT
OH7B     CAACTCTCATTATTGAGAACATGCACCTTCATATA-TTACGACAATGAACCCTGATGGGT
Tzi8     CAACTCTCATTGTTGAGAACATGCACCTTCATATACTTCCGACAATGAACCCTGATGGGT
         *********** **

B73      TTGCTCTTAGATGGCATGGTAATGCAAACAATATTGATCTCAACAGGGATTTTCCTGACC
HP301    TTGCTCTTAGATGGCATGGTAATGCAAACAATATTGATCTCAACAGGGATTTTCCTGACC
OH7B     TTGCTCTTAGATGGCGTGGTAATGCAAACAATATTGTTCTCAACAGGGATTTTCCTGACC
Tzi8     TTGCTCTTAGATGGCATGGTAATGCAAACAATATTGATCTCAACAGGGATTTTCCTGACC
                                                        *************

B73      AA-GTGAGTTACTTCTTTACATTACACCTCTTTTCCTTATGGAAGCTTCTACTTCCCTTT
HP301    AA-GTGAGTTACTTCTTTACATTACACCTCTTTTCCTTATGGAAGCTTCTACTTCCCTTT
OH7B     AAAGTGAGTTACTTCTTTACATTACACCTCTTTTCCTTATGGAAGCTTCTACTTCCCTTT
Tzi8     AA-GTGAGTTACTTCTTTACATTACACCTCTTTTCCTTATGGAAGCTTCTACTTCCCTTT
         ** **************
           ⬆ Donor vii

B73      TTGTACTCATTTTTT-CTTGTAGCCATT-CAATTGACACTAGCCGAAATTGA-AGTTCTT
HP301    TTGTACTCATTTTTT-CTTGTAGCCATT-CAATTGACACTAGCCGAAATTGA-AGTTCTT
OH7B     TTGTACTCATTTTTTTCTTGTAGCCATTTCAATTGACACTAGCTGAAATTGACAGTTCTT
Tzi8     TTGTACTCATTTTTT-CTTGTAGCCATT-CAATTGACACTAGCCGAAATTGA-AGTTCTT

B73      CCCCGTTAACAACGATATTGACTACAGACAGCCTGAAACTAGAGCCATTATGAATTGGGT
HP301    CCCCGTTAACAACGATATTGACTACAGACAGCCTGAAACTAGAGCCATTATGAATTGGGT
OH7B     CCCCGTTAACAACGATATTGACTACAGACAGCCTGAAACTAGAGCCATTATGAATTGGGT
Tzi8     CCCCGTTAACAACGATATTGACTACAGACAGCCTGAAACTAGAGCCATTATGAATTGGGT
                     ******************************
                               ⬆ Donor vii

B73      AAAGCAAGAACACTTCACGACTTCTGCTAGCTTGCATGGGGTAACTGTTCTCACGTACTG
HP301    AAAGCAAGAACACTTCACGACTTCTGCTAGCTTGCATGGGGTAACTGTTCTCACGTACTG
OH7B     AAAGCAAGAACACTTCACGGCTTCTGCTAGCTTGCATGGGGTAACTGTTCTCACGTACTG
Tzi8     AAAGCAAGAACACTTCACGACTTCTGCTAGCTTGCATGGGGTAACTGTTCTCACGTACTG
                                  ******************************
                                            ⬆ Donor viii

B73      TAGCTATAATATCAGTTATCCTGTTGAAATGAGATCCTATAGTTTTCTCTTATTTGATTT
HP301    TAGCTATAATATCAGTTATCCTGTTGAAATGAGATCCTATAGTTTTCTCTTATTTGATTT
OH7B     TAGCTATAATATCAGCTATCCTGTTGAAATGAGATCCTATAGTTTTCTCTTATTTGATTT
Tzi8     TAGCTATAATATCAGTTATCCTGTTGAAATGAGATCCTATAGTTTTCTCTTATTTGATTT
                                                                *****

B73      TGTTCTTCAGGGGGCTCTTGTTGCCAACTATCCATGGGATGGAACTAGAGACACAAGGTG
HP301    TGTTCTTCAGGGG-CTCTTGTTGCCAACTATCCATGGGATGGAACTAGAGACACAAGGTG
OH7B     TGTTCTTCAAGGGGCTCTTGTTGCCAACTATCCATGGGATGGAACTAGAGACACAAGGTG
Tzi8     TGTTCTTCAGGGGGCTCTTGTTGCCAACTATCCATGGGATGGAACTAGAGACACAAGGTG
         ********* *** ***********                 ******************
                   ⬆ Acceptor viii                     Donor ix  ⬆

B73      ATTT-CTTCTTCTACTTAATTTCAAGTT-----------TCTCAATTAATAGTGTTGGGT
HP301    ATTT-CTTCTTCTACTTAATTTCAAGTT-----------TCTCAATTAATAGTGTTGGGT
OH7B     ATTT-CTTCTTCTACTTAATTTCAAGTTAATTTCAAGTTTCTCAATTAATAGTGTTGGGT
Tzi8     ATTTTCTTCTTCTACTTAATTTCAAGTT-----------TCTCAATTAATAGTGTTGGGT
         **** ********
```

```
B73      GTGTTTGGTTGCGGGACGGCCAGGACAAGGATGTCCCCTGGCGTCCTCTCTCGTTCCTCC
HP301    GTGTTTGGTTGCGGGACGGCCAGGACAAGGATGTCCCCTGGCGTCCTCTCTCGTTCCTCC
OH7B     GTGTTTGGTTGCGGGACGGCCAGGATAGGGATGTCCCCTAGTGCCCTCTCTCGTTCCTCC
Tzi8     GTGTTTGGTTGCGGGACGGCCAGGACAAGGATGTCCCCTGGCGTCCTCTCTCGTTCCTCC

B73      AATTTTGAGGGATAACTGGGGACAACACTGGGATAGTCATGTCCCAACTCTTGACCCTGA
HP301    AATTTTGAGGGATAACTGGGGACAACACTGGGATAGTCATGTCCCAACTCTTGACCCTGA
OH7B     AATTTTGAGGGATAACTGGGGACAACACTGGGATAGTCATGTCCCAACTCTTGACCCTGA
Tzi8     AATTTTGAGGGATAACTGGGGACAACACTGGGATAGTCATGTCCCAACTCTTGACCCTGA

B73      ACCAAACAACCTTATTTGAGGGATCGTCCCATCCCGTCCCGTCCTGTCCTATCATTGCAA
HP301    ACCAAACAACCTTATTTGAGGGATCGTCCCATCCCGTCCCGTCCTGTCCTATCATTGCAA
OH7B     ACCAAACAACCTTATTTGAGGGATCGTCACATCCCGTCCTGTCCCGTCCTGTCATTGCAA
Tzi8     ACCAAACAACCTTATTTGAGGGATCGTCCCATCCCGTCCCGTCCTGTCCTATCATTGCAA

B73      CCAAACGCATCCTAAAACTTCTCATGAAGTATTGCCCAAAAACTATCTTCCCATGGTTCT
HP301    CCAAACGCATCCTAAAACTTCTCATGAAGTATTGCCCAAAAACTATCTTCCCATGGTTCT
OH7B     CCAAACGCATCCTAAAACTTCTCATGAAGTATTGCCCAAAAACTATCTTCCCATGGTTCT
Tzi8     CCAAACGCATCCTAAAACTTCTCATGAAGTATTGCCCAAAAACTATCTTCCCATGGTTCT

B73      AATGGTTATTTGAAGTTTGGATTGCAAGTAGGGGCCTTAATATTTCCTAATAAGATGTTT
HP301    AATGGTTATTTGAAGTTTGGATTGCAAGTAGGGGCCTTAATATTTCCTAATAAGATGTTT
OH7B     AATGGTTATTTGAAGTTTGGATTGCAAGTAGGGGCCTTAATATGTCCTAATAAGATGTTT
Tzi8     AATGGTTATTTGAAGTTTGGATTGCAAGTAGGGGCCTTAATATTTCCTAATAAGATGTTT

B73      CCACTTTCCACTGTTAGTTGCATTTTTATTCTTTATATGAAAATGGTTTGTTTTACTATC
HP301    CCACTTTCCACTGTTAGTTGCATTTTTATTCTTTATATGAAAATGGTTTGTTTTACTATC
OH7B     CCACTTTCCACTGTTAGTTGCATTTTTATTCTTTATATGAAAATGGTCTGTTTTACTATC
Tzi8     CCACTTTCCACTGTTAGTTGCATTTTTATTCTTTATATGAAAATGGTTTGTTTTACTATC

B73      ACTGCCCTCTTGACACCTAAACAAAATTTTGACTCATTTTGCTCTCCCAAACTATTTAAT
HP301    ACTGCCCTCTTGACACCTAAACAAAATTTTGACTCATTTTGCTCTCCCAAACTATTTAAT
OH7B     ACTGCCCTCTTGACACCTAAACAAAATTTTGACTCATTTTGCTCTCCCAAACTATTTAAT
Tzi8     ACTGCCCTCTTGACACCTAAACAAAATTTTGACTCATTTTGCTCTCCCAAACTATTTAAT

B73      CTGGGCCAGTTCACCCCCTAATGAAAATATTATATTTCCTTCTCCATGTTCAAATATTGA
HP301    CTGGGCCAGTTCACCCCCTAATGAAAATATTATATTTCCTTCTCCATGTTCAAATATTGA
OH7B     CTGGGCCAGTTCACCCC-TAATGAAAATATTCTATTTCCTTCTCCATATTCAAATACTGA
Tzi8     CTGGGCCAGTTCACCCCCTAATGAAAATATTATATTTCCTTCTCCATGTTCAAATATTGA

B73      TATACTTTTGTATCATAAGGTCTGTTGGCATCTATCATTTTGCAAATTTGCACCAGCAAC
HP301    TATACTTTTGTATCATAAGGTCTGTTGGCATCTATCATTTTGCAAATTTGCACCAGCAAC
OH7B     TATACTTTTGTATCATAAGGTCTGTTGGCATCTATCATTTTGCAAATTTGCACCAGCAAC
Tzi8     TATACTTTTGTATCATAAGGTCTGTTGGCATCTATCATTTTGCAAATTTGCACCAGCAAC
             ******************************
                            ⬆ Acceptor ix
B73      AGAACATGTACATGGCGAAGACAAATCATTAGGTGGCGACTTTTTTCTCACCACGGTAAC
HP301    AGAACATGTACATGGCGAAGACAAATCATTAGGTGGCGACTTTTTTCTCACCACGGTAAC
OH7B     AGAACATGTACATGGCAAAGACAAATCATTAGGTGGTGACTTTTTTCTCACCACGGTAAC
Tzi8     AGAACATGTACATGGCGAAGACAAATCATTAGGTGGCGACTTTTTTCTCACCACGGTAAC
```

```
B73      ACTGAATTTCATTATGGCAGCAACAAAGTTACAATATTCAGGTTTCATTAGGTGGTGTTT
HP301    ACTGAATTTCATTATGGCAGCAACAAAGTTACAATATTCAGGTTTCATTAGGTGGTGTTT
OH7B     ACTGAATTTCATTATGGCAGCAACAAAGTTACAATATTCAGGTTTCATTAGGTGGTGTTT
Tzi8     ACTGAATTTCATTATGGCAGCAACAAAGTTACAATATTCAGGTTTCATTAGGTGGTGTTT

B73      TTTACTAATATTTGAAGAGTTTGAGTTGGGCGACAGGGGGG-TATTGCCAGCAACTGGTT
HP301    TTTACTAATATTTGAAGAGTTTGAGTTGGGCGACAGGGGGG-TATTGCCAGCAACTGGTT
OH7B     CTTACTAATATTTGAAGAGTTTGAGTTGGGCGACAGGGGGGGTATTGCCAGCAACTGGTT
Tzi8     TTTACTAATATTTGAAGAGTTTGAGTTGGGCGACAGGGGGG-TATTGCCAGCAACTGGTT

B73      AATGAACTGTTATGGCTGAAGTGAACCATTAATATAGAGTTTGAGTTATTCTGTGTTGTC
HP301    AATGAACTGTTATGGCTGAAGTGAACCATTAATATAGAGTTTGAGTTATTCTGTGTTGTC
OH7B     AATGAATTGCTATGGCTGAAGTGAACCATTAATGTAGAGTTTGAGTTATTCTGTGTTGTC
Tzi8     AATGAACTGTTATGGCTGAAGTGAACCATTAATATAGAGTTTGAGTTATTCTGTGTTGTC

B73      ACAGACAACCGTATTTACACACACAGTATCTCGCCCGCTCCTCCAAGTCTCCCTCTCTCA
HP301    ACAGACAACCGTATTTACACACACAGTATCTCGCCCGCTCCTCCAAGTCTCCCTCTCTCA
OH7B     ACAGATAACCGTATTTACACACACAGTATCTCGCCTGCTCCTCCAAGTCTCCCTCTCTCA
Tzi8     ACAGACAACCGTATTTACACACACAGTATCTCGCCCGCTCCTCCAAGTCTCCCTCTCTCA

B73      ATATAAGCCTATTTCTGAATTCTATTTGTGGTCGCTTTCTTTG-ATTTGCTGATTGTCAC
HP301    ATATAAGCCTATTTCTGAATTCTATTTGTGGTCGCTTTCTTTG-ATTTGCTGATTGTCAC
OH7B     ATATAAGCCTATTTCTGAATTCTATTTATGGTCGCTTTCTTTGTATTTGCTGATTGTCAC
Tzi8     ATATAAGCCTATTTCTGAATTCTATTTGTGGTCGCTTTCTTTG-ATTTGCTGATTGTCAC

B73      CACTGGTTTTTAGGGCTCAATATGACTTTGTTTCACATTGTAACATCCTCAACAATGATT
HP301    CACTGGTTTTTAGGGCTCAATATGACTTTGTTTCACATTGTAACATCCTCAACAATGATT
OH7B     CACTGGTTTTTAGGGCTCAATATGACTTTGTTTCACATTGTAACATCCTCAACAATGATT
Tzi8     CACTGGTTTTTAGGGCTCAATATGACTTTGTTTCACATTGTAACATCCTCAACAATGATT

B73      TTACTTCTTACAACAAACACTACTACGGATGTCCTGATGATAAGACATTCCAGCACATGG
HP301    TTACTTCTTACAACAAACACTACTACGGATGTCCTGATGATAAGACATTCCAGCACATGG
OH7B     TTACTTCTTACAGCAAACACTACTACAGATGTCCTGGTGATAAGACATTCCGGCACATGG
Tzi8     TTACTTCTTACAACAAACACTACTACGGATGTCCTGATGATAAGACATTCCAGCACATGG

B73      CATCTGTGTATAGTCGGTCTCACTATAACATGTCTTTGAGCAAAGAATTTGAAGGAGGGA
HP301    CATCTGTGTATAGTCGGTCTCACTATAACATGTCTTTGAGCAAAGAATTTGAAGGAGGGA
OH7B     CATCTGTGTATAGTCGGTCTCACTATAACATGTCTTTGAGCAAAGAATTTGAAGGAGGGA
Tzi8     CATCTGTGTATAGTCGGTCTCACTATAACATGTCTTTGAGCAAAGAATTTGAAGGAGGGA

B73      TAACAAATGGAGCATTCTGGTAAACAGACAATAGCAAGTTCATATTTCATAATTCATATG
HP301    TAACAAATGGAGCATTCTGGTAAACAGACAATAGCAAGTTCATATTTCATAATTCATATG
OH7B     TAACAAATGGAGCATTCTGGTAAACAAACAATAGCAAGTTCATATTTCATAATTCATATG
Tzi8     TAACAAATGGAGCATTCTGGTAAACAGACAATAGCAAGTTCATATTTCATAATTCATATG
             ********************** *******
                           ↑ Donor x

B73      AGATTTCCTTTATCTAGGTACTACGTAGGTTGTGTATTTGTTGGCTTTGAATCAAACAAC
HP301    AGATTTCCTTTATCTAGGTACTACGTAGGTTGTGTATTTGTTGGCTTTGAATCAAACAAC
OH7B     AGATTTCCTTTATCTAGGTACTAGGTAGGTTGTGTATTTGTTGGCTTTGAATCAAACAAC
Tzi8     AGATTTCCTTTATCTAGGTACTACGTAGGTTGTGTATTTGTTGGCTTTGAATCAAACAAC
```

```
B73     ATCATTGCTTGTGGTCCAGGTACCCAATATATGGTGGTATGCAAGACTGGAACTATATAC
HP301   ATCATTGCTTGTGGTCCAGGTACCCAATATATGGTGGTATGCAAGACTGGAACTATATAC
OH7B    ATCATCGCTTGTGGTCCAGGTACCCAATATATGGTGGTATGCAGGACTGGAACTATATAC
Tzi8    ATCATTGCTTGTGGTCCAGGTACCCAATATATGGTGGTATGCAAGACTGGAACTATATAC
            * ****************************
                           ⬆ Acceptor x

B73     ATGGAGGCTGCTTTGAGTTAACTCTGGAGATTAGTGACACAAAGTGGCCAAAAGCAGATG
HP301   ATGGAGGCTGCTTTGAGTTAACTCTGGAGATTAGTGACACAAAGTGGCCAAAAGCAGATG
OH7B    ATGGAGGCTGCTTTGAGTTAACTCTGGAGATTAGTGACACAAAGTGGCCAAAAGCAGATG
Tzi8    ATGGAGGCTGCTTTGAGTTAACTCTGGAGATTAGTGACACAAAGTGGCCAAAAGCAGATG
                                                       *************

B73     AGGTACCTTTGCATCGAGTTAGGTTCCACTTCTACTGTTGCAGACCCAGACCATTTTATA
HP301   AGGTACCTTTGCATCGAGTTAGGTTCCACTTCTACTGTTGCAGACCCAGACCATTTTATA
OH7B    AGGTACCTTTGCATCGAGTTAGGTTCCACTTCTACTGTTGCAGACCCAGACCATTTTATA
Tzi8    AGGTACCTTTGCATCGAGTTAGGTTCCACTTCTACTGTTGCAGACCCAGACCATTTTATA
        *****************
          ⬆ Donor xi

B73     ATTATTCTTCTGTAATGTTGTTTATCCTAACATTTTCCTACAGCTTCCTATCATCTGGGA
HP301   ATTATTCTTCTGTAATGTTGTTTATCCTAACATTTTCCTACAGCTTCCTATCATCTGGGA
OH7B    ATTATTCTTCTGTAATGTTGTTTATCCTAACATTTTCCTACAGCTTCCTATCATCTGGGA
Tzi8    ATTATTCTTCTGTAATGTTGTTTATCCTAACATTTTCCTACAGCTTCCTATCATCTGGGA
                                    ******************************
                                               ⬆ Acceptor xi

B73     ACACAGTAGGATGAGTATGCTCAATCTTCTAGCAAGCCTAATAAAGGTAATATGTTTCCA
HP301   ACACAGTAGGATGAGTATGCTCAATCTTCTAGCAAGCCTAATAAAGGTAATATGTTTCCA
OH7B    ACACAATAGGATGAGTATGCTCAATCTTCTAGCAAGCCTAATAAAGGTAATATGTTTCCA
Tzi8    ACACAGTAGGATGAGTATGCTCAATCTTCTAGCAAGCCTAATAAAGGTAATATGTTTCCA
                                       *****************************
                                                      ⬆ Donor xii

B73     CAGTAGTCTCTTTATGCACTGCACAGGGTGATTTTTGCACCAATTGAATGGTTAGTGAAT
HP301   CAGTAGTCTCTTTATGCACTGCACAGGGTGATTTTTGCACCAATTGAATGGTTAGTGAAT
OH7B    CAATAGTCTCTTTATGCACTGCACAAGGTGATTTTTGCACCAATTGAATGGTTAGTGAAT
Tzi8    CAGTAGTCTCTTTATGCACTGCACAGGGTGATTTTTGCACCAATTGAATGGTTAGTGAAT
        *

B73     TGAGGCACTTGGTAGTTGGTTGCATATGTTTAGACTAGGGTCTGGATCATGTACTGTAAC
HP301   TGAGGCACTTGGTAGTTGGTTGCATATGTTTAGACTAGGGTCTGGATCATGTACTGTAAC
OH7B    TGAGGCACTTGGTAGTTGGTTGCATATGTTTAGACTAGGATCTGGATCATGTATTGTAAC
Tzi8    TGAGGCACTTGGTAGTTGGTTGCATATGTTTAGACTAGGGTCTGGATCATGTACTGTAAC

B73     TATTCGATTTTGACGCCTGTAGATTGCAAGAACGGGACTGCTAAGCAAGCTAAGATTTCA
HP301   TATTCGATTTTGACGCCTGTAGATTGCAAGAACGGGACTGCTAAGCAAGCTAAGATTTCA
OH7B    TGTTCGATTTTGACGCCTGCAGATTGCAAGAACAGGGCTGCTAAGTAAGCTAAGATTTCA
Tzi8    TATTCGATTTTGACGCCTGTAGATTGCAAGAACGGGACTGCTAAGCAAGCTAAGATTTCA
               ************ ************* **
                              ⬆ Acceptor xii

B73     ATGGCCTATTCCTTCATACACAGGCCATTGAGCGACGAGGAACGCCGT------------
HP301   ATGGCCTATTCCTTCATACACAGGCCATTGAGCGACGAGGAACGCCGT------------
OH7B    ATGGCCTATTCCTTCATACATAGGCCATTGAGCGACGAGGAACGCCGCATCAGCACTCCC
Tzi8    ATGGCCTATTCCTTCATACACAGGCCATTGAGCGACGAGGAACGCCGT------------

B73     ------GTCACTTGCAATGACCAGAGACGGGAGGTCTTCGTTGCCCAGGACATCGCTAAC
HP301   ------GTCACTTGCAATGACCAGAGACGGGAGGTCTTCGTTGCCCAGGACATCGCTAAC
OH7B    GTTGTGGTCACTTGCAATGACCAGAGACGGGAGGTCTCCGTTGCCCAGAACATCGCTAAC
Tzi8    ------GTCACTTGCAATGACCAGAGACGGGAGGTCTTCGTTGCCCAGGACATCGCTAAC
```

```
B73      AAGGAAATTGACCGCACCGTTGCATTCGACAAGGTAAGATGGATTAGCAAATAATATTGT
HP301    AAGGAAATTGACCGCACAAG-------GGCGAATTCCAGCACACTGGCGGCCGTTACTAG
OH7B     AAGGAAATTGACCGCACCGTTGCATTCGACAAGGTAAGATGGATTAGCAAATAATATTGT
Tzi8     AAGGAAATTGACCGCACAAG-------GGCGAATTCT-GCAGA-TATCCATCAC-ACTGG

B73      TTGACATTTAGGTTTTTATATTTTATATAATACACTATTAGGTGAGTGCCTGTGCGTTGC
HP301    TGG--ATCCGAGCTCG------------GATCCACTAGTAA-CGGCCGCCAGTGTGCTG-
OH7B     TTGACATTTAGGTTTTTATATTTTATATAATACACTATTAGGTGAGTGCCTGTGCGTTGC
Tzi8     CGGCCGCTCGAGCATG------------CATCTAGAGGGCC-CAATTCGCCCTATAGTG-
```

Figure S3 Alignment of *Helitron Hel1-333* sequence between the maize inbred lines. The sequence alignment displays the splice junctions i-xii of *Hel1-33*3 in B73 that are shown in figure 4. The blue and gray shaded sequences flanking the splice sites represent exons and introns, respectively. Conserved flanking sequences between all the inbred lines are indicated by asterisks beneath the highlighted regions, while the dashed lines fill the gaps in alignment. The inbred lines are indicated on the left.
