# Supplementary material for: Differential pre-mRNA Splicing Alters the Transcript Diversity of Helitrons Between the Maize Inbred Lines
Source: G3 (Bethesda). 2015 Jun 12;5(8):1703–11. doi: 10.1534/g3.115.018630 (PMC4528327; doi:10.1534/g3.115.018630)
Supplement: Supporting Information [file supp_g3.115.018630_FigureS1.pdf]

```
B73         TTCTCAAAGGAATTCAGAATTTGGCCCTTCGTTTGAGCAAAATAAATGTGAGGTCATGAT
HP301       TTCTCAAAGGAATTCAGAATTTGGCCCTTCGTTTGAGCAAAATAAATGTGAGGTCATGAT
OH7B        TTCTCAAAGGAATTCAGAATTTGGCCCTTCGTTTGAGCAAAATAAATGTGAGGTCATGAT
Tzi8        TTCTCAAAGGAATTCAGAATTTGGCCCTTCGTTTGAGCAAAATAAATGTGAGGTCATGAT


B73         GATGGTCGGTCTTCCGGCCACTGGCAAGTCAACATGGGCAGAGAAATGGGTCAAGGAGCA
HP301       GATGGTCGGTCTTCCGACCTCTGGCAAGTCAACATGGGCAGAGAAATGGGTCAAGGAGCA
OH7B        GATGGTCGGTCTTCCGACCTCTGGCAAGTCAACATGGGCAGAGAAATGGGTCAAGGAGCA
Tzi8        GATGGTCGGTCTTCCGACCTCTGGCAAGTCAACATGGGCAGAGAAATGGGTCAAGGAGCA


B73         TCAGGAGAAACGTTATATCCTTCTCGGAACTAACCTTGTATTGGAGCAAATGAAGGTTGG
HP301       TCAGGAGAAACGTTATATCCTTCTCGGAACTAACCTTGTATTGGAGCAAATGAAGGTTGG
OH7B        TCAGGAGAAACGTTATATCCTTCTCGGAACTAACCTTGTATTGGAGCAAATGAAGGTTGG
Tzi8        TCAGGAGAAACGTTATATCCTTCTCGGAACTAACCTTGTATTGGAGCAAATGAAGGTTGG


B73         TTTCCTTCTGGTGGCAATTGTATTAGGGAGTTCCCTTAATCCTGCAACCTTT-TTTTCAA
HP301       TTTCCTTCTAGTGGCAATTGTATTAGGGAGTTCCCTTAATCCTGCAACCTTTTTTTTCAA
OH7B        TTTCCTTCTAGTGGCAATTGTATTAGGGAGTTCCCTTAATCCTGCAACCTTT-TTTTCAA
Tzi8        TTTCCTTCTAGTGGCAATTGTATTAGGGAGTTCCCTTAATCCTGCAACCTTT-TTTTCAA


B73         TATATAACTAATACCTTGATCATTGCAGGTGCCAGGATTATTGCGTAAGAATAACTATGG
HP301       TATATAACTAATACCTTGATCATTGCAGGTGCCAGGATTATTGCGTAAGAATAACTATGG
OH7B        TATATAACTAATACCTTGATCATTGCAGGTGCCAGGATTATTGCGTAAGAATAACTATGG
Tzi8        TATATAACTAATACCTTGATCATTGCAGGTGCCAGGATTATTGCGTAAGAATAACTATGG


B73         TGAGCGTTTTGAACGACTGATGGATTGTGCTACATGGATTTTCAACAAACTGCTGACTAG
HP301       TGAGCGTTTTGAACGACTGATGGATTGTGCTACATGGATTTTCAACAAACTGCTGACTAG
OH7B        TGAGCGTTTTGAACGACTGATGGATTGTGCTACATGGATTTTCAACAAACTGCTGACTAG
Tzi8        TGAGCGTTTTGAACGACTGATGGATTGTGCTACATGGATTTTCAACAAACTGCTGACTAG


B73         GGCTGCAAATACCCCTCGCAACTTCATTATCGATCAAACGAATGTTTACAAGAATGCTCG
HP301       GGCTGCAAATACCCCTCGCAACTTCATTATCGATCAAACGAATGTTTACAAGAATGCTCG
OH7B        GGCTGCAAATACCCCTCGCAACTTCATTATCGATCAAACGAATGTTTACAAGAATGCTCG
Tzi8        GGCTGCAAATACCCCTCGCAACTTCATTATCGATCAAACGAATGTTTACAAGAATGCTCG


B73         CATTCGCAAGTTGAGGCCATTTGCTAACTACCGCAAGGTAAGCTGTAAAAGAGAAAAAGG
HP301       CATTCGCAAGTTGAGGCCATTTGCTAACTACCGCAACGTAAGCTGCAAAAGAGAAAAAGG
OH7B        CATTCGCAAGTTGAGGCCATTTGCTAACTACCGCAACGTAAGCTGCAAAAGAGAAAAAGG
Tzi8        CATTCGCAAGTTGAGGCCATTTGCTAACTACCGCAACGTAAGCTGCAAAAGAGAAAAAGG
                                  ************** ******** ******
                                                ↑ Donor i & ii

B73         AAATGATAGGTTTCCTGTATGGTAGCACTGGATTGGTTCATAAATACTCCCATATGCAAC
HP301       AAATGATAGGTTTCCTGTATGGTAGCACTGGATTGGTTCATAAATACTCCCATATGCAAC
OH7B        AAATGATAGGTTTCCTGTATGGTAGCACTGGATTGGTTCATAAATACTCCCATATGCAAC
Tzi8        AAATGATAGGTTTCCTGTATGGTAGCACTGGATTGGTTCATAAATACTCCCATATGCAAC
                                                           *************

B73         AGACTGATGTGGTCATGTTCCCAACTCCAAGTGAACTGAAATCCAGGGCAGCGAAGCGTT
HP301       AGACTGTTGTGGTCATGTTCCCAACTCCAAGTGAACTGAAATCCAGGGCAGCGAAGCTTT
OH7B        AGACTGTTGTGGTCATGTTCCCAACTCCAAGTGAACTGAAATCCAGGGCAGCGAAGCTTT
Tzi8        AGACTGTTGTGGTCATGTTCCCAACTCCAAGTGAACTGAAATCCAGGGCAGCGAAGCTTT
            ****** **********
              ↑ Acceptor ii
```

```
B73        TCAATGAGATGGGAAAGGAAATACCAGCTGAAGCAGTTAACGAAATGACAGGTACCTGGA
HP301      TCAATGAGATGGGAAAGGAAATACCAGCTGAAGCAGTTAACGAAATGACAGGTACCTGGA
OH7B       TCAATGAGATGGGAAAGGAAATACCAGCTGAAGCAGTTAACGAAATGACAGGTACCTGGA
Tzi8       TCAATGAGATGGGAAAGGAAATACCAGCTGAAGCAGTTAACGAAATGACAGGTACCTGGA
                                                ************************
                                                              ↑ Donor iii

B73        CATACACACATTATCATTTAACATGTTCGCTGTCTAGCTCCTTTTTTTCCCATTATCTAT
HP301      CATACACACATTATCATTTAACATGTTCGCTGCCTAGCTCCTTT--TTCCCATTATCTAT
OH7B       CATACACACATTATCATTTAACATGTTCGCTGCCTAGCTCCTTT---TCCCATTATCTAT
Tzi8       CATACACACATTATCATTTAACATGTTCGCTGCCTAGCTCCTTT---TCCCATTATCTAT
           ******

B73        ACATCCATTACTTACCAGTTACCTTAAATTATCTTTTTCAGCCAATTTTGTCCTACCACT
HP301      ACATCCATTACTTACCAGTTACCTTAAATTATCTTTTTCAGCCAATTTTGTCCTACCACT
OH7B       ACATCCATTACTTACCAGTTACCTTAAATTATCTTTTTCGGCCAATTTTGTCCTACCACT
Tzi8       ACATCCATTACTTACCAGTTACCTTAAATTATCTTTTTCGGCCAATTTTGTCCTACCACT
                                     ************* ****************
                                                ↑ Acceptor iii

B73        CTCGAAGGATATGCCTCATTCGAAGGAGCCTTTTGATGAGGTATACTCAAATGTTGATAT
HP301      CTCGAAGGATATGCCTCATTCGAAGGAGCCTTTTGATGAGGTATACTCAAATGTTGATAT
OH7B       CTCGAAGGATATGCCTCATTCGAAGGAGCCTTTTGATGAGGTATACTCAAATGTTGATAT
Tzi8       CTCGAAGGATATGCCTCATTCGAAGGAGCCTTTTGATGAGGTATACTCAAATGTTGATAT
                                    ******************************
                                               ↑ Donor iv

B73        TATTGTACATCTTCTGACTAGCGTCAAATGATTACTTAGCTCATTGAGCTTTAAAAACAT
HP301      TATTGTACATCTTCTGACTAGCGTCAAATGATTACTTAGCTCATTGAGCTTAAAAAACAT
OH7B       TATTGTACATCTTCTGACTAGCGTCAAATGATTACTTAGCTCATTGAGCTTAAAAAACAT
Tzi8       TATTGTACATCTTCTGACTAGCGTCAAATGATTACTTAGCTCATTGAGCTTAAAAAACAT
                                                               ********

B73        TTTCAGGTAATCTTCACAGAGCTTTCTAGGGATGAGGCTCAGAGAACCCTAGATGATATG
HP301      TTTTAGGTAATCTTCACAGAGCTTTCTAGGGATGAGGCTCAGAGAACCCTAGATGATATG
OH7B       TTTCAGGTAATCTTCACAGAGCTTTCTAGGGATGAGGCTCAGAGAACCCTAGATGATATG
Tzi8       TTTCAGGTAATCTTCACAGAGCTTTCTAGGGATGAGGCTCAGAGAACCCTAGATGATATG
           *** *****************
                ↑ Acceptor iv

B73        CAGCGTGTGTTGCCAAGAAATGTGACCCCAAGCTATGGAAACTCTGGCAACCAAAAACAT
HP301      CAGCGTGTGTTGCCAAGAAATGTGACCCCAAGCTATGGAAACTCTGGCAACCAAAAACAT
OH7B       CAGCGTGTGTTGCCAAGAAATGTGACCCCAAGCTATGGAAACTCTGGCAACCAAAAACAT
Tzi8       CAGCGTGTGTTGCCAAGAAATGTGACCCCAAGCTATGGAAACTCTGGCAACCAAAAACAT
                                                        ***************

B73        GTAATGGTGCCTTTATTGTTATTTTTGAAACAGTGCCTTGTATGTTTTATGTCATCATGG
HP301      GTAATGGTGCCTTTATTGTTATTTTTGAAACAGTGCCTTGTATGTTTTATGTCATCATGG
OH7B       GTAATGGTGCCTTTATTGTTATTTTTGAAACAGTGCTTTGTATGTTTTATGTCATCATGG
Tzi8       GTAATGGTGCCTTTATTGTTATTTTTGAAACAGTGCCTTGTATGTTTTATGTCATCATGG
           ***************
          ↑ Donor v

B73        ATCAGATTAGTGTAAATATGCAACGCTATACAACAATGTTCTGATGTTCTGGGACTGC--
HP301      ATCAGATTAGTGTAAATATGCAACGCTATACAACAATGTTCTGATGTTCTGGTGCTTACA
OH7B       ATCAGATTAGTGTAAATATGCAACGCTATACAACAATGTTCTGATGTTCTGGTACTTACA
Tzi8       ATCAGATTAGTGTAAATATGCAACGCTATACAACAATGTTCTGATGTTCTGGTACTTACA

B73        ---------------------------TGCTCCACTTGATCCTGAAGCCACTT-TGCAAA
HP301      GTATAGTTCAACATATGCTGGGACTGCTGCTCCACTTGATCCTGAAGCCACTTGTGCAAA
OH7B       GTATAGTTCAACATATGCTGGGACTGCTGCTCCACTTGATCCTGAAGCCACTTGTGCAAA
Tzi8       GTATAGTTCAACATATGCTGGGACTGCTGCTCCACTTGATCCTGAAGCCACTTGTGCAAA
                                                             *** ******
```

```
B73             TCAAGGTGCACAGTTTTCAAACATCTGGTGTTATTTTCCTATTTATTTAGTGAGACATTT
HP301           TCAAGGTGCACAGTTTTCAAACATCTGGTGTTATTTTCCTATTTATTTAGTGAGACATTT
OH7B            TCAAGGTGCACAGTTTTCAAACATCTGGTGTTATTTTCCTATTTATTTAGTGAGACATTT
Tzi8            TCAAGGTGCACAGTTTTCAAACATCTGGTGTTATTTTCCTATTTATTTAGTGAGACATTT
                ********************
                     ↑ Donor vi

B73             GGAGTTCATTTTGTCTGAATTAAACAACAAGTTTCAGTTGAGGTTAACTGAAATACGTGA
HP301           GGAGTTCATTTTGTCTGAATTAAATAACAAGTTTCAGTTGAGGTTAACTGAAATATGTGA
OH7B            GGAGTTCATTTTGTCTGAATTAAATAACAAGTTTCAGTTGAGGTTAACTGAAATATGTGA
Tzi8            GGAGTTCATTTTGTCTGAATTAAATAACAAGTTTCAGTTGAGGTTAACTGAAATATGTGA

B73             TTTTGTATAAACTGAACCAACGTAAACCAATTTTATTAGGCTACTTAGTAGCAGGGATAT
HP301           TTTTGTATAAACTGAACCAACGTAAACCAATTTTATTAGGCTACTTAGTAGCAGGGATAT
OH7B            TTTTGTATAAACTGAACCAATGTAAACCAATTTTATTAGGCTACTTAGTAGCAGGGATAT
Tzi8            TTTTGTATAAACTGAACCAATGTAAACCAATTTTATTAGGCTACTTAGTAGCAGGGATAT

B73             AAATTAATATAAAGAATGGAAGTTGTTAACTTACATAAACTACTGTGTTGAATTCATGTT
HP301           AAATTAATATAAAGAATGGAAGTTGTTAACTTGCATAAACTACTGTGTTGAAATCATGTT
OH7B            AAATTAATATAAAGAATGGAAGTTGTTAACTTACATAAACTACTGTGTTGAATTCATGTT
Tzi8            AAATTAATATAAAGAATGGAAGTTGTTAACTTACATAAACTACTGTGTTGAATTCATGTT

B73             CATTTTAGCCTTGTCTGCACCTGTTAACTATCCATTTGTCTATACAACTAAGTGCAGTTC
HP301           CATTTTAGCCTTGTCTGCACCTGTTAACTACCCATTTGTCTATACAACTAAGTGCAGTTC
OH7B            CATTTTAGCCTTGTCTGCACCTGTTAACTACCCATTTGTCTATACAACTAAGTGCAGTTC
Tzi8            CATTTTAGCCTTGTCTGCACCTGTTAACTACCCATTTGTCTATACAACTAAGTGCAGTTC

B73             ATAGTCCAAACTAAGGTATCACCATTTTGTCTTAATTATTGCATACAAAACTAATGAAAC
HP301           ATAGTCCGAACTAATGTATCACCATTTTGTCTTAATTATTGCATACAAAACTAATGAAAC
OH7B            ATAGTCCGAACTAATGTATCACCATTTTGTCTTAATTATTGCATACAAAACTAATGAAAC
Tzi8            ATAGTCCGAACTAATGTATCACCATTTTGTCTTAATTATTGCATACAAAACTAATGAAAC

B73             ATGATTATGTTTCTGCATTTTAACGAGTGAGTAGTCCCATAAAATATCAAGAACGACATC
HP301           ATGATTATATTTCTGCATTTTAACGAGCGAGTAGTCCCATAAAATATCAAGAACGACATC
OH7B            ATGATTATGTTTCTGCATTTTAACGAGTGAGTAGTCCCATAAAATATCAAGAACGACATC
Tzi8            ATGATTATGTTTCTGCATTTTAACGAGTGAGTAGTCCCATAAAATATCAAGAACGACATC

B73             CAAAACTAATCCG-TTTTTTTCACGAGGTTTGCTGACATAAGTGGCTTTTGTTTTTGTAG
HP301           CAAAACTAATCCCGTTTTTTTTCACGTGGTTTGCTGACATAAGTGGCTTTTGTTTTTGTAG
OH7B            CAAAACTAAC-CCGTTTTTTTTCACGTGGTTTGCTGACATAAGTGGCTTTTGTTTTTGTAG
Tzi8            CAAAACTAAC-CCGTTTTTTTTCACGTGGTTTGCTGACATAAGTGGCTTTTGTTTTTGTAG

B73             TAGCCAGTTGTCCTTTCTGAACCCTCTCATGCATTGAACCCTCTCATCTGTTGTTATGTT
HP301           TAGCCAGTTGTCCTTTCTGAACCCTCTCATGCATGGAACCCTCTCATCTGTTGTTATGTT
OH7B            TAGCCAGTTGTCCTTTCTGAACCCTCTCATGCATGGAACCCTCTCATCTGTTGTTATGTT
Tzi8            TAGCCAGTTGTCCTTTCTGAACCCTCTCATGCATGGAACCCTCTCATCTGTTGTTATGTT
                                                                       *****

B73             TGTACAACAGTGTTGTACGAGTGCCCGCGACAGGAGCTTGTACGCCAGTGTGTTTTCTTC
HP301           TGTACAACAGTGTTGTACGAGTGCCTGCGACAGGAGCTTGTACGCTAGTGTGTTTTCTTC
OH7B            TGTACAACAGTGTTGTACGAGTGCCTGCGACAGGAGCTTGTACGCTAGTGTGTTTTCTTC
Tzi8            TGTACAACAGTGTTGTACGAGTGCCTGCGACAGGAGCTTGTACGCTAGTGTGTTTTCTTC
                *************************
                          ↑ Acceptor v & vi
```

```
B73           CGGCTGATTGTTTTGCTCTGTGAAGGTTTGAATTCTCGAACATAGTATAGATCAATCATT
HP301         CGGCTGATTGTTTTGCTCTGTGAAGGTTTGAATTCTCGAACATAGTATAGATCAATCATT
OH7B          CGGCTGATTGTTTTGCTCTGTGAAGGTTTGAATTCTCGAACATAGTATAGATCAATCATT
Tzi8          CGGCTGATTGTTTTGCTCTGTGAAGGTTTGAATTCTCGAACATAGTATAGATCAATCATT
                       *******************************
                                       ↑ Donor vii

B73           CAATTAGATGCATTATGTTGGCATCAATATCCAGTGATCTGAGTAGCTTGTACAAAACAT
HP301         CAATTAGATGCATTAGGTTGGCATTAATATCCAGTGATCTGAGTAGCTTGTACAAAACAT
OH7B          CAATTAGATGCATTAGGTTGGCATTAATATCCAGTGATCTGAGTAGCTTGTACAAAACAT
Tzi8          CAATTAGATGCATTAGGTTGGCATTAATATCCAGTGATCTGAGTAGCTTGTACAAAACAT

B73           TATGCCAACCTAATGATAAAACTGTTTTGAATGATTGATACTCAATATCCAGTGAGCTGC
HP301         TATGCCAACCTAATGATAAAACTGTTTTGAATGATTGATACTCAATATCCAGTGAGCTGC
OH7B          TATGCCAACCTAATGATAAAACTGTTTTGAATGATTGATACTCAATATCCAGTGAGCTGC
Tzi8          TATGCCAACCTAATGATAAAACTGTTTTGAATGATTGATACTCAATATCCAGTGAGCTGC

B73           TATAACACTTATACGCATTTTCACTCTTTTGATCCAAGAATAAAATTGCGTATATATTCT
HP301         TATAACATTTATACGCATTTTCACTCTTTTGATCCAAGAATAAAATTGCGTATATATTCT
OH7B          TATAACATTTATACGCATTTTCACTCTTTTGATCCAAGAATAAAATTGCGTATATATTGT
Tzi8          TATAACATTTATACGCATTTTCACTCTTTTGATCCAAGAATAAAATTGCGTATATATTGT
                                                                       * *

B73           GGATCAAAAGAGTGAAACTGTGTATAAGTGCTATAACAGCTTGTGTAGTTCTCATGAGGG
HP301         GGATCAAAAGAGTGAAACTGTGTATAAGTGCTATAATAGCTTGTGTAGTTCTCATGAGGG
OH7B          GGATCAAAAGAGTGAAACTGTGTATAAGTGCTATAATAGCTTGTGTAGTTCTCATGAGGG
Tzi8          GGATCAAAAGAGTGAAACTGTGTATAAGTGCTATAATAGCTTGTGTAGTTCTCATGAGGG
              ***************************
                          ↑ Acceptor i

B73           AATTTGACCCGGGGAACATTGTCTTTTATATTTTTGTTCGGCTGCAGTCGTTGGTGCCAC
HP301         AATTTGACCCGGGGAACATTGTCTTTTATATTTTTGTTCGGCTGCAGTCGTTGGTGCCAC
OH7B          AATTTGACCCGGGGAACATTGTCTTTTATATTTTTGTTTGGCTGCAGTCGTTGGTGCCAC
Tzi8          AATTTGACCCGGGGAACATTGTCTTTTATATTTTTGTTCGGCTGCAGTCGTTGGTGCCAC
                                              ****** *********************
                                                              ↑ Acceptor vii

B73           CATCACACTGCTAGCGTGCAGAAACTCCTCTGTTGATTTAATCCTATCTCCATCAGTGAT
HP301         CATCACACTGCTAGCGTGCAGAAACTCCTCTGTTGATTTAATCCTATCTCCATCAGTGAT
OH7B          CATCACACTGCTAGCGTGCAGAAACTCCTCTGTTGATTTAATCCTATCTCCATCAGTGAT
Tzi8          CATCACACTGCTAGCGTGCAGAAACTCCTCTGTTGATTTAATCCTATCTCCATCAGTGAT
              **

B73           ATAAAGGTACAAGTTGAAGTAAAAGACATGGATATGATGCAGCAAGTCAAGTCTAACAAT
HP301         ATAAAGGTACAAGTTGAAGTAAAAGACATGGATATGATGTAGCAAGTCGAGTCTAACGAT
OH7B          ATAAAGGTACAAGTTGAAGTAAAAGACATGGATATGATGTAGCAAGTCGAGTCTAACGAT
Tzi8          ATAAAGGTACAAGTTGAAGTAAAAGACATGGATATGATGTAGCAAGTCGAGTCTAACGAT
```

Figure S1 Sequence Alignment of *Helitron Hel1-331* between the lines of maize. The sequence alignment exhibits the splice site junctions of various alternatively spliced transcript isoforms (i-vii) of *Hel1-331* in inbred B73 that are shown in figure 2. The arrows point to the donor and acceptor splice sites, and the blue and gray shaded sequence flanking the splice sites represent exons and introns, respectively. Asterisks beneath the highlighted regions indicate conserved flanking sequences between all the inbred lines. The names of the inbred lines are indicated on the left.
