# Supplementary material for: Differential pre-mRNA Splicing Alters the Transcript Diversity of Helitrons Between the Maize Inbred Lines
Source: G3 (Bethesda). 2015 Jun 12;5(8):1703–11. doi: 10.1534/g3.115.018630 (PMC4528327; doi:10.1534/g3.115.018630)
Supplement: Supporting Information [file supp_g3.115.018630_FigureS2.pdf]

```
B73       TCCAGCGTCCTCCACCCCGAATCGCCGCCCCGGCTCTCTCCATGGAAGGCGCGTCTGCGA
HP301     TCCAGCGTCCTCCACCCCGAATCGCCGCCCCGGCTCTCTCCATGGAAGGCGCGTCTGCGA
OH7B      TCCAGCGTCCTCCGCCCCGGATCGTTGCC--CCAGATCTCCATGGAAGGCGTGTCTGTGA
Tzi8      TCCAGCGTCCTCCGCCCCGGATCGTTGCC--CCAGATCTCCATGGAAGGCGTGTCTGTGA

B73       CAGCCCGATCTCCGTGGAAGGCGTGTCTTCCTCCCCAGATCGTCGTCCCGGCTCTCTCCA
HP301     CAGCCCGATCTCCGTGGAAGGCGTGTCCTCCTCCCCAGATCGTCGTCCCGGCTCTCTCCA
OH7B      CAGCCCGATCTCTGTGGAAGGCGCGTCCTCCTCCCCAGATCGTCGTCCCGGCTCTCTCCA
Tzi8      CAGCCCGATCTCTGTGGAAGGCGCGTCCTCCTCCCCAGATCGTCGTCCCGGCTCTCTCCA

B73       CGCCCGTGACAGCCTGCTCTCTATGGAAGGTGAGTCTGCGATGCTCACGTGGATTGGGTA
HP301     CGCCCGTGACAGCCTGCTCTCTATGGAAGGTGAGTCTGCGATGCTCACGTGGATTGGGTA
OH7B      CGCTCGTGACAGCCTGCTCTCTATGGAAGGTGAGTCCGCGATGCTCACGTGGATTGGGTA
Tzi8      CGCTCGTGACAGCCTGCTCTCTATGGAAGGTGAGTCCGCGATGCTCACGTGGATTGGGTA
                        ********************** *******
                                      ↑ Donor iii

B73       CCCCGCCCCCGCTCTCTCCATGGAAGGCGTGTCTCCGATAGCCGCATCCTCCCTTGCGAA
HP301     CCCCGCCCCCGCTCTCTCCATGGAAGGCGTGTCTCCGATAGCCGCATCCTCCCTTGCGAA
OH7B      CCCCGCCCCCGCTCTCTCCATGGAAAGTGTGTCTCCGATAGACGCATCCTCCCTTGCGAA
Tzi8      CCCCGCCCCCGCTCTCTCCATGGAAAGTGTGTCTCCGATAGACGCATCCTCCCTTGCGAA

B73       CCGCGTGCGTCATGGCAACACGGGCGAGGCGGTGGGGAGTCTGGTGGACGCGCATCACGG
HP301     CCGCGTGCGTCATGGCAACACGGGCGAGGCGGTGGGGAGTCTGGTGGACGCGCATCACGG
OH7B      CCGCGCACGTCATGGCAACACGGGCGAGGCAGTGGGGAGTCTGGCGGACGCGCATCACGG
Tzi8      CCGCGCACGTCATGGCAACACGGGCGAGGCAGTGGGGAGTCTGGCGGACGCGCATCACGG

B73       TGAGACGAACGGGGGCAAGGGCAAGGGATATATTTTCCATCCGGGGCGGATGTGGAGAGG
HP301     TGAGACGAACGGGGGCAAGGGCAAGGGATATATTTTCCATCCGGGGCGGATGTGGAGAGG
OH7B      TGAGGCGAACGGGGGCAAGGGCAAGGGATATATTTTCCATCCGGGGCGGATGTGGAGAGG
Tzi8      TGAGGCGAACGGGGGCAAGGGCAAGGGATATATTTTCCATCCGGGGCGGATGTGGAGAGG

B73       TTCTTCAAACTCTGTGACCCTGGTGAGTGCGCTCGATCTCTGGT----------------
HP301     TTCTTCAAACTCTGTGACCCTGGTGAGTGCGCTCGATCTCTGGT----------------
OH7B      TTCTTCAAACTCTGTGACCCTGGTGAGTGCGCTCGATCTCTTTGGTTCCAGGATGTGGAG
Tzi8      TTCTTCAAACTCTGTGACCCTGGTGAGTGCGCTCGATCTCTTTGGTTCCAGGATGTGGAG
                 ******************************
                              ↑ Donor i, iv§, v

B73       ---------------CTTCCGTGGTTTTTCTGCTTGGGTTTCGTTGCTTTGGTGGGGGAT
HP301     ---------------CTTCCGTGGTTTTTCTGCTTGGGTTTCGTTGCTTTGGTGGGGGAT
OH7B      TGATAGTCGAGGAGGCTTCCGTGGTTTTTCTGCTTGGGTTTCGTTGCTTTGGTGGGGGAT
Tzi8      TGATAGTCGAGGAGGCTTCCGTGGTTTTTCTGCTTGGGTTTCGTTGCTTTGGTGGGGGAT

B73       TGGATCATTGGAAGCTAAATTTGGAGAAATCTCCCTTTTCTCTGTCTCCTCTACCCTCAC
HP301     TGGATCATTGGAAGCTAAATTTGGAGAAATCTCCCTTTTCTCTGTCTCCTCTACCCTCAC
OH7B      TGGATCGTTGGAAGCTAAATTTAGAGAAATTT-CCCTTTCTCTGTCTCCTCTACCCTCAC
Tzi8      TGGATCGTTGGAAGCTAAATTTAGAGAAATTT-CCCTTTCTCTGTCTCCTCTACCCTCAC
                                                                     *

B73       TCGACATGCTTCAGATTCGATTATTGGTGGCCTTACAGCTTTCGTTAATGCTCTGCTTGT
HP301     TCGACATGCTTCAGATTCGATTATTGGTGGCCTTACAGCTTTCGTTAATGCTCTGCTTGT
OH7B      TCGACATGCTTCAGATTCGATTATTGGTGGCCTTGCAGCTTTCGTTAATGCTCTGCTTGT
Tzi8      TCGACATGCTTCGGATTCGATTATTGGTGGCCTTGCAGCTTTCGTTAATGCTCTGCTTGT
          ************ ***************  **** ***********************
                        ↑ Acceptor v                  ↑ Donor ii
```

```
B73       GCTTGATTGTAGCTGTTCGAATTTGGTGAGAGATTGGACCCCGTGATGACAGTTTTATGT
HP301     GCTTGATTGTAGCTGTTCGAATTTGGTGAGAGATTGGACCCCGTGATGACAGTTTTATGT
OH7B      GCTTGATTGTAGCTGTTCGAATTTGGTGAGAGATTGGACCCCGTGATGACAGTTTTATGT
Tzi8      GCTTGATTGTAGCTGTTCGAATTTGGTGAGAGATTGGACCCCGTGATGACAGTTTTATGT
                    ******************************
                                Donor vi

B73       TGGTTGGAACTGTGGAACCAGTGGCTGACATATAATTTTTTTTGCTATTAGGTTGTTACT
HP301     TGGTTGGAACTGTGGAACCAGTGGCTGACATATAATTTTTTTTGCTATTAGGTTGTTACC
OH7B      TGGTTGGAACTATGGAACCAGTGGCTGACATATAATTTTTTTTGCTATTAGGTTGTTACT
Tzi8      TGGTTGGAACTATGGAACCAGTGGCTGACATATAATTTTTTTTGCTAGTAGGTTGTTACT
                                             ************ ***********
                                                           Acceptor vi

B73       TGTTAGCGCTATGGGGTTGCTATCAATCTTGTATTGACAGCTTGCTTGCAGAAAATCACT
HP301     TGTTAGCGCTATGGGGTTGCTATCAATCTTGTATTGACAGCTTGCTTGCAGAAAATCACT
OH7B      TGTTAGCGCTATGGGGTTCCCATCAATCTTGTATTGACAGCTTGCTTGCAGAAAATCGCT
Tzi8      TGTTAGCGCTATGGGGTTCCCATCAATCTTGTATTGACAGCTTGCTTGCAGAAAATCGCT
          ******

B73       AGATATTTTTGGCTGATCGGCGCCGTGGAATTTCGATTGTGTACTGGGTGCTTGGTTTCT
HP301     AGATATTTTTGGCTGATCGGCGCCGTGGAATTTCGATTGTGTACTGGGTGCTTGGTTTCT
OH7B      AGATATTTTTGGCTGATCGGTGCCGTGGAAAATGGACTGTGTACTGGGTGCTTGGTTTCT
Tzi8      AGATATTTTTGGCTGATCGGTGCCGTGGAAAATGGACTGTGTACTGGGTGCTTGGTTTCT

B73       ATATGACTAGGCAGTTTGCTCTTCCATCTATCGCACAGCGCCACATCTTGGCTGGTCTGG
HP301     ATATGACTAGGCAGTTTGCTCTTCCATCTATCGCACAGCGCCACATCTTGGCTGGTCTGG
OH7B      ATATGACTAGGCAGTTTGCTCTTCCATCTATCGCACAGCGCCACATCTTGGCTGGTCTGG
Tzi8      ATATGACTAGGCAGTTTGCTCTTCCATCTATCGCACAGCGCCACATCTTGGCTGGTCTGG

B73       CATCAGCAGTTGACGGTCTGAGTGCACCATATGAAAGAGCATCATGTATTCATGAAAGAC
HP301     CATCAGCAGTTGACGGTCTGAGTGCACCATATGAAAGAGCATCATGTATTCATGAAAGAC
OH7B      CATCAACAGTTGACGGTCTGAGTGCACCATATGAAAGAGCATCACATATTCATGAAAGAC
Tzi8      CATCAACAGTTGACGGTCTGAGTGCACCATATGAAAGAGCATCACGTATTCATGAAAGAC

B73       ATGTAGTGAACTGGCTGTGGGCTGCAGGCTGTCATCCGTTTGGACCATTCTCGAACTCCT
HP301     ATGTAGTGAACTGGCTGTGGGCTGCAGGCTGTCATCCGTTTGGACCATTCTCGAACTCCT
OH7B      ATGTAGTGAACTGGCTGTGGGCTGCAGGCTGTCATCCGTTTGGACCATTCTCGAACTCCT
Tzi8      ATGTAGTGAACTGGCTGTGGGCTGCAGGCTGTCATCCGTTTGGACCATTCTCGAACTCCT

B73       CCCAGATCAGTCAAATTCTTTAGGATGTAGCACTGGTAATGTTATCAACAACATTT-TTT
HP301     CCCAGATCAGTCAAATTCTTTAGGATGTAGCACTGGTAATGTTATCAACAACATTT-TTT
OH7B      CCCAGATCAGTCAAATTCTTCAGGATGTAGCACTGGTAATGTTATCAACAACATTTTTTT
Tzi8      CCCAGATCAGTCAAATTCTTCAGGATGTAGCACTGGTAATGTTATCAACAACATTTTTTT
                               *****************************
                                          Donor vii

B73       TATTTCTATGATATGTACAATGAGTAGGCAGGGAGCGAGGGAGGTCAATGCTCCTATGTC
HP301     TATTTCTATGATATGTACAATGAGTAGGCAGGGAGCGAGGGAGGTCAATGCTCCTATGTC
OH7B      TAATTCTATGATATGTACAATGAGTAGGCAGGGAGCGAGGGAGGTCAATGCTCCTATGTC
Tzi8      TATTTCTATGATATGTACAATGAGTAGGCAGGGAGCGAGGGAGGTCAATGCTCCTATGTC

B73       GAAGTGCAGCTTAAAGTAGAAAATCTGTTCATGCTTCCAATGGTTTGCACAGTGCATAGG
HP301     GAAGTGCAGCTTAAAGTAGAAAATCTGTTCATGCTTCCAATGGTTTGCACAGTGCATAGG
OH7B      GAAGTGTAGCTTAAAGTAGAAAATCTGTTCATGCTTCCAATGGTTTGCACAGTGCATAGG
Tzi8      GAAGTGTAGCTTAAAGTAGAAAATCTGTTCATGCTTCCAATGGTTTGCACAGTGCATAGG
```

```
B73      TATGCAAACCAACAAAGGAAAACGAGGCCAAACTATGCCCCAATTGTTTCTGTCTGAGTT
HP301    TATGCAAACCAACAAAGGAAAACGAGGCCAAACTATGCCCCAATTGTTTCTGTCTGAGTT
OH7B     TATGCAAACCAACAAAGGAAAACGAGGCCAAACTATGCCCCAATTGTTTCTGTCTGAGTT
Tzi8     TATGCAAACCAACAAAGGAAAACGAGGCCAAACTATGCCCCAATTGTTTCTGTCTGAGTT

B73      TGCCAGTTTCAATTTTTTGGATCAACATAACTTGGGTATGTTGGAAATAATCCCGTGTCT
HP301    TGCCAGTTTCAATTTTTTGGATCAACATAACTTGGGTATGTTGGAAATAATCCCGTGTCT
OH7B     TGCCAGTTTCAATTTTTTGGATCAACATAACTTGGGTATGTTGGAAATAATCCCATGTCT
Tzi8     TGCCAGTTTCAATTTTTTGGATCAACATAACTTGGGTATGTTGGAAATAATCCCATGTCT

B73      AGGTTCATAGCATTTACTTTAGCTAAATTTCAGCATGGAATAATAGGAAGTAATATGCTG
HP301    AGGTTCATAGCATTTACTTTAGCTAAATTTCAGCATGGAATAATAGGAAGTAATATGCTG
OH7B     AGGTTCATAGCATTTACTTTAGCTAAATTTCAGCATGGAATAATAGGAAGTAATATGCTA
Tzi8     AGGTTCATAGCATTTACTTTAGCTAAATTTCAGCATGGAATAATAGGAAGTAATATGCTA

B73      ATTAGTTGAGTAATATCCTGCATTTATTAGGGGCTATCATTGCTTTATTTGTAGCTCACA
HP301    ATTAGTTGAGTAATATCCTGCATTTATTAGGGGCTATCATTGCTTTATTTGTAGCTCACA
OH7B     ATTAGTTGAGTAATATCCTGCATTTATTAGGGGCTATCATAGCTTTATTTGTAGCTCACA
Tzi8     ATTAGTTGAGTAATATCCTGCATTTATTAGGGGCTATCATAGCTTTATTTGTAGCTCACA

B73      TTGGTTATAATAGTAGTAGTAGAAGAGAAAATAGAAAAGGCAAGCCATTCCAGCCACCAA
HP301    TTGGTTATAATAGTAGTAGTAGAAGAGAAAATAGAAAAGGCAAGCCATTCCAGCCACCAA
OH7B     TTGGTTATAATAGTAGTAGTAGAAGAGAAAATAGAAAAGGCAAGCCATTCCAGCCACCAG
Tzi8     TTGGTTATAATAGTAGTAGTAGAAGAGAAAATAGAAAAGGCAAGCCATTCCAGCCACCAG

B73      AAACACTTGTACTATGTTCCTGTGACCTTTTGGAGGCTTTCAACCTGTCAGGAATCAGGA
HP301    AAACACTTGTACTATGTTCCTGTGACCTTTTGGAGGCTTTCAACCTGTCAGGAATCAGGA
OH7B     AAACACTTGTACTGTGTTCCTGTGACCTTTTGGAGGCTTTCAACCTGTCAGGAATCAGGA
Tzi8     AAACACTTGTACTGTGTTCCTGTGACCTTTTGGAGGCTTTCAACCTGTCAGGAATCAGGA

B73      TATTTGATGCATCCAATTATCTAAATCCAACAAATCTTGGTATTATGCTTACTGTATAAT
HP301    TATTTGATGCATCCAATTATCTAAATCCAACAAATCTTGGTATTATGCTTACTGTATAAT
OH7B     TATTTGATGCATCCAATTATCTAAATCCAACAAATCTTGGTATTATGCTTACTGTATAAT
Tzi8     TATTTGATGCATCCAATTATCTAAATCCAACAAATCTTGGTATTATGCTTATTGTATAAT

B73      CATGGTCGTATTATTTTTTAGTCTGTTGATATTTCACTGGCATAAATTGTTTCTTCTGTG
HP301    CATGGTCGTATTATTTTTTAGTCTGTTGATATTTCACTGGCATAAATTGTTTCTTCTGTG
OH7B     CATGGTCGCATTATTTTTTAGTCTGTTGATATTTCACTGGCATAAATTGTTTCTTCTGTG
Tzi8     CATGGTCGCATTATTTTTTAGTCTGTTGATATTTCACTGGCATAAATTGTTTCTTCTGTG

                                                 ↓ Acceptor i, iii, iv§, vii
B73      CTTTCATATCCACTATGTAACCCATGTTTTATTGTTTGTAGAGAACTACAATTTATGCTC
HP301    CTTTCATATCCACTATGTAACCCATGTTTTATTGTTTGTAGAGAACTACAATTTATGCTC
OH7B     CTTTCATATCCACTATGTAACCCATGTTTTATTGTTTGTAGAGAACTACAATTTATGCTC
Tzi8     CTTTCATATCCACTATGTAACCCATGTTTTATTGTTTGTAGAGAACTACAATTTATGCTC
                                   **********************************
                                                         ↑ Acceptor ii

B73      AAGTCAATGCTGCTCTTCGCAAAATAAGAGACACATCAGAGGTCCTACATGCAATTCAGC
HP301    AAGTCAATGCTGCTCTTCGCAAAATAAGAGACACATCAGAGGTCCTACATGCAATTCAGC
OH7B     AAGTCAATGCTGCTCTTCGCAAAATAAGAGACACATCAGAGGTCCTACATGCAATTCAGC
Tzi8     AAGTCAATGCTGCTCTTCGCAAAATAAGAGACACATCAGAGGTCCTACATGCAATTCAGC
         ******
```

```
B73         CTACTTTTATTCACTTATTTCCTCAAATGCACAGAAAGTATAACTACTATCTCTGGTCTG
HP301       CTACTTTTATTCACTTATTTCCTCAAATGCACAGAAAG-TTAACTACTATCTCTGGTCTG
OH7B        CTACTTTTATTCACTTATTTCCTCAAATGCACAGAAAGTATAACTACTATCTCTGGTCTG
Tzi8        CTACTTTTATTCACTTATTTCCTCAAATGCACAGAAAGTATAACTACTATCTCTGGTCTG

B73         GTGCAGTTTGTCCAATCTTTTGCATCAGAGCATCTTAAGACGCCATTAGAAGAACTTGTG
HP301       GTGCAGTTTGTCCAATCTTTTGCATCAGAGCATCTTAAGACGCCATTAGAAGAACTTGTG
OH7B        GTGCAGTTTGTCCAATCTTTTGCATCAGAGCATCTTAAGATGCCATTAGAAGAACTTGTG
Tzi8        GTGCAGTTTGTCCAATCTTTTGCATCAGAGCATCTTAAGATGCCATTAGAAGAACTTGTG

B73         AAAGGTAATAAAAACAAGTCCACCA---CTGAGTTATGGGTAGAGAAATTCTATAAGAAG
HP301       AAAGGTAATAAAAACAAGTCCACCA---CTGAGTTATGGGTAGAGAAATTCTATAAGAAG
OH7B        AAAGGTAATAAAAACAAGTC-CACCACTGAGTT--ATGGGTAGAGAAATTCTATAAGAAG
Tzi8        AAAGGTAATAAAAAACAAGTCCACCGCTGAGTTATTGGGTAGAGAAAATTCTATAAGAAG

B73         GTAACCAATCTACCGGAGCCTTTTCCCCATGATCTTGTTGAGAAGTTAGAAGAATACCTG
HP301       GTAACCAATCTACCGGAGCCTTTTCCCCATGATCTTGTTGAGAAGTTAGAAGAATACCTG
OH7B        GTAACCAATCTACCGGAGCCTTTTCCCCATGATCTTGTTGAGAAGTTAGAAGAATACCTG
Tzi8        GTAACCAATCTACCGGAGCCTTTTCCCCATGATCTTGTTGAGAAGTTAGAAGAATACCTG

B73         GATGTAAGTATTATATACCATACATTGCATAAGTGTACTTTCATGATGAAATAATGCATA
HP301       GATGTAAGTATTATATACCATACATTGCATAAGTGTACTTTCATGATGAAATAATGCATA
OH7B        GATGTAAGTATTATATACCATACATTGCATAAGTGTACTTTCATGATGAAATAATGCATA
Tzi8        GATGTAAGTATTATATACCATACATTGCATAAGTGTACTTTCATGATGAAATAATGCATA

B73         TATATGTTCTAATTTTCCTTCATCTATGGTGCCCCTTGTAGAAACTTGAAGGGCAACCCG
HP301       TATATGTTCTAATTTTCCTTCATCTATGGTGCCCCTTGTAGAAACTTGAAGGGCAACCCG
OH7B        TATATGTTCTAATTTTCCTTCATCTATGGTGCCCCTTGTAGAAACTTGAAGGGCAACCCG
Tzi8        TATATGTTCTAATTTTCCTTCATCTATGGTGCCCCTTGTAGAAACTTGAAGGGCAACCCG

B73         TGGACTTGTCTTCCTTATTGTATGACCACCAGCTGATCGATGCATATCAAAACAGTACCG
HP301       TGGACTTGTCTTCCTTATTGTATGACCACCAGCTGATCGATGCATATCAAAACAGTACCG
OH7B        TGGACTTGTCTTCCTTATTGTATGACCACCGGCTGATCGATGCATATCAAAGCAGTACCG
Tzi8        TGGACTTGTCTTCCTTATTGTATGACCACCGGCTGATCGATGCATATCAAAGCAGTACCG

B73         ACATCCTGCAAAGCACTATTTTTACACAACAGTATGTTTATATAGAGATGTTATATTTGC
HP301       ACATCCTGCAAAGCACTATTTTTACACAACAGTATGTTTATATAGAGATGTTATATTTGC
OH7B        ACATCCTGCAAAGCACTATTTTTACACAACAGTATGTTTATACAGGGATGCTATATTTGC
Tzi8        ACATCCTGCAAAGCACTATTTTTACACAACAGTATGTTTATACAGGGATGCTATATTTGC

B73         ATCTGGCTACTCTTGTGCTATCTTTTTGAAACTCATTATTTTCATTTTGTTTCATTCGAA
HP301       ATCTGGCTACTCTTGTGCTATCTTTTTGAAACTCATTATTTTCATTTTGTTTCATTCGAA
OH7B        ATCTGGCTACTCTTGTGCTATCTTTTTGAAACTCATTATTTTCATTTTGTTTCATTCGAA
Tzi8        ATCTGGCTACTCTTGTGCTATCTTTTTGAAACTCATTATTTTCATTTTGTTTCATTCGAA

B73         ACATTTTTACCCTTTCCCCCCTTCTCTTGACAGGTATGTGGAACGTGTTCTAGCTAACGA
HP301       ACATTTTTACCCTTTCCCCCCTTCTCTTGACAGGTATGTGGAACGTGTTCTAGCTAACGA
OH7B        ACATTTTTACCCTTTCCCCCCTTCTCTTGACAGGTATGTGGAACGTGTTCTAGCTAACGA
Tzi8        ACATTTTTACCCTTTCCCCCCTTCTCTTGACAGGTATGTGGAACGTGTTCTAGCTAACGA
```

```
B73         GAGAGACAAGATGAAATGCTGTAGCATTGAATATAGTCATCCAAAGCAGTCATCCCAGGC
HP301       GAGAGACAAGATGAAATGCTGTAGCATTGAATATAGTCATCCAAAGCAGTCATCCCAGGC
OH7B        GAGAGACAAGATGAAATGCTGTAGCATTGAATATAGTCATCCAAAGCAGTCATCCCAGGC
Tzi8        GAGAGACAAGATGAAATGCTGTAGCATTGAATATAGTCATCCAAAGCAGTCATCCCAGGC

B73         TTTTGTCTATGGAGGGATCCTTCTGGCTGGATTTATTGTGTATTCACTGGTTATTTTCTT
HP301       TTTTGTCTATGGAGGGATCCTTCTGGCTGGATTTATTGTGTATTCACTGGTTATTTTCTT
OH7B        TTTTGTCTATGGAGGGATCCTTCTGGCTGGATTTCTTGTGTATTCACCGGTTATTTTCTT
Tzi8        TTTTGTCTATGGAGGGATCCTTCTGGCTGGATTTCTTGTGTATTCACCGGTTATTTTCTT
                                               ************ ************
                                                             ⬆ Donor viii§

B73         CTCTTCACCTGTTCGCTAATTTTATGTCCAGATGAAGAAAGTCCTGGATGCTTTATGGTA
HP301       CTCTTCACCTGTTCGCTAATTTTATGTCCAGATGAAGAAAGTCCTGGATGCTTTATGGTA
OH7B        CTCTTCACCTGTTCGCTAATTTTATGTCCAGATGCAGAAAGTCCTGGATGCTTTATGGTA
Tzi8        CTCTTCACCTGTTCGCTAATTTTATGTCCAGATGCAGAAAGTCCTGGATGCTTTATGGTA
            ****

B73         GGCTAGGTTTGTCTAGGGTTGTCAATCTTCATGAGGGAATGACCTAGACCGTCTTGGATA
HP301       GGCTAGGTTTGTCTAGGGTTGTCAATCTTCATGAGGGAATGACCTAGACCGTCTTGGATA
OH7B        GGCTAGGTTTGTCTAGGGTTGTCAATATTCATGAGGGAATGACCTAGACTGTCTTGGATA
Tzi8        GGCTAGGTTTGTCTAGGGTTGTCAATATTCATGAGGGAATGACCTAGACTGTCTTGGATA

B73         TAGAAGGGCTTTGGCACAGTTTTAGTGCGCTAT-TTCGCGATACACAAGCAATCAGACGT
HP301       TAGAAGGGCTTTGGCACAGTTTTAGTGCGCTAT-TTCGCGATACACAAGCAATCAGACGT
OH7B        TAGAAGGGCTTTGGCACAGTTTTAGTGCGCTAT-TTCTCGATACACAAGCAATCAGACGG
Tzi8        TAGAAGGGCTTTGGCACAGTTTTAGTGCGCTATTTTCTCGATACACAAGCAATCAGACGG

B73         CTGATATATATGTAACTGATTCTTGTCTGCGATAAACTATAGTCGATTTCACAGAAGAGT
HP301       CTGATATATATGTAACTGATTCTTGTCTGCGATAAACTATAGTCGATTTCACAGAAGAGT
OH7B        CTGATATATATGTAACTGATTCTTGTCTGCGATAAACTATAGTTGATTTCACAGAAGAGT
Tzi8        CTGATATATATGTAACTGATTCTTGTCTGCGATAAACTATAGTTGATTTCACAGAAGAGT

B73         AGGTGCATGCTATGAAAATATGAAATACTTTCCCCC--ATTTTGCAACTTGTTACTGCAG
HP301       AGGTGCATGCTATGAAAATATGAAATACTTTCC-CC--ATTTTGCAACTTGTTACTGCAG
OH7B        TGGTGCATGCTATGAAAATATGAAATACTTACCCCC--ATTTTGCAACTTGTTACTGTAG
Tzi8        TGGTGCATGCTATGAAAATATGAAATACTTACCCCCCATTTTTGCAACTTGTTACTGTAG

B73         AACTGCACATAACACCACTGACAGAAACCCGTTTGTTATTAAGCCCCCCAGCTTCATGTG
HP301       AACTGCACATAACACCACTGACAGAAACCCGTTTGTTATTAAGC-CCCCAGCTTCATGTG
OH7B        AACTGCACATAACACCACTGACAGAAACCCATTTGTTAATAAGCCCCCCAGTTTCATGTG
Tzi8        AACTGCACATAACACCACTGACAGAAACCCATTTGTTAATAAGCCCCCCAGTTTCATGTG

B73         GACCAGCCAGCAGACCTGTGGCCGTGGAGTGGTGCAAGGGCGTAGTCGCTTTATTTCATG
HP301       GACCAGCCAGCAGACCTGTGGCCGTGGAGTGGTGCAAGGGCGTAGTCGCTTTATTTCACG
OH7B        GACCAGCCAGCAGACCTGTGGCCGTGGAGTGGTGCAAGGGCGTAGTCGCTTTATTTCATG
Tzi8        GACCAGCCAGCAGACCTGTGGCCGTGGAGTTGCGCAAGGGCGAGTCGCTTTAT-T-CATG

B73         GCAATCATCTCCAAAAATT-ATTTTCCTTGGCTCGTGT-GTTTTTTATCCTTTTGAAAAT
HP301       GCAATCATCTCCAAAAATT-ATTTTCCTTGGCTCGTGT-GTTTTTTATCCTTTTGAAAAT
OH7B        GCAATCAT-CTCCAAAAAATTATTTTCCTTAGCTCGTGT-GTTTTTTATCCTTTTGAAAAT
Tzi8        GCAATCATCTCCAAAAAATTATTTTCCTTAGCTCGTGTGTTTTTTTATCCTTTTGAAAAT
```

```
B73         TGCCTGACGCCAAGGCTCCACCATACGCCTGAAGCGCTGAGGCCTCAATGTCCAGCGTGT
HP301       TGCCTGACGCCAAGGCTCCACCATACGCCTGAAGCGCTGAGGCCTCAATGTCCAGCGTGT
OH7B        TGCCTAACGCCAAGGCTCCACCGTACGCCTGAAGCGATGAGGCCTCAATGTCCAGCGTGT
Tzi8        TGCCTAACGCCAAGGCTCCACCGTACGCCTGAAGCGATGAGGCCTCAATGTCCAGCGTGT

B73         TTCAATATAAAAGATTGCTTTTAGCTAGGGCAATGGTGGTTGTGCAAGCAGTATTTAGGT
HP301       TTCAATATAAAAGATTGCTTTTAGCTAGGGCAATGGTGGTTGTGCAAGCAGTATTTAGGT
OH7B        TTCAATATAAAAGATTGCTTTTAGCTAGGGCAATGGTGGTTGTGCAAGAAGTACTTAGGT
Tzi8        TTCAATATAAAAGATTGCTTTTAGCTAGGGCAATGGTGGTTGTGCAAGAAGTACTTAGGT

B73         GCAAACGTGCAAGCAAGTTCTCTGGTCCGTTTTGATTTGTTCCATCCTGCTATATATTTT
HP301       GCAAACGTGCAAGCAAGTTCTCTGGTCCGTTTTGATTTGTTCCATCCTGCTATATATTTT
OH7B        GCAAACGTGCAAGCGAGTTCTCTGGTCCGTTTTGATTTGTTCCATCCCGCTATATATTTT
Tzi8        GCAAACGTGCAAGCGAGTTCTCTGGTCCGTTTTGATTTGTTCCATCCCGCTATATATTTT

B73         GCACCAGTTTATAATTATCTTTTGGCAC--ATGATCACTACATATGTGAGTTGGCTCCAA
HP301       GCACCAGTTTATAATTATCTTTTGGCAC--ATGATCACTACATATGTGAGTTGGCTCCAA
OH7B        GCACCAGTTTATAATTATCTTTTGGCACATATGATCACTACATATGTGAGTTGGCTCCAA
Tzi8        GCACCAGTTTATAATTATCTTTTGGCACATATGATCACTACATATGTGAGTTGGCTCCAA

B73         TAATTTAACTTTGATTCCATGGAACATTTTATTTTCATCCGGACACTTCTGTCTTTCTCA
HP301       TAATTTAACTTTGATTCCATGGAACATTTTATTTTCATCCGGACACTTCTGTCTTTCTCA
OH7B        TAATTTAACTTTGATTCCATGGAACATTTTATTTTCATCCGGACACTTATGTCTTTCTCA
Tzi8        TAATTTAACTTTGATTCCATGGAACATTTTATTTTCATCCGGACACTTATGTCTTTCTCA

B73         TAAAATTT-TTATTGCTTGTAAATTTACTATTGGTGCTCAAATATTCTTTG-CTTCATCA
HP301       TAAAATTT-TTATTGCTTGTAAATTTACTATTGGTGCTCAAATATTCTTTG-CTTCATCA
OH7B        TAAAATCTTTTATTGCTTGTAAATTTACTATTGGTGCTCAAATATTCTTTGCCTTCATCA
Tzi8        TAAAATCTTTTATTGCTTGTAAATTTACTATTGGTGCTCAAATATTCTTTGCCTTCATCA

B73         AAAAAATTCCTTGATCAGTTCTGGTGATTATAACAATTTTAGTTTTTTGGTTATAACTTT
HP301       AAAAAATTCCTTGATCAGTTCTGGTGATTATAACAATTTTAGTTTTTTGGTTATAACTTT
OH7B        AAAAAATTCCTTGATCAGTTCTGGTGATTAGAACAATTTTAGTTTTTTTGTTAGAACTTT
Tzi8        AAAAAATTCCTTGATCAGTTCTGGTGATTAGAACAATTTTAGTTTTTTTGTTAGAACTTT

B73         TTCCTTTCTCAGAACATTTCTCTTTGCTTGGAGCATTTTCCTTAGTATTTAGAACAATTT
HP301       TTCCTTTCTCAGAACATTTCTCTTTGCTTGGAGCATTTTCCTTAGTATTTAGAACAATTT
OH7B        TTCCTTTCTCAGAACATTTCTCTTTGCTTGGAGCATTTTCCTTAGTATTTAGAACAATTT
Tzi8        TTCCTTTCTCAGAACATTTCTCTTTGCTTGGAGCATTTTCCTTAGTATTTAGAACAATTT

B73         ATTTTTTGATCGA--TTTTTTTCTTTGCTCAGATCAATGCTTGTTGCAACATCATAGTGC
HP301       ATTTTTTGATCGA--TTTTTTTCTTTGCTCAGATCAATGCTTGTTGCAACATCATAGTGC
OH7B        ATTTTTTGATCGAATTTTTTTTCTTTGCTCAGATCAATGCTTGTTGCAACATCATAGTGC
Tzi8        ATTTTTTGATCGAATTTTTTTTCTTTGCTCAGATCAATGCTTGTTGCAACATCATAGTGC
                             ******************************
                                           ↑ Acceptor viii[§]

B73         ACCTGATGGGGAATCCAACATGTTTCACGTGACATGTAGGGGAAGGCATCGAAAAAGGTG
HP301       ACCTGATGGGGAATCCAACATGTTTCACGTGACATGTAGGGGAAGGCATCGAAAAAGGTG
OH7B        ACCTGATAGGGAATCCAACATGTTTCACGTGACATGTAGGGGAAGGCATCGAAAAAGTTG
Tzi8        ACCTGATAGGGAATCCAACATGTTTCACGTGACATGTAGGGGAAGGCATCGAAAAAGTTG
```

```
B73         AAGGTTGTAGAAAAGGGCCCTAAAAAGGGCACGATGAAAGCCCAACTTGCTAAGAGAGGT
HP301       AAGGTTGTAGAAAAGGGCCCTAAAAAGGGCACGATGAAAGCCCAACTTGCTAAGAGAGGT
OH7B        AAGGTTGTAGAAAAGAGCACGAT-----------GAAAAAGCCAACTTGCTAAGAGAGGT
Tzi8        AAGGTTGTAGAAAAGAGCACGAT-----------GAAAAAGCCAACTTGCTAAGAGAGGT

B73         AGGAAGGCTAAGAATTGATGACATGTTGATTTCCTCGTCAGAGAAAAAAATGCTATAGCA
HP301       AGGAAGGCTAAGAATTGATGACATGTTGATTTCCTCGTCAGAGAAAAAAATGCTATAGCA
OH7B        AGGAAGGCTAAGAATTGATGACATGTTGATTTCCTCGTCAGAGAAAAAAATGCTATAGCA
Tzi8        AGGAAGGCTAAGAATTGATGACATGTTGATTTCCTCGTTAGAGAAAAAAATGCTATAGCA
```

Figure S2 Alignment of *Helitron Hel1-332* between the maize inbred lines. The alignment shows the splice junctions of alternative splicing events (i – viii) of *Hel1-332* shown in figure 3. The blue and gray shaded sequences flanking the splice sites represent exons and introns, respectively. Asterisks represent the conserved sequences beneath the highlighted regions, while the dashed lines fill the gaps in the alignment. The inbred lines are indicated on the left. The intron retention at splice sites iv and viii are marked by §.
