# Supplementary material for: Differential pre-mRNA Splicing Alters the Transcript Diversity of Helitrons Between the Maize Inbred Lines
Source: G3 (Bethesda). 2015 Jun 12;5(8):1703–11. doi: 10.1534/g3.115.018630 (PMC4528327; doi:10.1534/g3.115.018630)
Supplement: Supporting Information [file supp_g3.115.018630_FigureS3.pdf]

```
B73     TGTGAATCCTCGCCTAGGATGCCGACGGGCACGGCGCCCACGGTGGTCACCTTCTGCGCC
HP301   TGTGAATCCTCGCCTAGGATGCCGACGGGCACGGCGCCCACGGTGGTCACCTTCTGCGCC
OH7B    TGTGAATCCTCGCCTAGGATGCCGACGGGCACGACGCCCACGGTGGTCACCTTCTGCGCC
Tzi8    TGTGAATCCTCGCCTAGGATGCCGACGGGCACGGCGCCCACGGTGGTCACCTTCTGCGCC


B73     TCTTCGAGGTGGTGCTCATCGGCTCCATAGATCTGGCACCATGCTTAGCCGACCATGGCG
HP301   TCTTCGAGGTGGTGCTCATCGGCTCCATAGATCTGGCGCCATGCTTAGCCGACCATGGCG
OH7B    TCTTCGAGGTGGTGCTCATCGGCTCCATAGATCTGGCACCATGCTTAGCCGACCATGGCG
Tzi8    TCTTCGAGGTGGTGCTCATCGGCTCCATAGATCTGGCACCATGCTTAGCCGACCATGGCG


B73     AGGCCATCAAGGAGGACACCGGCCACGATCCGTCAGGTTTCTCTCCCTCCTCTTTCCCTC
HP301   AGGCCATCAAGGAGGACACCGGCCACGATCCGTCAGGTTTCTCTCCCTCCTCTTTCCCTC
OH7B    AGGCCATCAAGGAGGACATCGACCACGATCCGTCAGGTTTCTCTCCCTCCTCTTTCCCTC
Tzi8    AGGCCATCAAGGAGGACACCGGCCACGATCCGTCAGGTTTCTCTCCCTCCTCTTTCCCTC
                              *****************************
                                            ↑ Donor i
B73     TGTGTCTGTGATGTCGGGGTTGGATATGCATTGCAATGGGGGGCGTGATGGTGCGGTTGG
HP301   TGTGTCTGTGATGTCGGGGTTGGATATGCATTGCAATGGGGGGCGTGATGGTGCGGTTGG
OH7B    TGTGTCTGTGATGTCGGGGTTGGATATGCATTGCAATGGGGG-CGTGATGGTGCAGTTGG
Tzi8    TGTGTCTGTGATGTCGGGGTTGGATATGCATTGCAATGGGGGGCGTGATGGTGCGGTTGG


B73     AACTCATAACCATCCCCAATCAGTGTGAAGCGGTGGTGTGGGTGTCCCACTTGGGAGACT
HP301   AACTCATAACCATCCCCAATCAGTGTGAAGCGGTGGTGTGGGTGTCCCACTTGGGAGACT
OH7B    AACTCATAACCGTCCCCCATCCGTGTGAAGCGGTGGTGTGGGTGTCCCACTTGGGAGACT
Tzi8    AACTCATAACCATCCCCAATCAGTGTGAAGCGGTGGTGTGGGTGTCCCACTTGGGAGACT


B73     CGAGCACTGCGCATCCGGCAGCTTGTCTGGTGGGGGTCTATAGGGATTTTTTT-ACTTTC
HP301   CGAGCACTGCGCATCCGGCAGCTTGTCTGGTGGGGGTCTATAGGGATTTTTTT-ACTTTC
OH7B    CGGGCACTTCGCATCCGGCAGCTTGTCTGGTGGGGGTCTATAGGGATTTTTTTTACTTTC
Tzi8    CGAGCACTGCGCATCCGGCAGCTTGTCTGGTGGGGGTCTATAGGGATTTTTTT-ACTTTC


B73     TCTACTCTCAACTCGAGTGTTTCAAAAGCATCTTACACCCGTCTGTCAGATGGCTTTAAA
HP301   TCTACTCTCAACTCGAGTGTTTCAAAAGCATCTTACACCCGTCTGTCAGATGGCTTTAAA
OH7B    TCTACTCTCAACTCTAGTGTTTCAAAAGCATCTTACACCCGACTGTCAGATGGCTTTAAA
Tzi8    TCTACTCTCAACTCGAGTGTTTCAAAAGCATCTTACACCCGTCTGTCAAATGGCTTTAAA


B73     ACACTCGAAACTGGATAGATAACAGAATCGAGTGTTTATAGGCTATGGAACACTCAATTC
HP301   ACACTCGAAACTGGATAGATAACAGAATCGAGTGTTTATAGGCTATGGAACACTCAATTC
OH7B    ACACTCGAAACTAGATAGATAACAGAATCGAGTGTTTATAGGCTATGGAAAGCTCAATTC
Tzi8    ACACTCGAAACTGGATAGATAACAGAATCGAGTGTTTATAGGCTATGGAACACTCAATTC


B73     TCATATCAGATAGAAAAAAA-CAAACCAAATTATTTCTATGGAAGCCAAATTGGAAGGTT
HP301   TCATATCAGATAGAAAAAAA-CAAACCAAATTATTTCTATGGAAGCCAAATTGGAAGGTT
OH7B    TCATATCAGATAGAAAAAAAACTAACCAAATTATTTCTATGGAAGCCAAATTGGAAGGTT
Tzi8    TCATATCAGATAGAAAAAAA-CAAACCAAATTATTTCTATGGAAGCCAAATTGGAAGGTT


B73     TGCAGAGGGGAACCAAATAAAACATGCATACAAACAAAAAATAGAACAACAAGAAATAAA
HP301   TGCAGAGGGGAACCAAATAAAACATGCATACAAACAAAAAATAGAACAACAAGAAATAAA
OH7B    TGCAGAGGGGAACCAAATAAAACATGCATACAAACAAAAAATAGAACAGCAAGAAATAAA
Tzi8    TGCAGAGGGGAACCAAATAAAACATGCATACAAACAAAAAATAGAACAACAAGAAATAAA
```

```
B73     CAGATAACTGTTCGTGCAATCCAAATGATCATTCTCAATAATAAACAACCAATTTACATC
HP301   CAGATAACTGTTCGTGCAATCCAAATGATCATTCTCAATAATAAACAACCAATTTACATC
OH7B    CAGATAACTGTTCGTGCAATCCAAATGATCATTCTCAATAATAAACAACCAATTTACATC
Tzi8    CAGATAACTGTTCGTGCAATCCAAATGATCATTCTCAATAATAAACAACCAATTTACATC

B73     AGTCGGATACACAAC----ACACAAACACAGATAGTAAAGCATGATATAGTAAGATGTAA
HP301   AGTCGGATACACAAC----ACACAAACACAGATAGTAAAGCATGATATAGTAAGATGTAA
OH7B    AGTCGGATACACAACTTACACACAAACACAGATAGTAAAGCATGCTATAGTAAGATGTAA
Tzi8    AGTCGGATACACAAC----ACACAAACACAGATAGTAAAGCATGATATAGTAAGATGTAA

B73     TACGGAACTACTGGGCCAAAGCAGACCGGACGTAATCAAATCGAAGAAGGGATTCACAGA
HP301   TACGGAACTACTGGGCCAAAGCAGACCGGACGTAATCAAATCGAAGAAGGGATTCACAGA
OH7B    TACGGAACTACTGGGCCAAAGCAGACCGGACGTAATCAAATCGAAGAAGGGATTCACAGA
Tzi8    TACGGAACTACTGGGCCAAAGCAGACCGGACGTAATCAAATCGAAGAAGGGATTCACAGA

B73     TTCAAATAAGAGATGGAATTGTCTGGTGCCTTGGATTGCTGATTGAGGTGATGCAGGGCC
HP301   TTCAAATAAGAGATGGAATTGTCTGGTGCCTTGGATTGCTGATTGAGGTGATGCAGGGCC
OH7B    TTCAAATAAGAGATGGAATTGTCTGGTGCCTTGGATCGCTGATTGAGGTGATGC------
Tzi8    TTCAAATAAGAGATGGAATTGTCTGGTGCCTTGGATTGCTGATTGAGGTGATGCAGGGCC

B73     GGTCCTGAGGTTTTATGGACCCGGGGCAAAACAAAAAATTGGGGCCCTTTAAAACTGTAT
HP301   GGTCCTGAGGTTTTATGGACCCGGGGCAAAACAAAAAATTGGGGCCCTTTAAAACTGTAT
OH7B    ------------------------------------------------------------
Tzi8    GGTCCTGAGGTTTTATGGACCCGGGGCAAAACAAAAAATTGGGGCCCTTTAAAACTGTAT

B73     ATATATTTTTTACTTTCTTCTAAGTACATAAGCAAAATTTTGAAAGATAAAAGTCTATAA
HP301   ATATATTTTTTACTTTCTTCTAAGTACATAAGCAAAATTTTGAAAGATAAAAGTCTATAA
OH7B    ------------------------------------------------------------
Tzi8    ATATATTTTTTACTTTCTTCTAAGTACATAAGCAAAATTTTGAAAGATAAAAGTCTATAA

B73     TATATCAAAATAACTTCAAATCTTTGTACATATTACTTCAAAAAAATTCTAACATTTCTT
HP301   TATATCAAAATAACTTCAAATCTTTGTACATATTACTTCAAAAAAATTCTAACATTTCTT
OH7B    ------------------------------------------------------------
Tzi8    TATATCAAAATAACTTCAAATCTTTGTACATATTACTTCAAAAAAATTCTAACATTTCTT

B73     GATGCAAAGTCATTGATAATAGTGTTATTATCAACCTCGTCTAATAATTTTTCTCAATAC
HP301   GATGCAAAGTCATTGATAATAGTGTTATTATCAACCTCGTCTAATAATTTTTCTCAATAC
OH7B    ------------------------------------------------------------
Tzi8    GATGCAAAGTCATTGATAATAGTGTTATTATCAACCTCGTCTAATAATTTTTCTCAATAC

B73     ATAAAGTAGCTAATTCGTTGAGACGATCTTGAGACATCGTTGACCTTAAATAGTTCTTCA
HP301   ATAAAGTAGCTAATTCGTTGAGACGATCTTGAGACATCGTTGACCTTAAATAGTTCTTCA
OH7B    ------------------------------------------------------------
Tzi8    ATAAAGTAGCTAATTCGTTGAGACGATCTTGAGACATCGTTGACCTTAAATAGTTCTTCA

B73     ACAATTTTAACTTTGAAAAGCTTATCTTAGTCGATGCCACAGTCACGTCACATGCACAGT
HP301   ACAATTTTAACTTTGAAAAGCTTATCTTAGTCGATGCCACAGTCACGTCACATGCACAGT
OH7B    ------------------------------------------------------------
Tzi8    ACAATTTTAACTTTGAAAAGCTTATCTTAGTCGATGCCACAGTCACGTCACATGCACAGT
```

```
B73      AAATAATATTCGATAAGTGATTGAAATATTTGGATACGAATCTATTTGCCGAACATATTA
HP301    AAATAATATTCGATAAGTGATTGAAATATTTGGATACGAATCTATTTGCCGAACATATTA
OH7B     ------------------------------------------------------------
Tzi8     AAATAATATTCGATAAGTGATTGAAATATTTGGATACGAATCTATTTGCCGAACATATTA

B73      AAAAATCTCCATAGAAGACATTAGTCTTTCAGGGCACGAGGCCGGTGGCCAGCAACAGGT
HP301    AAAAATCTCCATAGAAGACATTAGTCTTTCAGGGCACGAGGCCGGTGGCCAGCAACAGGT
OH7B     ------------------------------------------------------------
Tzi8     AAAAATCTCCATAGAAGACATTAGTCTTTCAGGGCACGAGGCCGGTGGCCAGCAACAGGT

B73      CGTCAGGCCGATGTCGATCGTCGGCCAGCCAGACGGACGAAACCAAGGCGGTGGCAGCAC
HP301    CGTCAGGCCGATGTCGATCGTCGGCCAGCCAGACGGACGAAACCAAGGCGGTGGCAGCAC
OH7B     ------------------------------------------------------------
Tzi8     CGTCAGGCCGATGTCGATCGTCGGCCAGCCAGACGGACGAAACCAAGGCGGTGGCAGCAC

B73      AACGATTAAAATTCTAGACACAATTTTAGTAAGTTAAATAATTGAAAAATAGGACCATTG
HP301    AACGATTAAAATTCTAGACACAATTTTAGTAAGTTAAATAATTGAAAAATAGGACCATTG
OH7B     ------------------------------------------------------------
Tzi8     AACGATTAAAATTCTAGACACAATTTTAGTAAGTTAAATAATTGAAAAATAGGACCATTG

B73      TAACTTTAGCAGGTCAGACCTAATACCTAAGACTATATATACATGTACACTACTTGAAGG
HP301    TAACTTTAGCAGGTCAGACCTAATACCTAAGACTATATATACATGTACACTACTTGAAGG
OH7B     ------------------------------------------------------------
Tzi8     TAACTTTAGCAGGTCAGACCTAATACCTAAGACTATATATACATGTACACTACTTGAAGG

B73      GCCCTTCCAAAATCGGGGGCCCGGTGCGGCCGCTCCAGTTGCCCCCCTCCAGGTCCGGCC
HP301    GCCCTTCCAAAATCGGGGGCCCGGTGCGGCCGCTCCAGTTGCCCCCCTCCAGGTCCGGCC
OH7B     ------------------------------------------------------------
Tzi8     GCCCTTCCAAAATCGGGGGCCCGGTGCGGCCGCTCCAGTTGCCCCCCTCCAGGTCCGGCC

B73      CTGGGTGATGCCAGGGATGAGCTAGAATTTCTTCAATACTTGATGCGTGTGTTGACTGTT
HP301    CTGGGTGATGCCAGGGATGAGCTAGAATTTCTTCAATACTTGATGCGTGTGTTGACTGTT
OH7B     -----------CAGGGATGAGCTAGAATTTCTTCAATACTTGATGCGTGTGTTGACTGTT
Tzi8     CTGGGTGATGCCAGGGATGAGCTAGAATTTCTTCAATACTTGATGCGTGTGTTGACTGTT

B73      GAAGACTACAGCCCATCATGCATTTAGCTTATTAAAATAATGGTACCATTTTTGTAATGG
HP301    GAAGACTACAGCCCATCATGCATTTAGCTTATTAAAATAATGGTACCATTTTTGTAATGG
OH7B     GAAGACTACAGCCCATCATGCATTTAGCTTATTAAAATAATGGTACCATTTTTGTAATGG
Tzi8     GAAGACTACAGCCCATCATGCATTTAGCTTATTAAAATAATGGTACCATTTTTGTAATGG

B73      CTTGATGAA---TCATTAAGCTGGAGTTTTGTAGTGGCTTATTTTGTCCATGCTCCATCC
HP301    CTTGATGAA---TCATTAAGCTGGAGTTTTGTAGTGGCTTATTTTGTCCATGCTCCATCC
OH7B     CTTGATGATCATTAATTAAGCTGGAGTTTTGTAGTGTCTTATTTTGTCCATGCTCCATCC
Tzi8     CTTGATGAA---TCATTAAGCTGGAGTTTTGTAGTGGCTTATTTTGTCCATGCTCCATCC

B73      AAAACAGATTCATACCCATGCCCTT-CTCTAATCTGATCAAGTTATTCTTATCTTGAGTT
HP301    AAAACAGATTCATACCCATGCCCTT-CTCTAATCTGATCAAGTTATTCTTATCTTGAGTT
OH7B     AAAACAGATTCATACCCATGCCCTT-CTCTAATCTGATCAAGCTATTCTTATCTTGAGTT
Tzi8     AAAACAGATTCATACCCATGCCCTTTCTCTAATCTGATCAAGTTATTCTTATCTTGAGTT
```

```
B73      CTTGGCTAACCTCAGCGTCTATCTGTTTGATTACTCATATTTCATAATAAATTGATGTAT
HP301    CTTGGCTAACCTCAGCGTCTATCTGTTTGATTACTCATATTTCATAATAAATTGATGTAT
OH7B     CTTGGCTAACCTCAGCGTCTATCTGTTTGATTACTCATATTCCATAATAAATTGATGTAT
Tzi8     CTTGGCTAACCTCAGCGTCTATCTGTTTGATTACTCATATTTCATAATAAATTGATGTAT


B73      GTTTCTGCAATTTTGC--------TTATACATGCCTATTTTCATTATGGAGTTACTTACT
HP301    GTTTCTGCAATTTTGCAATTTTGCTTATACATGCCTATTTTCATTATGGAGTTACTTACT
OH7B     GTTTCTGCAATTTTGC--------TTATACATGCCTATTTTCATTATGGAGTTACTTACG
Tzi8     GTTTCTGCAATTTTGC--------TTATACATGCCTATTTTCATTATGGAGTTACTTACT


B73      TATCTGTAATTTTGTTTGTCCGCCCTGTGCAGATGATGGAGCCCACGAGCCATTTTCACG
HP301    TATCTGTAATTTTGTTTGTCCGCCCTGTGCAGATGATGGAGCCCACGAGCCATTTTCACG
OH7B     TATCTGTAATTTTGTTTGTCCGTCCTGTGCAGATGATGGAGCCCACGAGCCATTTTCACG
Tzi8     TATCTGTAATTTTGTTTGTCCGCCCTGTGCAGATGATGGAGCCCACGAGCCATTTTCACG
                          ***** ************************
                                         ↑ Acceptor i

B73      TAAACTTCTTGAAGATAAGCCTCGGATCACGTATGTTTAGAATTAAAACTTTTAAGTTTT
HP301    TAAACTTCTTGAAGATAAGCCTCGGATCACGTATGTTTAGAATTAAAACTTTTAAGTTTT
OH7B     TAAACTTCTTGAAGATAAGCCTCGGATCACGTATGTTTAGAATTAGAACTTTTAAGTTTT
Tzi8     TAAACTTCTTGAAGATAAGCCTCGGATCACGTATGTTTAGAATTAAAACTTTTAAGTTTT
                        ******************************
                                       ↑ Donor ii

B73      TTGATGTATAAAATGAAAACAATGTGTGTCTTAGTCTGATGATGTTATTGGTTGACTATG
HP301    TTGATGTATAAAATGAAAACAATGTGTGTCTTAGTCTGATGATGTTATTGGTTGACTATG
OH7B     TTGATGTATAAAATGAAAACAATGTGTGTCTCAGTCTGATGATGTTATTGGTTGACTATG
Tzi8     TTGATGTATAAAATGAAAACAATGTGTGTCTTAGTCTGATGATGTTATTGGTTGACTATG


B73      TTGTGTATGATTTTGAAATACATTTAGAGAAGAAATGGTCCGTGGATATATGAGCAACAC
HP301    TTGTGTATGATTTTGAAATACATTTAGAGAAGAAATGGTCCGTGGATATATGAGCAACAC
OH7B     CTGTGTATGATTTTGAAATACATTTAGAGAAGAAATGGTCCGTGGATATATGAGCAACAC
Tzi8     TTGTGTATGATTTTGAAATACATTTAGAGAAGAAATGGTCCGTGGATATATGAGCAACAC
                     ******************************
                                    ↑ Acceptor ii

B73      TGAGCTTGAGACCGCTGTACATGCCTTTGGAAGTCGTTGTTCCAATATCTCCAGGGTGTA
HP301    TGAGCTTGAGACCGCTGTACATGCCTTTGGAAGTCGTTGTTCCAATATCTCCAGGGTGTA
OH7B     TGAGCTTGAGACTGCTGTACATGCCTTTGGAAGTCGTTGTTCCAATATCTCCAGGGTGTA
Tzi8     TGAGCTTGAGACCGCTGTACATGCCTTTGGAAGTCGTTGTTCCAATATCTCCAGGGTGTA
                                                         ************

B73      CAGGTACACGATGTATAATGTTTTACTTCTGTGATAATTACATTTGGAGCTTCTAAACAA
HP301    CAGGTACACGATGTATAATGTTTTACTTCTGTGATAATTACATTTGGAGCTTCTAAACAA
OH7B     CAGGTACACGATGTATAATGTTTTACTTCTGTGATAATTACATTTGGAGCTTCTAAACAA
Tzi8     CAGGTACACGATGTATAATGTTTTACTTCTGTGATAATTACATTTGGAGCTTCTAAACAA
         ******************
            ↑ Donor iii

B73      ACTTTTAAGACCATCAGTTTTTAAGGACATGTAAATGACTGTGTCATTGGTGGGTAGTAC
HP301    ACTTTTAAGACCATCAGTTTTTAAGGACATGTAAATGACTGTGTCATTGGTGGGTAGTAC
OH7B     ACTTTTAAGACCATCAGTTTTTAAGGACATGTAAATGACTGTGTCATTGGTGGGTAGTAC
Tzi8     ACTTTTAAGACCATCAGTTTTTAAGGACATGTAAATGACTGTGTCATTGGTGGGTAGTAC


B73      CGTAGTAATATAAGATCAGGAGCGACTGCGTCTTTGACAGACAAAGGCCCAGCATGGGCA
HP301    CGTAGTAATATAAGATCAGGAGCGACTGCGTCTTTGACAGACAAAGGCCCAGCATGGGCA
OH7B     TGTAGTAATATAAGATCAGGAGCGACTGCGTCTTTGACAGACAAAGGCCCAACATGGACA
Tzi8     CGTAGTAATATAAGATCAGGAGCGACTGCGTCTTTGACAGACAAAGGCCCAGCATGGGCA
```

```
B73     GCATCGTCATGAGAGCGAGAGCGGAGAAGGAGCTTTGGGAGATGGCTGGAGGCTGGAGCT
HP301   GCATCGTCATGAGAGCGAGAGCGGAGAAGGAGCTTTGGGAGATGGCTGGAGGCTGGAGCT
OH7B    GCATCGTCATGAGAGCGAGAGCGGAGAGGGAGCTTTGGGAGATGGCTGGAGGCTGGAGCT
Tzi8    GCATCGTCATGAGAGCGAGAGCGGAGAAGGAGCTTTGGGAGATGGCTGGAGGCTGGAGCT

B73     AAGAGCATGTCTCTCCTTATGGCTCCGGCTCCAGGGTTGTAGGGTCCCCTTAGTAGCGAT
HP301   AAGAGCATGTCTCTCCTTATGGCTCCGGCTCCAGGGTTGTAGGGTCCCCTTAGTAGCGAT
OH7B    AAGAGCATGTCTCTCCTTATGGCTCCGGCTCCAGGGTTGTAGGGTCCCCTTAGTAGCGAT
Tzi8    AAGAGCATGTCTCTCCTTATGGCTCCGGCTCCAGGGTTGTAGGGTCCCCTTAGTAGCGAT

B73     GTGGTCGTCATTATTTGTTTAGTCGTATGAGGTATGACCCCATTTTTTGTATTCTTGTTT
HP301   GTGGTCGTCATTATTTGTTTAGTCGTATGAGGTATGACCCCATTTTTTGTATTCTTGTTT
OH7B    GTGGTCGCCATTATTTGTTTAGTCGTATGAGGTATGACCCCATTTTTTGTATTCTTGTTT
Tzi8    GTGGTCGTCATTATTTGTTTAGTCGTATGAGGTATGACCCCATTTTTTGTATTCTTGTTT

B73     TTGGCCACCGTAAGTGGTTTTATCTTGTAACGGCCGGCATGGACCTTAACCACTTTCTTC
HP301   TTGGCCACCGTAAGTGGTTTTATCTTGTAACGGCCGGCATGGACCTTAACCACTTTCTTC
OH7B    TTGGCCACCTTAAGTGGTTTTATCTTGTAACGGTCGGCATGGACCTTAACCACTTTCTTC
Tzi8    TTGGCC-CCGTAAGTGGTTTTATCTTGTAACGGCCGGCATGGACCTTAACCACTTTCTTC

B73     TTAATATAATGACGCGCAGCTTTCCTGCGCTTCCGAGAAAAATAAAGGATTTAAAAATCA
HP301   TTAATATAATGACGCGCAGCTTTCCTGCGCTTCCGAGAAAAATAAAGGATTTAAAAATCA
OH7B    TTAATATAATGACGCACAGCTCTCCTGCGCTTCCGAGAAAAATAAAGGATTTAAAAATCA
Tzi8    TTAATATAATGACGCGCAGCTTTCCTGCGCTTCCGAGAAAAATAAAGGATTTAAAAATCA

B73     TTATCAACCATGAGAGAAACAACATTGGATGCAGTCATGTTGATAGGTAACAACAAATGG
HP301   TTATCAACCATGAGAGAAACAACATTGGATGCAGTCATGTTGATAGGTAACAACAAATGG
OH7B    TTATCAACCATGAGAGAAACAACATTGCATGCAGTCATGTTGATAGGTAACAGCAAATGG
Tzi8    TTATCAACCATGAGAGAAACAACATTGGATGCAGTCATGTTGATAGGTAACAACAAATGG

B73     ATAAAATGAAACATGTAGCTTTTTGAAAAAAAAACTAATCATGTAGCGCCAACTTATAAC
HP301   ATAAAATGAAACATGTAGCTTTTTGAAAAAAAAACTAATCATGTAGCGCCAACTTATAAC
OH7B    ATAAAATGAAACATGTAGCTTTTTGAAAAAAAA-CTAATCATGAAGCGCCAACTTATAAC
Tzi8    ATAAAATGAAACATGTAGCTTTTTGAAAAAAAAACTAATCATGTAGCGCCAACTTATAAC

B73     ATAATTATATCC--ATAGGATACATGTAGTAATTCTGTTTTACTTCAGATTGTTTTTGGA
HP301   ATAATTATATCCCCATAGGATACATGTAGTAATTCTGTTTTACTTCAGATTGTTTTTGGA
OH7B    ATAATTATATCC--ATAGGATACATGTAGTAATTCTGTTTTACTTCAGATTGTTTTTGGA
Tzi8    ATAATTATATCC--ATAGGATACATGTAGTAATTCTGTTTTACTTCAGATTGTTTTTGGA

B73     GGAGAAAACTGTAGG-TTTTACTTCAGATTGTTGAAATGTTCC-ATGTTCATTGG-CCAT
HP301   GGAGAAAACTGTAGG-TTTTACTTCAGATTGTTGAAATGTTCC-ATGTTCATTGG-CCAT
OH7B    GGAGAAAACTGTAGG-TTTTACTTCAGATTGTTGAAATGTTCC-ATGTTCATTGG-CCAT
Tzi8    GGAGAAAACTGTAGGGTTTTACTTCAGATTGTTGAAATGTTCCCATGTTCATTGGGCCAT

B73     GATTAGCATTTTTAAGCATTTCCTGAGTAACAGAATAATTTGGTTTCAATCTCTTTATTC
HP301   GATTAGCATTTTTAAGCATTTCCTGAGTAACAGAATAATTTGGTTTCAATCTCTTTATTC
OH7B    GATTAGCATTTTTAAGCATTTCCTGAGTAACAGAATAATTTGGTTTCAATCTCTTTATTC
Tzi8    GATTAGCATTTTTAAGCATTTCCTGAGTAACAGAATAATTTGGTTTCAATCTCTTTATTC
```

```
B73     TGTTGGTTTGTTATACAACTTCTGTACTGTCCTTGATTATCTCATTTTTTAAGTGCACTG
HP301   TGTTGGTTTGTTATACAACTTCTGTACTGTCCTTGATTATCTCATTTTTTAAGTGCACTG
OH7B    TGTTGGTTTGTTATACAACTTCTGTACTGTCCTTGGTTATCTCATTTTTTAAGTGCACTG
Tzi8    TGTTGGTTTGTTATACAACTTCTGTACTGTCCTTGATTATCTCATTTTTTAAGTGCACTG

B73     CTTCTTTCTTTATTTTGTGCCCTCCAGTTTTCTCATATGTCCACACTTCACTTACATTTT
HP301   CTTCTTTCTTTATTTTGTGCCCTCCAGTTTTCTCATATGTCCACACTTCACTTACATTTT
OH7B    CTTCTTTCTTTATTTTGTGCCCTCCAGTTTTCTCATATGTCCACACTTCACTTACATTTT
Tzi8    CTTCTTTCTTTATTTTGTGCCCTCCAGTTTTCTCATATGTCCACACTTCACTTACATTTT

B73     -ATTTCTTCTCTCTTTGAGGGATAACACAAACCTGCTATCAGCATTGGAAAGAGTGTGAA
HP301   -ATTTCTTCTCTCTTTGAGGGATAACACAAACCTGCTATCAGCATTGGAAAGAGTGTGAA
OH7B    TATTTCTTCTCTCTTTGAGGGATAACACAAACCTGCTATCAGCATTGGAAAGAGTGTGAA
Tzi8    -ATTTCTTCTCTCTT-GAGGGATAACACAAACCTGCTATCAGCATTGGAAAGAGTGTGAA
            *********** ******************                        **
                           ↑ Acceptor iii

B73     TCATTTTCCATTGGTACATAACTTAATTGGGTTACATTTTTGTATGTTTTTCTTAGTATC
HP301   TCATTTTCCATTGGTACATAACTTAATTGGGTTACATTTTTGTATGTTTTTCTTAGTATC
OH7B    TCATTTTCCATTGGTACTTAACTTAATTGGGTTACATTTTTGTATGTTTTTCTTAGTATC
Tzi8    TCATTTTCCATTGGTACATAACTTAATTGGGTTACATTTTTGTATGTTTTTCTTAGTATC
        ***************** **********
                     ↑ Donor iv

B73     AAATTTACACACTGGCGAACACTTCTGGTTATTCTTGTAGTGGGTGATTGAAATATCAGA
HP301   AAATTTACACACTGGCGAACACTTCTGGTTATTCTTGTAGTGGGTGATTGAAATATCAGA
OH7B    AAATTTACACACTGGGGAACACTTCTGGTTATTCTTGTAGTGGGTGATTGAAATATCAGA
Tzi8    AAATTTACACACTGGCGAACACTTCTGGTTATTCTTGTAGTGGGTGATTGAAATATCAGA
                                 ******************************
                                        ↑ Acceptor iv

B73     CAAGCCCAAGCAAAGGGAATCTGAACCAGCATTCAAGGTTTGTGGTGACATTTTCTTAAC
HP301   CAAGCCCAAGCAAAGGGAATCTGAACCAGCATTCAAGGTTTGTGGTGACATTTTCTTAAC
OH7B    CAAGCCCAGGAAAAGGGAATCTGAACCAGCATTCAAGGTTTGTGGTGACATTTTCTTAAC
Tzi8    CAAGCCCAAGCAAAGGGAATCTGAACCAGCATTCAAGGTTTGTGGTGACATTTTCTTAAC
                              ******************************
                                     ↑ Donor v

B73     GCTATTGAGTGCGGCTTTCCTTTTTCGTAATTTTGACAGTGGAAATAAACTCTTGGTTGG
HP301   GCTATTGAGTGCGGCTTTCCTTTTTCGTAATTTTGACAGTGGAAATAAACTCTTGGTTGG
OH7B    GCTATTGAGTGCGGCTTTCCTTTTTCGTAATTTCGACAGTGGAAATAAACTCTTGGTTGG
Tzi8    GCTATTGAGTGCGGCTTTCCTTTTTCGTAATTTTGACAGTGGAAATAAACTCTTGGTTGG

B73     AGATTTCCTGTGTCGATCTTCTATTTTTTTGTTTAACCCTTGTCTAAAAGGGTAGTTATG
HP301   AGATTTCCTGTGTCGATCTTCTATTTTTTTGTTTAACCCTTGTCTAAAAGGGTAGTTATG
OH7B    AGATTTCCTATGTCGATCTTCTATTTTTTTGTTTAACCCTTGTCTAAAAGGGTAGTTATG
Tzi8    AGATTTCCTGTGTCGATCTTCTATTTTTTTGTTTAACCCTTGTCTAAAAGGGTAGTTATG

B73     TTCAATGGCAATGCAATGCCTTTACATCATTTTCTTGAATTCAACTTCCATGATGCCACA
HP301   TTCAATGGCAATGCAATGCCTTTACATCATTTTCTTGAATTCAACTTCCATGATGCCACA
OH7B    TTCAATGGCAATGCAATGCCTTTACATCATTTTCTTGAATTCAACTTCCATGATGCCACA
Tzi8    TTCAATGGCAATGCAATGCCTTTACATCATTTTCTTGAATTCAACTTCCATGATGCCACA

B73     TCTCGAGCACGGAGTAGCGTAGGCTCACACTTAACACATATTTCTGCAGTGGCAAACATG
HP301   TCTCGAGCACGGAGTAGCGTAG-CTCACACTTAACACATATTTCTGCAGTGGCAAACATG
OH7B    TCTCGAGCACGGAGTAGCGTAGGCTCACACTTAACACATATTTCTGCAGTGGCAAACATG
Tzi8    TCTCGAGCACGGAGTAGCGTAGGCTCACACTTAACACATATTTCTGCAGTGGCAAACATG
```

```
B73     CAATCATGTAATGCCATATTGGCAGTTACTAAACACATATTTTTGAAAGGATGTAATATA
HP301   CAATCATGTAATGCCATATTGGCAGTTACTAAACACATATTTTTGAAAGGATGTAATATA
OH7B    CAATCATGTAATGCCATATTGGCAGTTACTAAACACATATTTTTGAAAGGATGTAATATA
Tzi8    CAATCATGTAATGCCATATTGGCAGTTACTAAACACATATTTTTGAAAGGATGTAATATA

B73     CCCAAAGATTTAGTGTTGAATAGGAGCGAATGGAAAACAACTATCCACGTGCCTGAACCT
HP301   CCCAAAGATTTAGTGTTGAATAGGAGCGAATGGAAAACAACTATCCACGTGCCTGAACCT
OH7B    CCCAAAGATTTAGTGTTGAATAGGAACGAATGGAAAACAACTATCCACGTGCCTGAACCT
Tzi8    CCCAAAGATTTAGTGTTGAATAGGAGCGAATGGAAAACAACTATCCACGTGCCTGAACCT

B73     TGATTTGTGGTTTCTGTTGGGTTTCAACTCTAGCCTACATCAACTTGCTTGGGACTAAAA
HP301   TGATTTGTGGTTTCTGTTGGGTTTCAACTCTAGCCTACATCAACTTGCTTGGGACTAAAA
OH7B    TGATTTGTGGTTTCTGTTGGCTTTCAACTCTAGCCTACATCAACTTGCTTGGGACTAAAA
Tzi8    TGATTTGTGGTTTCTGTTGGGTTTCAACTCTAGCCTACATCAACTTGCTTGGGACTAAAA

B73     -GACTTTGTTGTTG-TTGTACA-CATGCAGTTCATTGGAAA---------CGTTCATGGT
HP301   AGACTTTGTTGTTG-TTGTACAACATGCAGTTCATTGGAAAACGTTGAAACGTTCATGGT
OH7B    -GACTTTGTTGTTG-TTGTACA-CATGCAGTTCATTGTAAA---------TGTTCATGGT
Tzi8    -GACTTTGTTGTTGGTTGTACA-CATGCAGTTCATTGGAA----------CGTTCATGGT
                     * ******* ************** **
                                ↑ Acceptor v

B73     GATGAGCCTGTTGCAAGAGAGGTTCTTATGCATCTTGCAAATTGGCTGTGTGATAACTAT
HP301   GATGAGCCTGTTGCAAGAGAGGTTCTTATGCATCTTGCAAATTGGCTGTGTGATAACTAT
OH7B    GATGAGCCTGTTGGAAGAGAGGTTCTTATGCATCTTGCAAATTGGCTGTGTGATAACTAT
Tzi8    GATGAGCCTGTTGCAAGAGAGGTTCTTATGCATCTTGCAAATTGGCTGTGTGATAACTAT

B73     CTGAAAGATTCACTGGTAAGCAACATTCCTCTTTGTTACTTGCTCTTTGGTCACCCTGCA
HP301   CTGAAAGATTCACTGGTAAGCAACATTCCTCTTTGTTACTTGCTCTTTGGTCACCCTGCA
OH7B    CTGAAAGATTCACTGGTAAGCA-CATTCCTCTTTGTTACTTGCTCTTTGGTCAC--TGCA
Tzi8    CTGAAAGATTCACTGGTAAGCAACATTCCTCTTTGTTACTTGCTCTTTGGTCACCCTGCA
        ********************** *******
                     ↑ Donor vi

B73     GTTTACCAATAGCCAATCTCTTGTAATTTTTAGCTTTGCAATAACTACCATTTGTTTTTA
HP301   GTTTACCAATAGCCAATCTCTTGTAATTTTTAGCTTTGCAATAACTACCATTTGTTTTTA
OH7B    GTTTACCAATAGCCAATCTCTTGTAATTTTTAGCTTTGCAATAACTACTTTTTGTTTTTA
Tzi8    GTTTACCAATAGCCAATCTCTTGTAATTTTTAGCTTTGCAATAACTACCATTTGTTTTTA

B73     TTGAGTTGTTGTTCTGATGATATCATACTTTTTCTACAAATACAATAACACGTGAAAGTT
HP301   TTGAGTTGTTGTTCTGATGATATCATACTTTTTCTACAAATACAATAACACGTGAAAGTT
OH7B    TTGAGTTGTTGTTCTGATGATACCATACTTTTGCTACAAATACAATAACACGTGAAAGTT
Tzi8    TTGAGTTGTTGTTCTGATGATATCATACTTTTTCTACAAATACAATAACACGTGAAAGTT

B73     ATTTGAAATATGAAATGAAATTCTAAAGGACGCTGTTTTTACTTAGTTTCCAATATATTG
HP301   ATTTGAAATATGAAATGAAATTCTAAAGGACGCTGTTTTTACTTAGTTTCCAATATATTG
OH7B    ATTTGAAATATGAAATGAAATTCTAAAGGACGTTGTTTTTACTTAGTTTCCAATATATTG
Tzi8    ATTTGAAATATGAAATGAAATTCTAAAGGATGCTGTTTTTACTTAGTTTCCAATATATTG

B73     TTCAACATTAAGATATTGTGTTCCATGTAACTATGATGTATAACACAACATATTTTCATT
HP301   TTCAACATTAAGATATTGTGTTCCATGTAACTATGATGTATAACACAACATATTTTCATT
OH7B    TTCAACATTAAGATACTGTGTTCCATGTAACTATGATGTATAGCACAACATATTTTCATT
Tzi8    TTCAACATTAAGATATTGTGTTCCATGTAACTATGATGTATAACACAACATATTTTCATT
```

```
B73       AACTGTTAAAATGGTTGACTGTATATGGGAAAGGTCAGCAGCTGAACTAATTTTTGTAGG
HP301     AACTGTTAAAATGGTTGACTGTATATGGGAAAGGTCAGCAGCTGAACTAATTTTTGTAGG
OH7B      AACTGTTAAAATGGTTGACTGTATATGGGAAAGGTCAGCAGGTGAACTAATTTTTGTAGG
Tzi8      AACTGTTAAAATGGTTGACTGTATATGGGAAAGGTCAGCAGCTGAACTAATTTTTGTAGG
                                                      ****************
                                                        Acceptor vi  ⬆

B73       CAACTCTCATTGTTGAGAACATGCACCTTCATATACTTCCGACAATGAACCCTGATGGGT
HP301     CAACTCTCATTGTTGAGAACATGCACCTTCATATACTTCCGACAATGAACCCTGATGGGT
OH7B      CAACTCTCATTATTGAGAACATGCACCTTCATATA-TTACGACAATGAACCCTGATGGGT
Tzi8      CAACTCTCATTGTTGAGAACATGCACCTTCATATACTTCCGACAATGAACCCTGATGGGT
          *********** **

B73       TTGCTCTTAGATGGCATGGTAATGCAAACAATATTGATCTCAACAGGGATTTTCCTGACC
HP301     TTGCTCTTAGATGGCATGGTAATGCAAACAATATTGATCTCAACAGGGATTTTCCTGACC
OH7B      TTGCTCTTAGATGGCGTGGTAATGCAAACAATATTGTTCTCAACAGGGATTTTCCTGACC
Tzi8      TTGCTCTTAGATGGCATGGTAATGCAAACAATATTGATCTCAACAGGGATTTTCCTGACC
                                                         *************

B73       AA-GTGAGTTACTTCTTTACATTACACCTCTTTTCCTTATGGAAGCTTCTACTTCCCTTT
HP301     AA-GTGAGTTACTTCTTTACATTACACCTCTTTTCCTTATGGAAGCTTCTACTTCCCTTT
OH7B      AAAGTGAGTTACTTCTTTACATTACACCTCTTTTCCTTATGGAAGCTTCTACTTCCCTTT
Tzi8      AA-GTGAGTTACTTCTTTACATTACACCTCTTTTCCTTATGGAAGCTTCTACTTCCCTTT
          ** **************
            ⬆ Donor vii

B73       TTGTACTCATTTTTT-CTTGTAGCCATT-CAATTGACACTAGCCGAAATTGA-AGTTCTT
HP301     TTGTACTCATTTTTT-CTTGTAGCCATT-CAATTGACACTAGCCGAAATTGA-AGTTCTT
OH7B      TTGTACTCATTTTTTTCTTGTAGCCATTTCAATTGACACTAGCTGAAATTGACAGTTCTT
Tzi8      TTGTACTCATTTTTT-CTTGTAGCCATT-CAATTGACACTAGCCGAAATTGA-AGTTCTT

B73       CCCCGTTAACAACGATATTGACTACAGACAGCCTGAAACTAGAGCCATTATGAATTGGGT
HP301     CCCCGTTAACAACGATATTGACTACAGACAGCCTGAAACTAGAGCCATTATGAATTGGGT
OH7B      CCCCGTTAACAACGATATTGACTACAGACAGCCTGAAACTAGAGCCATTATGAATTGGGT
Tzi8      CCCCGTTAACAACGATATTGACTACAGACAGCCTGAAACTAGAGCCATTATGAATTGGGT
                       *****************************
                                     ⬆ Donor vii

B73       AAAGCAAGAACACTTCACGACTTCTGCTAGCTTGCATGGGGTAACTGTTCTCACGTACTG
HP301     AAAGCAAGAACACTTCACGACTTCTGCTAGCTTGCATGGGGTAACTGTTCTCACGTACTG
OH7B      AAAGCAAGAACACTTCACGGCTTCTGCTAGCTTGCATGGGGTAACTGTTCTCACGTACTG
Tzi8      AAAGCAAGAACACTTCACGACTTCTGCTAGCTTGCATGGGGTAACTGTTCTCACGTACTG
                                   ******************************
                                                ⬆ Donor viii

B73       TAGCTATAATATCAGTTATCCTGTTGAAATGAGATCCTATAGTTTTCTCTTATTTGATTT
HP301     TAGCTATAATATCAGTTATCCTGTTGAAATGAGATCCTATAGTTTTCTCTTATTTGATTT
OH7B      TAGCTATAATATCAGCTATCCTGTTGAAATGAGATCCTATAGTTTTCTCTTATTTGATTT
Tzi8      TAGCTATAATATCAGTTATCCTGTTGAAATGAGATCCTATAGTTTTCTCTTATTTGATTT
                                                                 *****

B73       TGTTCTTCAGGGGGCTCTTGTTGCCAACTATCCATGGGATGGAACTAGAGACACAAGGTG
HP301     TGTTCTTCAGGGG-CTCTTGTTGCCAACTATCCATGGGATGGAACTAGAGACACAAGGTG
OH7B      TGTTCTTCAAGGGGCTCTTGTTGCCAACTATCCATGGGATGGAACTAGAGACACAAGGTG
Tzi8      TGTTCTTCAGGGGGCTCTTGTTGCCAACTATCCATGGGATGGAACTAGAGACACAAGGTG
          ********* *** ***********                 ******************
                   ⬆ Acceptor viii                       Donor ix  ⬆

B73       ATTT-CTTCTTCTACTTAATTTCAAGTT-----------TCTCAATTAATAGTGTTGGGT
HP301     ATTT-CTTCTTCTACTTAATTTCAAGTT-----------TCTCAATTAATAGTGTTGGGT
OH7B      ATTT-CTTCTTCTACTTAATTTCAAGTTAATTTCAAGTTTCTCAATTAATAGTGTTGGGT
Tzi8      ATTTTCTTCTTCTACTTAATTTCAAGTT-----------TCTCAATTAATAGTGTTGGGT
          **** ********
```

```
B73      GTGTTTGGTTGCGGGACGGCCAGGACAAGGATGTCCCCTGGCGTCCTCTCTCGTTCCTCC
HP301    GTGTTTGGTTGCGGGACGGCCAGGACAAGGATGTCCCCTGGCGTCCTCTCTCGTTCCTCC
OH7B     GTGTTTGGTTGCGGGACGGCCAGGATAGGGATGTCCCCTAGTGCCCTCTCTCGTTCCTCC
Tzi8     GTGTTTGGTTGCGGGACGGCCAGGACAAGGATGTCCCCTGGCGTCCTCTCTCGTTCCTCC

B73      AATTTTGAGGGATAACTGGGGACAACACTGGGATAGTCATGTCCCAACTCTTGACCCTGA
HP301    AATTTTGAGGGATAACTGGGGACAACACTGGGATAGTCATGTCCCAACTCTTGACCCTGA
OH7B     AATTTTGAGGGATAACTGGGGACAACACTGGGATAGTCATGTCCCAACTCTTGACCCTGA
Tzi8     AATTTTGAGGGATAACTGGGGACAACACTGGGATAGTCATGTCCCAACTCTTGACCCTGA

B73      ACCAAACAACCTTATTTGAGGGATCGTCCCATCCCGTCCCGTCCTGTCCTATCATTGCAA
HP301    ACCAAACAACCTTATTTGAGGGATCGTCCCATCCCGTCCCGTCCTGTCCTATCATTGCAA
OH7B     ACCAAACAACCTTATTTGAGGGATCGTCACATCCCGTCCTGTCCCGTCCTGTCATTGCAA
Tzi8     ACCAAACAACCTTATTTGAGGGATCGTCCCATCCCGTCCCGTCCTGTCCTATCATTGCAA

B73      CCAAACGCATCCTAAAACTTCTCATGAAGTATTGCCCAAAAACTATCTTCCCATGGTTCT
HP301    CCAAACGCATCCTAAAACTTCTCATGAAGTATTGCCCAAAAACTATCTTCCCATGGTTCT
OH7B     CCAAACGCATCCTAAAACTTCTCATGAAGTATTGCCCAAAAACTATCTTCCCATGGTTCT
Tzi8     CCAAACGCATCCTAAAACTTCTCATGAAGTATTGCCCAAAAACTATCTTCCCATGGTTCT

B73      AATGGTTATTTGAAGTTTGGATTGCAAGTAGGGGCCTTAATATTTCCTAATAAGATGTTT
HP301    AATGGTTATTTGAAGTTTGGATTGCAAGTAGGGGCCTTAATATTTCCTAATAAGATGTTT
OH7B     AATGGTTATTTGAAGTTTGGATTGCAAGTAGGGGCCTTAATATGTCCTAATAAGATGTTT
Tzi8     AATGGTTATTTGAAGTTTGGATTGCAAGTAGGGGCCTTAATATTTCCTAATAAGATGTTT

B73      CCACTTTCCACTGTTAGTTGCATTTTTATTCTTTATATGAAAATGGTTTGTTTTACTATC
HP301    CCACTTTCCACTGTTAGTTGCATTTTTATTCTTTATATGAAAATGGTTTGTTTTACTATC
OH7B     CCACTTTCCACTGTTAGTTGCATTTTTATTCTTTATATGAAAATGGTCTGTTTTACTATC
Tzi8     CCACTTTCCACTGTTAGTTGCATTTTTATTCTTTATATGAAAATGGTTTGTTTTACTATC

B73      ACTGCCCTCTTGACACCTAAACAAAATTTTGACTCATTTTGCTCTCCCAAACTATTTAAT
HP301    ACTGCCCTCTTGACACCTAAACAAAATTTTGACTCATTTTGCTCTCCCAAACTATTTAAT
OH7B     ACTGCCCTCTTGACACCTAAACAAAATTTTGACTCATTTTGCTCTCCCAAACTATTTAAT
Tzi8     ACTGCCCTCTTGACACCTAAACAAAATTTTGACTCATTTTGCTCTCCCAAACTATTTAAT

B73      CTGGGCCAGTTCACCCCCTAATGAAAATATTATATTTCCTTCTCCATGTTCAAATATTGA
HP301    CTGGGCCAGTTCACCCCCTAATGAAAATATTATATTTCCTTCTCCATGTTCAAATATTGA
OH7B     CTGGGCCAGTTCACCCC-TAATGAAAATATTCTATTTCCTTCTCCATATTCAAATACTGA
Tzi8     CTGGGCCAGTTCACCCCCTAATGAAAATATTATATTTCCTTCTCCATGTTCAAATATTGA

B73      TATACTTTTGTATCATAAGGTCTGTTGGCATCTATCATTTTGCAAATTTGCACCAGCAAC
HP301    TATACTTTTGTATCATAAGGTCTGTTGGCATCTATCATTTTGCAAATTTGCACCAGCAAC
OH7B     TATACTTTTGTATCATAAGGTCTGTTGGCATCTATCATTTTGCAAATTTGCACCAGCAAC
Tzi8     TATACTTTTGTATCATAAGGTCTGTTGGCATCTATCATTTTGCAAATTTGCACCAGCAAC
             ******************************
                            ⬆ Acceptor ix

B73      AGAACATGTACATGGCGAAGACAAATCATTAGGTGGCGACTTTTTTCTCACCACGGTAAC
HP301    AGAACATGTACATGGCGAAGACAAATCATTAGGTGGCGACTTTTTTCTCACCACGGTAAC
OH7B     AGAACATGTACATGGCAAAGACAAATCATTAGGTGGTGACTTTTTTCTCACCACGGTAAC
Tzi8     AGAACATGTACATGGCGAAGACAAATCATTAGGTGGCGACTTTTTTCTCACCACGGTAAC
```

```
B73      ACTGAATTTCATTATGGCAGCAACAAAGTTACAATATTCAGGTTTCATTAGGTGGTGTTT
HP301    ACTGAATTTCATTATGGCAGCAACAAAGTTACAATATTCAGGTTTCATTAGGTGGTGTTT
OH7B     ACTGAATTTCATTATGGCAGCAACAAAGTTACAATATTCAGGTTTCATTAGGTGGTGTTT
Tzi8     ACTGAATTTCATTATGGCAGCAACAAAGTTACAATATTCAGGTTTCATTAGGTGGTGTTT

B73      TTTACTAATATTTGAAGAGTTTGAGTTGGGCGACAGGGGGG-TATTGCCAGCAACTGGTT
HP301    TTTACTAATATTTGAAGAGTTTGAGTTGGGCGACAGGGGGG-TATTGCCAGCAACTGGTT
OH7B     CTTACTAATATTTGAAGAGTTTGAGTTGGGCGACAGGGGGGGTATTGCCAGCAACTGGTT
Tzi8     TTTACTAATATTTGAAGAGTTTGAGTTGGGCGACAGGGGGG-TATTGCCAGCAACTGGTT

B73      AATGAACTGTTATGGCTGAAGTGAACCATTAATATAGAGTTTGAGTTATTCTGTGTTGTC
HP301    AATGAACTGTTATGGCTGAAGTGAACCATTAATATAGAGTTTGAGTTATTCTGTGTTGTC
OH7B     AATGAATTGCTATGGCTGAAGTGAACCATTAATGTAGAGTTTGAGTTATTCTGTGTTGTC
Tzi8     AATGAACTGTTATGGCTGAAGTGAACCATTAATATAGAGTTTGAGTTATTCTGTGTTGTC

B73      ACAGACAACCGTATTTACACACACAGTATCTCGCCCGCTCCTCCAAGTCTCCCTCTCTCA
HP301    ACAGACAACCGTATTTACACACACAGTATCTCGCCCGCTCCTCCAAGTCTCCCTCTCTCA
OH7B     ACAGATAACCGTATTTACACACACAGTATCTCGCCTGCTCCTCCAAGTCTCCCTCTCTCA
Tzi8     ACAGACAACCGTATTTACACACACAGTATCTCGCCCGCTCCTCCAAGTCTCCCTCTCTCA

B73      ATATAAGCCTATTTCTGAATTCTATTTGTGGTCGCTTTCTTTG-ATTTGCTGATTGTCAC
HP301    ATATAAGCCTATTTCTGAATTCTATTTGTGGTCGCTTTCTTTG-ATTTGCTGATTGTCAC
OH7B     ATATAAGCCTATTTCTGAATTCTATTTATGGTCGCTTTCTTTGTATTTGCTGATTGTCAC
Tzi8     ATATAAGCCTATTTCTGAATTCTATTTGTGGTCGCTTTCTTTG-ATTTGCTGATTGTCAC

B73      CACTGGTTTTTAGGGCTCAATATGACTTTGTTTCACATTGTAACATCCTCAACAATGATT
HP301    CACTGGTTTTTAGGGCTCAATATGACTTTGTTTCACATTGTAACATCCTCAACAATGATT
OH7B     CACTGGTTTTTAGGGCTCAATATGACTTTGTTTCACATTGTAACATCCTCAACAATGATT
Tzi8     CACTGGTTTTTAGGGCTCAATATGACTTTGTTTCACATTGTAACATCCTCAACAATGATT

B73      TTACTTCTTACAACAAACACTACTACGGATGTCCTGATGATAAGACATTCCAGCACATGG
HP301    TTACTTCTTACAACAAACACTACTACGGATGTCCTGATGATAAGACATTCCAGCACATGG
OH7B     TTACTTCTTACAGCAAACACTACTACAGATGTCCTGGTGATAAGACATTCCGGCACATGG
Tzi8     TTACTTCTTACAACAAACACTACTACGGATGTCCTGATGATAAGACATTCCAGCACATGG

B73      CATCTGTGTATAGTCGGTCTCACTATAACATGTCTTTGAGCAAAGAATTTGAAGGAGGGA
HP301    CATCTGTGTATAGTCGGTCTCACTATAACATGTCTTTGAGCAAAGAATTTGAAGGAGGGA
OH7B     CATCTGTGTATAGTCGGTCTCACTATAACATGTCTTTGAGCAAAGAATTTGAAGGAGGGA
Tzi8     CATCTGTGTATAGTCGGTCTCACTATAACATGTCTTTGAGCAAAGAATTTGAAGGAGGGA

B73      TAACAAATGGAGCATTCTGGTAAACAGACAATAGCAAGTTCATATTTCATAATTCATATG
HP301    TAACAAATGGAGCATTCTGGTAAACAGACAATAGCAAGTTCATATTTCATAATTCATATG
OH7B     TAACAAATGGAGCATTCTGGTAAACAAACAATAGCAAGTTCATATTTCATAATTCATATG
Tzi8     TAACAAATGGAGCATTCTGGTAAACAGACAATAGCAAGTTCATATTTCATAATTCATATG
             ********************** *******
                            ↑ Donor x

B73      AGATTTCCTTTATCTAGGTACTACGTAGGTTGTGTATTTGTTGGCTTTGAATCAAACAAC
HP301    AGATTTCCTTTATCTAGGTACTACGTAGGTTGTGTATTTGTTGGCTTTGAATCAAACAAC
OH7B     AGATTTCCTTTATCTAGGTACTAGGTAGGTTGTGTATTTGTTGGCTTTGAATCAAACAAC
Tzi8     AGATTTCCTTTATCTAGGTACTACGTAGGTTGTGTATTTGTTGGCTTTGAATCAAACAAC
```

```
B73     ATCATTGCTTGTGGTCCAGGTACCCAATATATGGTGGTATGCAAGACTGGAACTATATAC
HP301   ATCATTGCTTGTGGTCCAGGTACCCAATATATGGTGGTATGCAAGACTGGAACTATATAC
OH7B    ATCATCGCTTGTGGTCCAGGTACCCAATATATGGTGGTATGCAGGACTGGAACTATATAC
Tzi8    ATCATTGCTTGTGGTCCAGGTACCCAATATATGGTGGTATGCAAGACTGGAACTATATAC
            * ****************************
                               Acceptor x

B73     ATGGAGGCTGCTTTGAGTTAACTCTGGAGATTAGTGACACAAAGTGGCCAAAAGCAGATG
HP301   ATGGAGGCTGCTTTGAGTTAACTCTGGAGATTAGTGACACAAAGTGGCCAAAAGCAGATG
OH7B    ATGGAGGCTGCTTTGAGTTAACTCTGGAGATTAGTGACACAAAGTGGCCAAAAGCAGATG
Tzi8    ATGGAGGCTGCTTTGAGTTAACTCTGGAGATTAGTGACACAAAGTGGCCAAAAGCAGATG
                                                       *************

B73     AGGTACCTTTGCATCGAGTTAGGTTCCACTTCTACTGTTGCAGACCCAGACCATTTTATA
HP301   AGGTACCTTTGCATCGAGTTAGGTTCCACTTCTACTGTTGCAGACCCAGACCATTTTATA
OH7B    AGGTACCTTTGCATCGAGTTAGGTTCCACTTCTACTGTTGCAGACCCAGACCATTTTATA
Tzi8    AGGTACCTTTGCATCGAGTTAGGTTCCACTTCTACTGTTGCAGACCCAGACCATTTTATA
        *****************
           Donor xi

B73     ATTATTCTTCTGTAATGTTGTTTATCCTAACATTTTCCTACAGCTTCCTATCATCTGGGA
HP301   ATTATTCTTCTGTAATGTTGTTTATCCTAACATTTTCCTACAGCTTCCTATCATCTGGGA
OH7B    ATTATTCTTCTGTAATGTTGTTTATCCTAACATTTTCCTACAGCTTCCTATCATCTGGGA
Tzi8    ATTATTCTTCTGTAATGTTGTTTATCCTAACATTTTCCTACAGCTTCCTATCATCTGGGA
                                    ******************************
                                                  Acceptor xi

B73     ACACAGTAGGATGAGTATGCTCAATCTTCTAGCAAGCCTAATAAAGGTAATATGTTTCCA
HP301   ACACAGTAGGATGAGTATGCTCAATCTTCTAGCAAGCCTAATAAAGGTAATATGTTTCCA
OH7B    ACACAATAGGATGAGTATGCTCAATCTTCTAGCAAGCCTAATAAAGGTAATATGTTTCCA
Tzi8    ACACAGTAGGATGAGTATGCTCAATCTTCTAGCAAGCCTAATAAAGGTAATATGTTTCCA
                                       *****************************
                                                    Donor xii

B73     CAGTAGTCTCTTTATGCACTGCACAGGGTGATTTTTGCACCAATTGAATGGTTAGTGAAT
HP301   CAGTAGTCTCTTTATGCACTGCACAGGGTGATTTTTGCACCAATTGAATGGTTAGTGAAT
OH7B    CAATAGTCTCTTTATGCACTGCACAAGGTGATTTTTGCACCAATTGAATGGTTAGTGAAT
Tzi8    CAGTAGTCTCTTTATGCACTGCACAGGGTGATTTTTGCACCAATTGAATGGTTAGTGAAT
        *

B73     TGAGGCACTTGGTAGTTGGTTGCATATGTTTAGACTAGGGTCTGGATCATGTACTGTAAC
HP301   TGAGGCACTTGGTAGTTGGTTGCATATGTTTAGACTAGGGTCTGGATCATGTACTGTAAC
OH7B    TGAGGCACTTGGTAGTTGGTTGCATATGTTTAGACTAGGATCTGGATCATGTATTGTAAC
Tzi8    TGAGGCACTTGGTAGTTGGTTGCATATGTTTAGACTAGGGTCTGGATCATGTACTGTAAC

B73     TATTCGATTTTGACGCCTGTAGATTGCAAGAACGGGACTGCTAAGCAAGCTAAGATTTCA
HP301   TATTCGATTTTGACGCCTGTAGATTGCAAGAACGGGACTGCTAAGCAAGCTAAGATTTCA
OH7B    TGTTCGATTTTGACGCCTGCAGATTGCAAGAACAGGGCTGCTAAGTAAGCTAAGATTTCA
Tzi8    TATTCGATTTTGACGCCTGTAGATTGCAAGAACGGGACTGCTAAGCAAGCTAAGATTTCA
               ************ ************* **
                              Acceptor xii

B73     ATGGCCTATTCCTTCATACACAGGCCATTGAGCGACGAGGAACGCCGT------------
HP301   ATGGCCTATTCCTTCATACACAGGCCATTGAGCGACGAGGAACGCCGT------------
OH7B    ATGGCCTATTCCTTCATACATAGGCCATTGAGCGACGAGGAACGCCGCATCAGCACTCCC
Tzi8    ATGGCCTATTCCTTCATACACAGGCCATTGAGCGACGAGGAACGCCGT------------

B73     ------GTCACTTGCAATGACCAGAGACGGGAGGTCTTCGTTGCCCAGGACATCGCTAAC
HP301   ------GTCACTTGCAATGACCAGAGACGGGAGGTCTTCGTTGCCCAGGACATCGCTAAC
OH7B    GTTGTGGTCACTTGCAATGACCAGAGACGGGAGGTCTCCGTTGCCCAGAACATCGCTAAC
Tzi8    ------GTCACTTGCAATGACCAGAGACGGGAGGTCTTCGTTGCCCAGGACATCGCTAAC
```

```
B73     AAGGAAATTGACCGCACCGTTGCATTCGACAAGGTAAGATGGATTAGCAAATAATATTGT
HP301   AAGGAAATTGACCGCACAAG-------GGCGAATTCCAGCACACTGGCGGCCGTTACTAG
OH7B    AAGGAAATTGACCGCACCGTTGCATTCGACAAGGTAAGATGGATTAGCAAATAATATTGT
Tzi8    AAGGAAATTGACCGCACAAG-------GGCGAATTCT-GCAGA-TATCCATCAC-ACTGG

B73     TTGACATTTAGGTTTTTATATTTTATATAATACACTATTAGGTGAGTGCCTGTGCGTTGC
HP301   TGG--ATCCGAGCTCG------------GATCCACTAGTAA-CGGCCGCCAGTGTGCTG-
OH7B    TTGACATTTAGGTTTTTATATTTTATATAATACACTATTAGGTGAGTGCCTGTGCGTTGC
Tzi8    CGGCCGCTCGAGCATG------------CATCTAGAGGGCC-CAATTCGCCCTATAGTG-
```

Figure S3 Alignment of *Helitron Hel1-333* sequence between the maize inbred lines. The sequence alignment displays the splice junctions i-xii of *Hel1-33*3 in B73 that are shown in figure 4. The blue and gray shaded sequences flanking the splice sites represent exons and introns, respectively. Conserved flanking sequences between all the inbred lines are indicated by asterisks beneath the highlighted regions, while the dashed lines fill the gaps in alignment. The inbred lines are indicated on the left.
